# Engineered bacteria reprogram tumor microenvironment *via* cell senescence and neutrophil extracellular traps degradation

**DOI:** 10.1016/j.apsb.2026.01.030

**Published:** 2026-01-28

**Authors:** Wanfa Dong, Chenyang Li, Jiqiang Lu, Lin Weng, Yicong Xu, Min Xu, Peiqi Li, Yanhui Wu, Zixuan Shan, Pengyou Shang, Liangliang Dai, Tao Zhang, Yanlong Jia, Tianyun Wang, Wenjie Ren, Ping Lu, Xiao Chen, Zichun Hua

**Affiliations:** a School of Biopharmacy, China Pharmaceutical University, Nanjing 211198, China; b The State Key Laboratory of Pharmaceutical Biotechnology, College of Life Sciences, Nanjing University, Nanjing 210023, China; c Faculty of Pharmaceutical Sciences, Xinxiang Medical University, Xinxiang 453003, China; d Changzhou High-Tech Research Institute of Nanjing University and Jiangsu TargetPharma Laboratories Inc., Changzhou 213164, China

**Keywords:** Neutrophil, Oncolytic bacteria, Cellular senescence, NETosis, T cell exhaustion, Staphylococcal nuclease, Cancer immunotherapy, VNP20009

## Abstract

Attenuated *Salmonella* VNP20009 (VNP) shows promising anti-cancer therapeutic potential. Limited understanding of its anti-tumor mechanism has hindered broader clinical application. Recent studies have reported cellular senescence is involved in tumor progression; however, its critical role in VNP therapy remains elusive. Our study revealed that VNP exerts anti-tumor growth and anti-angiogenesis effects by inducing cellular senescence in tumor cells and vascular endothelial cells. While VNP-induced senescence inhibits tumor growth, it concurrently promotes neutrophil extracellular traps (NETs), which paradoxically enhance tumor progression. To address this challenge, we engineered VNP-SNase, a novel variant capable of releasing the DNA-degrading enzyme *Staphylococcus aureus* nuclease directly within tumors. VNP-SNase significantly inhibited NETs formation across multiple tumor types, effectively promoted anti-tumor immunity, and exhibited improved tumor suppression effects with enhanced biosafety. Our findings elucidate the critical role of cellular senescence in VNP therapy and propose targeting NETs as a strategic approach to enhance the efficacy of VNP-based cancer treatments.

## Graphical abstract

Attenuated *Salmonella* VNP20009 triggers senescence in tumor and endothelial cells to suppress tumor growth. However, it also induces neutrophil senescence and the formation of tumor-promoting neutrophil extracellular traps. The engineered strain degrades these traps, blocking their pro-tumor effects and enhancing antitumor efficacy.

**Figure:**
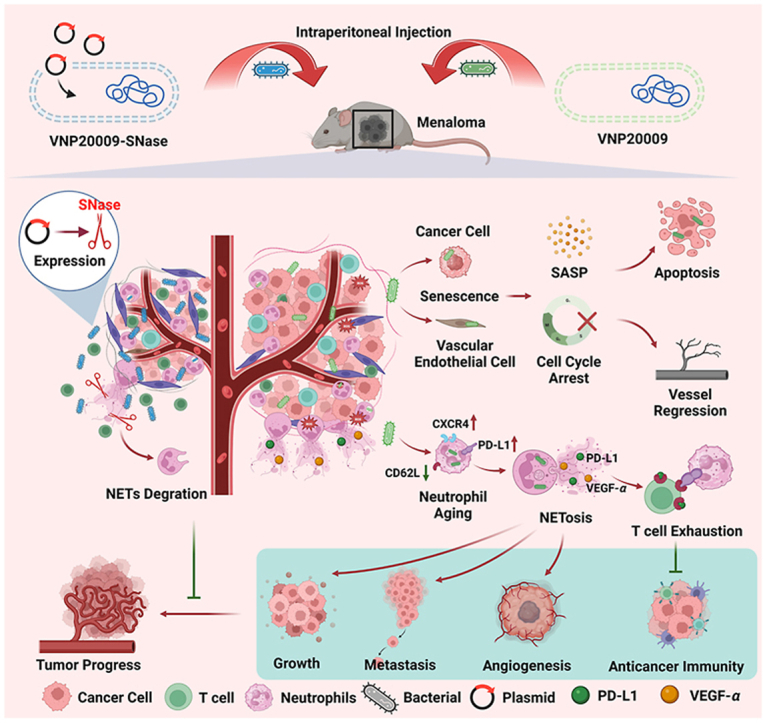

## 1 Introduction

VNP20009 (abbreviated as VNP, which lacks the purI and msbB genes) is an attenuated strain of *Salmonella* that has been extensively studied in preclinical research for cancer treatment^1^, demonstrating promising therapeutic results. The mechanisms underlying VNP’s anti-tumor effects have been extensively investigated. Previously published literature and our group’s research have shown that VNP exerts its effects through multiple complex pathways simultaneously, including direct induction of tumor cell apoptosis, immune activation, inhibition of angiogenesis, and metabolic disruption^1^, ^2^, ^3^, ^4^, ^5^. While VNP has been designated as the unique *Salmonella* strain to receive approval for Phase I clinical trials^6^, the results indicated that VNP monotherapy did not achieve satisfactory therapeutic outcomes. Therefore, further exploration of VNP’s anti-tumor mechanisms is crucial, as it will guide both the modification of VNP strains in basic research and the development of combination therapies in clinical settings.

Cellular senescence is characterized by cell cycle arrest, expression of cyclin-dependent kinase inhibitor proteins, elevated senescence-associated *β*-galactosidase activity, and the development of senescence-associated secretory phenotype (SASP)^7^^,^^8^. The role of cellular senescence in tumor progression is notably complex^9^^,^^10^. Several findings have demonstrated that senescence serves as a natural tumor suppression mechanism to arrest cell cycle and promote apoptosis^11^^,^^12^. However, SASP components, including pro-inflammatory factors, can remodel the tumor microenvironment (TME) and paradoxically promote tumor growth and metastasis^13^, ^14^, ^15^. For instance, neutrophil senescence induces the formation of neutrophil extracellular traps (NETs)^16^, which promote tumor progression and suppress anti-tumor immunity through multiple sophisticated mechanisms^17^, ^18^, ^19^.

Given the critical role of cellular senescence in tumor progression, cellular senescence has emerged as a novel potential target for anti-tumor therapy. For examples, the galactose-conjugated form of Navitoclax promotes further apoptosis in senescent lung cancer cells, thereby improving survival in a mouse model of lung cancer^20^; DNASE1L3 impedes tumor angiogenesis by affecting the SASP^21^; the combination of Palbociclib and Doxorubicin induces tumor cell senescence, thereby enhancing anti-tumor immunity^22^^,^^23^. These studies demonstrate that targeting cellular senescence can influence multiple tumor-related processes, including cell apoptosis, angiogenesis, and anti-tumor immunity. Considering that VNP similarly induces cell apoptosis, inhibits angiogenesis, and promotes anti-tumor immunity, we hypothesize that cellular senescence may also be involved in these VNP-mediated processes. However, the effects of VNP on senescence of various cells within the TME, and their subsequent impact on tumor progression and VNP therapeutic efficacy, remain uncertain.

In our study, we discovered that during melanoma treatment, VNP significantly induced senescence in both B16F10 tumor cells and human umbilical vein endothelial cells (HUVECs), effectively inhibiting tumor growth and angiogenesis. Given that VNP leads to massive recruitment of neutrophils in the TME, we examined VNP’s effects on neutrophils and found that it could also induce neutrophil senescence and promote NETs formation. Unexpectedly, these NETs further promoted tumor proliferation, metastasis, and angiogenesis. Moreover, NETs upregulated PD-L1 levels in the TME, resulting in cytotoxic T cell exhaustion and suppression of anti-tumor immunity. Based on these findings, we engineered a modified strain of VNP (VNP-SNase) capable of releasing *Staphylococcus aureus* nuclease (SNase), which specifically targets the DNA scaffold of intra-tumoral NETs. By disrupting intra-tumoral NETs, VNP-SNase demonstrated increased bacterial colonization in the TME and enhanced inhibition against tumor proliferation, metastasis, and angiogenesis. The modified strain also alleviated T cell exhaustion, subsequently boosting anti-tumor immunity. Additionally, VNP-SNase significantly suppressed lung metastasis in melanoma models. These findings establish VNP-SNase as a highly promising engineered bacterial therapeutic agent for cancer treatment. Our research not only expands our understanding of VNP’s mechanism of action but also provides innovative strategies for bacterial modification and combination therapy in cancer treatment.

## 2 Materials and methods

### 2.1 Animals and cell lines

Wild-type female C57BL/6J mice (6–8 weeks old) were purchased from Huachuang Sino Company, Nanjing, China. All animal experiments were conducted in accordance with the approved protocols of the Animal Protection and Use Committee of China Pharmaceutical University (2024-12-099). The cell lines B16F10, 4T1, HUVEC and Hepa1-6 were archived in our laboratory. B16F10 cells were cultured in RPMI 1640 medium (Thermo Fisher Scientific, MA, USA) supplemented with 10% FBS (Gibco, NY, USA). All cells were cultured at 37 °C in a humidified atmosphere containing 5% CO_2_. B16F10 cells (5.0 × 10^5^ cells/mouse) were subcutaneously injected into the right flank of C57BL/6J mice. When the tumor volume reached approximately 100 mm^3^, VNP (1.0 × 10^6^ CFU/mouse) and VNP-SNase strains (1.0 × 10^6^ CFU/mouse) were intraperitoneally injected once, while the SNase group received daily intraperitoneal injections (5 mg/kg). Tumor volume was measured and calculated daily using Eq. (1):

(1) *V* = 0.52 × *a* × *b*^2^

where *a* represents the long diameter and *b* the short diameter. The tumor doubling time (TDT) was calculated using Eq. (2):

(2) TDT = *t* × log_2_/log (*V*_*t*_/*V*_0_)

where *t* represents the time interval between tumor assessments in days, *V*_*t*_ represents the tumor volume at time *t*, and *V*_0_ represents the initial tumor volume. Body weight of the mice was monitored daily. On the day of euthanasia, whole blood was collected for complete blood count, and serum was analyzed for liver function markers, kidney function marker (Servicebio, Wuhan, China), and NETs markers (Zeweil, Nanjing, China). Organ indices were calculated from the weighed heart, liver, spleen, lungs, and kidneys, and tissue sections were embedded and stained with H&E for pathological analysis (Servicebio, Wuhan, China). Tumor sections were subjected to fluorescence immunostaining (Dreambio, Nanjing, China). A melanoma lung metastasis model was established by injecting 1.5 × 10^5^ B16F10 cells suspended in 100 μL PBS (Servicebio, Wuhan, China) into the tail vein of mice. Treatment was initiated on Day 5, and mice were euthanized on Day 23 for the enumeration of metastatic foci in the lungs. The rationale for selecting the 8-day experimental timeframe, commencing treatment on Day 7 post-inoculation and terminating on Day 15, was grounded in several critical observations. Specifically, Day 7 was identified as the optimal initiation point for intervention, as it corresponds to the time when 4T1 tumors typically become palpable, with volumes ranging from approximately 50 to 100 mm^3^. This ensures uniform tumor establishment across all experimental groups prior to the commencement of treatment. Additionally, the chosen 8-day treatment duration was informed by two key factors: firstly, significant treatment effects become distinctly discernible in tumor growth trajectories by Day 15, and secondly, this timeframe aligns with the experimental timeline of our parallel melanoma study, thereby maintaining methodological consistency across related research endeavors. Serum samples were analyzed for NETs detection (Zeweil, Nanjing, China). Kaplan–Meier survival curves were plotted.

### 2.2 Cell apoptosis and cell cycle detection

B16F10 cells and HUVECs (3.0 × 10^5^) were seeded in wells of a cell culture plate (Jetbio, Guangzhou, China) and allowed to adhere overnight. The cells were then co-cultured with the VNP strain for 0, 2, 4, 6, 8, and 10 h at a multiplicity of infection (MOI) of 100. After harvesting the cells, they were washed and resuspended in 100 μL of 1 × binding buffer. Cell cycle and apoptosis assays were performed using the Cell Cycle Detection Kit (Beyotime, Shanghai, China) and the Cell Apoptosis Detection Kit (Yeasen, Shanghai, China), followed by incubation in the dark and on ice for 30 min. To assess the survival of primary neutrophils, tumor masses from the PBS and VNP groups were processed into single-cell suspensions, and primary neutrophils were isolated using MojoSort Mouse Neutrophil Isolation Kit (BioLegend, CA, USA). Apoptosis was assessed at various time points during the *in vitro* culture of these cells. The stained cells were analyzed using a flow cytometer (BD LSRFortessa, USA), and the data were processed using FlowJo VX software.

### 2.3 Neutrophil isolation

Wild-type female C57BL/6J mice were euthanized and their tibiae and femurs were removed and stripped of muscle tissue. Bone marrow was flushed from the bones using a 1 mL syringe (BD, NJ, USA), and red blood cells were lysed (Beyotime, Shanghai, China). Neutrophils were isolated using the MojoSort Mouse Neutrophil Isolation Kit (BioLegend, CA, USA). For intra-tumoral neutrophil isolation, tumor tissue was digested using digestion medium containing 1 mg/mL collagenase I, 1 mg/mL, and 200 μg/mL DNase I (Yeasen, Shanghai, China) at 37 °C for 30 min, followed by lysis of red blood cells and filtration of the cell suspension through a 200 μm filter. Neutrophils were then isolated from the tumor following the same protocol.

### 2.4 NETs production *in vitro*

Purified neutrophils were resuspended in RPMI 1640 medium and plated at a density of 2.0 × 10^6^ cells in each well of a 6-well plate. VNP was co-incubated with neutrophils at an MOI of 100:1 at 37 °C and 5% CO_2_ for 4 h. The culture medium was then aspirated, and the mixture was centrifuged at 4 °C, 100,000×*g* for 10 min (Beckman, CA, USA). The supernatant, containing NETs, was used for further experiments.

### 2.5 Cell viability assay

After resuspending tumor cells (1.0 × 10^3^) in 100 μL of culture medium, the cells were seeded onto a 96-well plate (Jetbio, Guangzhou, China) and incubated overnight at 37 °C. The following day, the cells were treated with SNase, NETs, and SNase + NETs. Subsequently, 10 μL of CCK-8 solution (Beyotime, Shanghai, China) was added to each well at 24, 48, and 72 h, followed by a 2 h incubation at 37 °C. The absorbance at 450 nm was then measured using a microplate reader (BioTek Instruments, VT, USA) to determine cell viability. Additionally, Tumor cells (1.0 × 10^3^) were seeded in a 96-well plate for overnight culture. The following day, neutrophils were added to each well at a 1:1 ratio, and cell viability of the B16F10 cells was measured at 24, 48, and 72 h.

### 2.6 Wound healing assay

Tumor cells (3.0 × 10^5^) were resuspended in 500 μL of RPMI 1640 medium and seeded onto a 24-well plate. The plate was then placed in a 37 °C, 5% CO_2_ cell culture incubator (Thermo Fisher Scientific, MA, USA) for 24 h to allow cell adhesion. A vertical scratch was created on the bottom surface of each well using a sterile 20 μL pipette tip (Corning, Shanghai, China) to induce a wound. The cells were subsequently treated with NETs, and the wound width was monitored and measured at 36 h to assess the impact of NETs on cell migration.

### 2.7 Trans-well assay

Cells in the logarithmic growth phase were suspended in 100 μL of RPMI 1640 medium and seeded onto the upper chamber of a Trans-well (Corning, Shanghai, China) insert with an 8-μm pore size. They were then cultured in the medium containing NETs without FBS. The lower chamber was filled with 800 μL of RPMI 1640 medium supplemented with 10% serum. At different hours post-culture, cells in the upper chamber were gently removed using a cotton swab (Kanmine, Jinhua, China). The cells that had migrated to the underside were fixed with 4% paraformaldehyde (Servicebio, Wuhan, China) for 30 min, stained with 1% crystal violet (Solarbio, Beijing, China) for 20 min, washed three times with PBS, air-dried, and then imaged using a microscope (Leica, Wetzla, Germany).

### 2.8 HUVECs tube formation assay

Fifty microliters of Matrigel (BD Biosciences, NJ, USA) were plated in a pre-cooled 96-well plate and incubated at 37 °C for 30 min. HUVECs at a density of 3.0 × 10^4^ were resuspended in 100 μL of DMEM medium (Thermo Fisher Scientific, NY, USA) and DMEM medium enriched with NETs, respectively. The HUVECs were then seeded into the 96-well plate, cultured for 12 h in a 37 °C, 5% CO_2_ incubator, and the tube formation was observed. For the experiment to assess the inhibition of HUVECs tube formation by VNPs, 3.0 × 10^4^ HUVECs were resuspended in 100 μL of DMEM medium, and VNPs were added at an MOI of 100:1 (VNP: HUVECs). After co-culturing for 12 h, gentamicin at a concentration of 50 μg/mL was added, and the tube formation was observed under a microscope (Leica, Wetzla, Germany) 2 h later.

### 2.9 RT-PCR

Total RNA was extracted using Trizol reagent (Invitrogen, CA, USA). 1 μg RNA was reverse transcribed into cDNA using a cDNA synthesis kit (Vazyme, Nanjing, China). Relative mRNA levels were quantified using a one-step RT-PCR SYBR Green kit (Vazyme, Nanjing, China). Primers were synthesized by Sangon Biotech (Shanghai, China), and their sequences are provided in Supporting Information Table S1.

### 2.10 Co-culture experiment of neutrophils and CD8^+^ T cells

On Day 1, CD8^+^ T cells were purified using magnetic beads (STEMCELL, VAN, Canada) and stimulated with 25 μg/mL CD3/CD28 T cell activator and 50 μg/mL recombinant human IL-2 for 48 h. On Day 3, peripheral blood neutrophils from mice were isolated and purified using MojoSort Mouse Neutrophil Isolation Kit (BioLegend, CA, USA), then added to activated CD8^+^ T cells in a 12-well plate at a 1:1 ratio and co-cultured for 48 h. After co-culture, mean fluorescence intensity (MFI) of exhaustion markers PD-1 and TIM-3 in CD8^+^ T cells were analyzed using FlowJo (v.10.4). For detecting *Pdcd1* and *Havcr2* mRNA levels in CD8^+^ T cells, the post-co-culture cells were sorted using CD8^+^ T cell selection, and RNA was extracted for q-PCR analysis. To investigate the effect of NETs on exhausted CD8^+^ T cells, activated CD8^+^ T cells were co-cultured with or without NETs-containing medium, and subsequent experimental steps were performed as described above.

### 2.11 Reactive oxygen species (ROS) measurement

Bone marrow-derived neutrophils were co-incubated with VNP at a multiplicity of infection (MOI) of 1:100 for 1, 2, or 3 h. ROS levels in these cells were measured using a ROS assay kit (Solarbio, Beijing, China). B16F10 cells and HUVECs were co-incubated with VNP at an MOI of 100:1 for various time points, after which they were treated with gentamicin (50 μg/mL, Sinoway, Xiamen, China) for 1 h. ROS levels in these cells were also determined using the ROS assay kit.

### 2.12 Neutrophil chemotaxis assay

Neutrophils (1.0 × 10^5^) were plated in 3415 Corning *Trans*-wells® with a 3-μm pore size. SNase, supernatant containing NETs, or a combination of both was added to the lower chamber, followed by incubation for 12 h. The fluid from the lower chamber was collected, and the neutrophils were enumerated using BD LSRFortessa. The chemotaxis ratio was then calculated.

### 2.13 Neutrophil depletion

To deplete neutrophils, anti-Ly6G antibody (BioXCell, NH, USA) and anti-mouse IgG2b antibody (BioXCell, NH, USA) were injected intraperitoneally one day before VNP treatment, with daily injections of 100 μg/mouse. Peripheral blood and intra-tumoral CD45^+^CD11b^+^Ly6G^+^ cell proportions were assessed at the end of the animal experiments.

### 2.14 RNA sequencing

Transcriptome sequencing of B16F10 cells (PBS, VNP (MOI 100, 8 h) groups) was performed by Oebiotech (Shanghai, China). The DEGs were selected by integrating both the *P* value and fold change the of each gene (*P* value < 0.05, absolute log_2_ (fold change) ≥1), following the GO and Reactome enrichment analysis.

### 2.15 Bacterial strains and plasmid construction

VNP20009, abbreviated as VNP, VNP-SNase, transformed with a plasmid expressing SNase under the control of a J23150 promoter, was tagged with an HA tag at the N-terminus of SNase to facilitate subsequent detection, VNP-RFP, expressing RFP under the control of a J23100 promoter and VNP-NC, transformed by an empty plasmid were used in this study. All plasmids were constructed using the ClonExpress II MultiS One Step Cloning Kit (Vazyme, Nanjing, China). The VNP strains were electro-transformed with the plasmids as described previously.

### 2.16 Bacterial growth curve determination

The OD_600_ of the VNP strains was adjusted to 1.0. Subsequently, 10 μL of the bacterial suspension was added to 0.5 mL of LB medium and incubated in a microplate reader (BioTek Instruments, VT, USA) at 37 °C for 28 h, with measurements of OD_600_ taken every 30 min for the generation of a growth curve.

### 2.17 VNP distribution *in vivo*

On Day 9 post-treatment, mice were euthanized, and their tumors and other organs, including the liver and spleen, were harvested in tubes and homogenized (Servicebio, Wuhan, China). The homogenate was diluted with sterile PBS to the appropriate concentration, plated on LB agar plates, and incubated for 16 h for bacterial colony formation. The bacterial colonies on the plates were then counted to determine the distribution of bacteria within the organs. To assess the *in vivo* pharmacokinetics of VNP-SNase in mice, major organs and whole-blood samples were collected at predefined intervals post-administration. Tissues and blood were homogenized or diluted in sterile saline at appropriate ratios, plated onto LB agar supplemented with the corresponding antibiotic, and incubated inverted at 37 °C for 16 h. Colony-forming units (CFUs) were subsequently enumerated on each plate to quantify bacterial (VNP-SNase) distribution and clearance.

### 2.18 Scanning electron microscopy (SEM)

Neutrophils to the desired confluence on sterile coverslips (Invitrogen Countess). Fix cells with 2.5% glutaraldehyde (Nanjing Chemical Material Corp, Nanjing, China) in 0.1 mol/L sodium cacodylate buffer (pH 7.4) (Thermo Fisher Scientific, MA, USA) for 2 h at room temperature or overnight at 4 °C. After fixation, rinse cells with PBS three times for 5 min each. Post-fix cells with 1% OsO_4_ (Sigma–Aldrich, MO, USA) in PBS for 1 h at 4 °C. Dehydrate cells through a graded series of ethanol or acetone (30%, 50%, 70%, 90%, 100%) for 10 min each. Replace 100% ethanol or acetone with a propylene oxide solution (Huntsman Corporation, TX, USA) for 10 min. Transfer samples to a critical point dryer and process them with liquid carbon dioxide to avoid ice crystal formation. Remove coverslips from culture dishes and mount them onto SEM stubs using double-sided adhesive tape. Sputter coat samples with a thin layer of gold or gold-palladium. Place stubs in the SEM chamber (JEOL, MA, USA) and adjust the pressure to the optimal level for imaging.

### 2.19 Transmission electron microscopy (TEM)

Neutrophils were fixed in a solution of 2.5% glutaraldehyde in 0.1 mol/L sodium cacodylate buffer for 2 h at 4 °C and washed three times with 0.1 mol/L sodium cacodylate buffer. Post-fixation was performed using 1% osmium tetroxide for 1 h at 4 °C. Dehydration of the cells was carried out using a graded ethanol series (50%, 70%, 90%, and 100%) for 15 min each at room temperature. Then, the cells were infiltrated with propylene oxide for 1 h. Cells were then infiltrated and embedded in freshly prepared embedding resin. To enhance contrast, the sections were stained with 2% uranyl acetate for 15 min, followed by a 5-min staining with lead citrate. The stained sections were mounted onto Formvar-coated copper grids and allowed to dry completely. Finally, the sections were examined using a transmission electron microscope to visualize the cellular ultrastructure.

### 2.20 Western blot

Cells were lysed on ice with Sangon lysis buffer (Shanghai, China) for 20 min. The lysate was centrifuged at 12,000 rpm for 15 min (TGL-16MS, BIORIDGE), and the supernatant was collected. Protein concentration was determined using a BCA protein assay kit (Beyotime, Shanghai, China). Proteins were denatured, separated by SDS-PAGE, and subsequently transferred to a PVDF membrane (Merck Millipore, USA). The membrane was incubated with primary antibodies after being blocked with 5% skim milk (DairyAmerica, USA), followed by incubation with secondary antibodies. Target proteins were detected using ECL reagent (Tanon, Shanghai, China). Secreted proteins in the bacterial culture medium were collected by TCA precipitation (Bioesn, Shanghai, China) and processed as described for Western blot analysis. Details of the antibodies are provided in Supporting Information Table S2.

### 2.21 Fluorescence activated cell sorting (FACS)

On Days 3 and 9 post-treatment, peripheral blood was collected in anticoagulant tubes (Shuangwei Biotechnology, Nanjing, China) containing heparin from the treated mice and processed into single-cell suspensions after lysing red blood cells. Tumor tissue was digested using RPMI 1640 medium (1 mg/mL collagenase I) at 37 °C, 200 rpm for 30 min, followed by red blood cell lysis and filtration of the cell suspension through a 200 μm filter. The single-cell suspensions were then incubated at 4 °C for 20 min with 0.5% bovine serum albumin (Sigma–Aldrich, MO, USA), followed by surface staining with the relevant antibodies for 30 min at 4 °C. After erythrocyte lysis and surface staining, pellet the cells (500×*g*, 5 min, 4 °C), discard the supernatant, resuspend the pellet in 200 μL freshly prepared Fix/Perm working solution (Thermo Fisher, USA), vortex briefly, incubate 30 min at 4 °C in the dark, wash twice with 200 μL 1 × permeabilization buffer (Thermo Fisher, USA) under the same centrifugation conditions, resuspend in 100 μL 1 × permeabilization buffer containing the appropriate fluorochrome-conjugated intracellular antibodies at 1 μL per sample, incubate 30 min at room temperature in the dark, wash twice again with 200 μL permeabilization buffer, and finally resuspend in 200 μL PBS for acquisition. (antibody details can be found in the Supporting Information Table S3). Stained cells were analyzed using a BD LSRFortessa flow cytometer, and the data were analyzed using FlowJo VX software.

### 2.22 Immunofluorescence, immunohistochemistry, and hematoxylin and eosin (H&E) staining

Neutrophils (1.0 × 10^6^) were plated in 1 mL of antibiotic-free RPMI 1640 medium in 24-well plates with coverslips coated with poly-l-lysine (Sigma–Aldrich, MO, USA), and after 6 h of static culture, VNP was added to the cells at a multiplicity of infection (MOI) of 100:1 and then co-cultured for 6 h, followed by treatment with gentamicin (50 μg/mL) for 1 h. After gently removing the medium, cells were fixed with 500 μL of fixative for 30 min and then stained with 1 μL Sytox Green (Yeasen, Shanghai, China) for 15 min. For frozen tissue sections, the tissues were fixed, washed, and stained as described above. Animal tissue sections were prepared by Servicebio Technology (Wuhan, China). The immunofluorescence staining for the markers CD8, PD-1, CD66b, PD-L1, CXCR4 and CD62L on animal tissues was performed by Dreambio Biotechnology (Nanjing, China). Antibody details can be found in Table S3.

### 2.23 β-Galactosidase staining

Following co-incubation of B16F10 with VNP or VNP combined with CGK733(5 μmol/L, MCE, USA) or VNP combined with Pifithrin-*α* (10 μmol/L, Selleck, USA) for 8 h, the culture medium was aspirated and the cells were washed once with PBS. Then, 1 mL of *β*-galactosidase staining fixative (Beyotime, Shanghai, China) was added and the cells were fixed at room temperature for 15 min. The staining solution was then removed, and 1 mL of staining working solution was added to each well. The cells were incubated at 37 °C overnight. On the subsequent day, the staining solution was removed, the tissue was washed with PBS and then observed and photographed using a microscope. For frozen tissue sections, thawing was the initial step, followed by a single wash with PBS and fixation in an appropriate volume of fixative at room temperature for 30 min. After fixation, the tissue was washed with PBS, and an appropriate amount of staining working solution was added, followed by overnight incubation at 37 °C. On the subsequent day, the staining solution was removed, the tissue was washed with PBS, and observed and photographed using a microscope (Leica, Wetzla, Germany).

### 2.24 Enzyme-linked immunosorbent assay (ELISA)

After bacterial treatment, the levels of proteins in mouse serum or tissue digestion fluid from each group were measured, including CXCL2, IL-1*β*, CCL-3, CCL-4, TNF-*α*, IFN-*γ*, GSF3R, C5AR1, MMP-9, and S100A8 using a mouse ELISA kit (Zeweil, Nanjing, China), and MPO-DNA using an ELISA kit (YuanjuBio, Shanghai, China). For the detection of CitH3-DNA and NE-DNA, 96-well plates were coated overnight at 4 °C with anti-histone H3 antibody (Abcam, UK) or anti-ELANE antibody (Huabio, Hangzhou, China), respectively. The plates were then blocked with 5% BSA for 2 h. Wells were washed four times with washing buffer, followed by the addition of 100 μL of sample and 50 μL of incubation buffer containing peroxidase-labeled anti-DNA antibody (Roche, Basel, Switzerland) for 2 h. Subsequently, the wells were washed three times with washing buffer, and 50 μL of peroxidase substrate was added to the wells for 30 min in the dark. Finally, 100 μL of ABTS peroxidase stop solution was added to the wells, and the absorbance was measured at 405 nm.

### 2.25 Hemolysis assay

The hemolytic potential of VNP-SNase was quantified using freshly isolated mouse erythrocytes. A 20% (*v*/*v*) erythrocyte stock was prepared in phosphate-buffered saline (PBS) and further diluted 1:20 to yield a working suspension. Aliquots of 100 μL of this suspension were transferred into a 96-well plate and mixed with an equal volume of either (i) PBS containing 5 × 10^5^ CFU of VNP-SNase, (ii) 1% (*v*/*v*) Triton X-100 (positive control for 100% lysis), or (iii) sterile 0.9% NaCl (negative control). After incubation at 37 °C for 2 h, the plates were centrifuged at 3000 rpm for 10 min. A 100 μL aliquot of the supernatant from each well was transferred to a fresh plate, and the absorbance at 415 nm was measured using a microplate reader (BioTek Instruments, VT, USA). Percent hemolysis was calculated as Eq. (3):

Hemolysis (%) = [(*A*_sample_–*A*_negative_)/(*A*_positive_–*A*_negative_)] × 100

### 2.26 Extraction of extracellular DNA from tumor tissues

On the nineth day after bacterial treatment, subcutaneous tumors were harvested from B16F10-bearing mice. The tumors were digested with 1 mg/mL collagenase IV (Yeasen, Shanghai, China) for 30 min, followed by centrifugation at 300×*g* for 10 min. The supernatant was collected and stored for further analysis. A portion of the supernatant was purified using a DNA extraction kit (Cat: DC102-01, Vazyme, Nanjing, China) to obtain high-purity DNA. The concentration of DNA was measured using a Nanodrop spectrophotometer (Thermo Scientific, USA) and then multiplied by the dilution factor to calculate the total amount of extracellular DNA in the tumor tissue. The purified DNA was further analyzed by agarose gel electrophoresis to assess the degree of DNA fragmentation. Another portion of the supernatant was used for ELISA to determine the levels of the NETs marker MPO-DNA (YuanjuBio, Shanghai, China) in the extracellular fraction of the tumor tissue.

### 2.27 Assessment of antigen uptake and presentation by DC cells

DC2.4 cells in the logarithmic growth phase were seeded into 12-well plates at a density of 5 × 10^5^ cells per well. The cells were stimulated with 1 μg/mL LPS (Beyotime, S1732, Shanghai, China) for 6 h to activate the DC2.4 cells. After removing the LPS, the plates were divided into four groups: control group; SNase group (5 μg); DNA group (100 μg); and the DNA + SNase group. The cells were then cultured for 24 h in a cell incubator at 37 °C with 5% CO_2_. Subsequently, mRNA was extracted from the cells, and the transcriptional levels of genes related to antigen uptake and presentation were analyzed using RT-PCR. Genomic DNA from B16F10 cells was purified using a DNA extraction kit (Cat: DC102-01, Vazyme, Nanjing, China).

### 2.28 Data analysis

ImageJ software was used to quantify immunofluorescence area, fluorescence intensity, protein band density, and wound healing width. Data were analyzed using GraphPad Prism software v.9. The *t*-test was used for comparisons between two groups, and one-way ANOVA was used for more than two groups. Statistical significance was defined as a probability value of 0.05 or less; ns indicates not significant (*P* > 0.05). Survival analysis was conducted using Kaplan–Meier survival curves, and differences between different patient or mouse groups were assessed using log-rank statistics. Data are presented as the mean ± standard deviation (SD).

## 3 Results

### 3.1 VNP induces B16F10 and HUVECs senescence to inhibit tumor growth and angiogenesis

**Figure 1:**
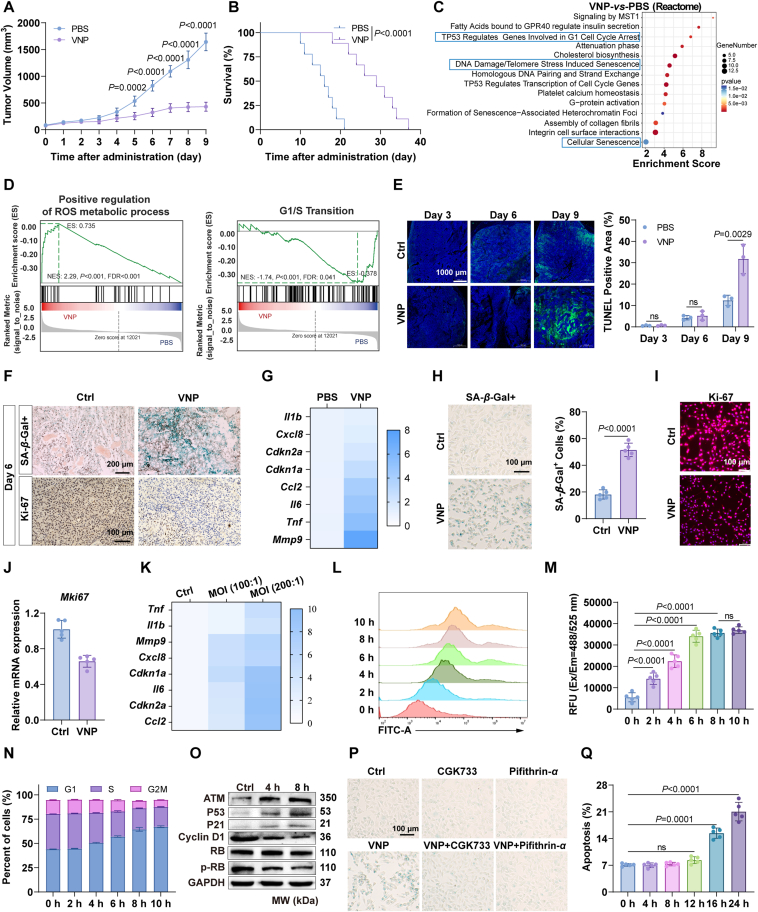
B16F10 senescence induced by VNP. (A) Tumor growth curve. (B) Overall survival curve. (C) Reactome enrichment. (D) GSEA graphs of G1/S cell cycle arrest (left) and ROS (right). (E) TUNEL staining. Scale bar = 1000 μm. (F) *β*-galactosidase staining (upper) and Ki-67 immunohistochemistry staining (lower) of tumor tissues. Scale bar = 200 μm (upper) and 100 μm (lower). (G) Relative mRNA expression of SASP genes within tumor. (H) *β*-Galactosidase staining of B16F10 cells with VNP stimulation for 8 h. Scale bar = 100 μm. (I) Ki-67 immunofluorescence staining of B16F10 cells co-cultured with VNP for 8 h. Scale bar = 100 μm. (J) Relative mRNA expression of Ki-67 in B16F10 cells with VNP stimulation. (K) Relative mRNA expression of SASP genes in B16F10 cells. (L, M) ROS level changes in B16F10 cells measured by FACS (L) and microplate reader (M). (N) Cell cycle composition of B16F10 cells. (O) Western blotting to detect the protein expression levels of senescence-associated signaling pathways. (P) *β*-Galactosidase staining to assess the effects of VNP combined with ATM inhibitor (CGK733) or P53 inhibitor (Pifithrin-*α*) on senescence in B16F10 cells. Scale bar = 100 μm. (Q) Apoptosis in B16F10 cells co-cultured with VNP for 24 h. Data represent the mean ± SD in (A) (*n* = 7), (B) (*n* = 9), (C, D, H, J, M and Q) (*n* = 5) and (E and O) (*n* = 3). Statistical significance was determined using unpaired Student’s *t*-test in (H), (J). One-way ANOVA with Tukey test was used in (M, Q). Two-way ANOVA with Tukey test was used in (A, E). Log rank (Mantel–Cox) tests in (B).

We first evaluated the anti-tumor activity of VNP in a melanoma mouse model and discovered that VNP significantly suppressed tumor growth and prolonged survival (Fig. 1A and B, Supporting Information Fig. S1A and S1B). To elucidate the underlying mechanism, we performed transcriptome sequencing on VNP-treated B16F10 cells. As depicted in Fig. 1C and D and Fig. S1C–S1G, the results suggested that VNP might induce cellular senescence, evidenced by elevated ROS levels, DNA damage, cell cycle arrest, and reduced cell proliferation. The tumor growth curve revealed that VNP exhibited significant tumor growth inhibition by day six. However, H&E and TUNEL staining showed no significant differences in tumor necrosis compared to the control group at day six, further implying that VNP induces cellular senescence before causing tumor cell death (Fig. 1E, Fig. S1H). Consistent with our hypothesis, *β*-galactosidase (SA-*β*-gal) and Ki67 staining of tumor tissue confirmed that VNP induced significant cellular senescence and proliferation slowdown by Day 6 (Fig. 1F, Fig. S1I). Senescence-associated SA-*β*-gal is the acidic (pH 6.0) active form of the lysosomal enzyme GLB1. Its accumulation is a hallmark of senescence, driven by a 2- to 3-fold increase in lysosomal mass and hyperactivation in senescent cells, resulting in 5- to 10-fold higher SA-*β*-gal activity compared to young cells^24^. Moreover, VNP enhanced the expression of SASP-related genes within the tumor tissue, including *Tnf*, *Il1b*, *Mmp9*, *Cxcl8*, *Cdkn1a*, *Cdkn2a*, *Il6*, and *Ccl2* (Fig. 1G). Cell-level experiments yielded similar conclusions. Unexpectedly, VNP treatment for 8 h significantly increased the percentage of SA-*β*-gal positive B16F10 cells (Fig. 1H) while decreasing Ki-67 expression (Fig. 1I and J, Fig. S1J), accompanied by significant upregulation of SASP genes (Fig. 1K). Furthermore, VNP progressively increased ROS levels in B16F10 cells in a time-dependent manner (Fig. 1L and M), induced G1/S phase cell cycle arrest (Fig. 1N), increased ATM, P53, and P21 expression, and decreased Cyclin D and phosphorylated RB levels (Fig. 1O, Fig. S1K). Importantly, pharmacological inhibition using the ATM-specific inhibitor CGK733 or the P53 inhibitor Pifithrin-*α* significantly attenuated VNP-induced cellular senescence (Fig. 1P, Fig. S1L), providing direct evidence for the functional involvement of these signaling molecules. Notably, VNP did not significantly induce B16F10 apoptosis within 12 h (Fig. 1Q, Fig. S1M), suggesting that it triggers cellular senescence prior to tumor cell death. These findings suggest that VNP may induce B16F10 senescence *via* the DNA damage–ATM–P53–P21–Cyclin D1 signaling axis.

**Figure 2:**
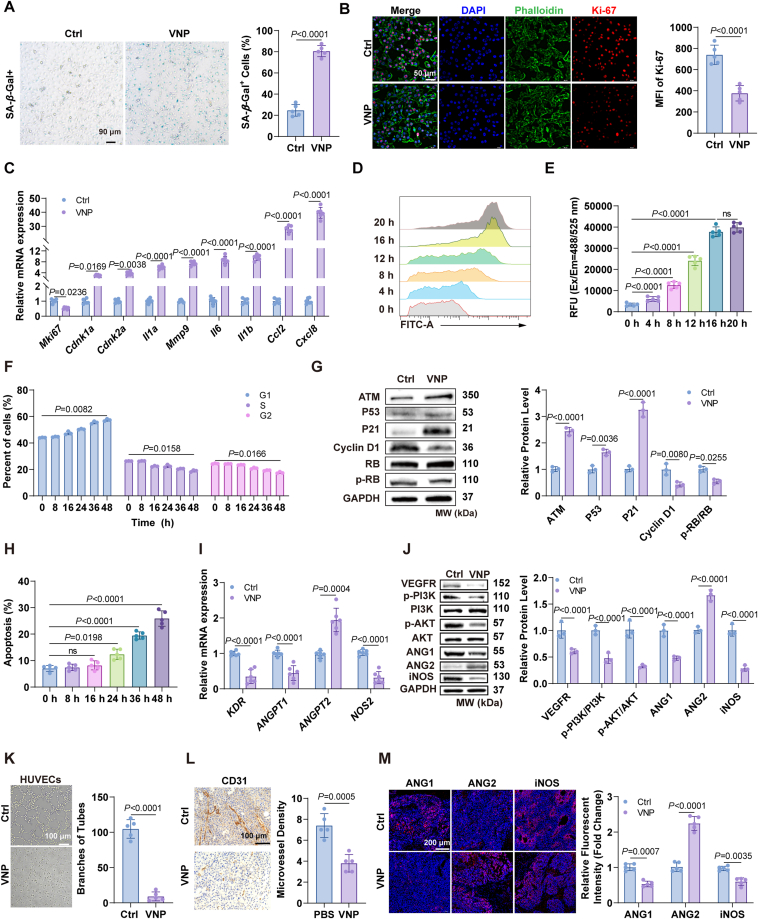
VNP induces HUVECs senescence and inhibits angiogenesis. (A) *β*-Galactosidase staining of HUVECs co-cultured with VNP for 12 h. Scale bar = 90 μm. (B) Ki-67 immunofluorescence staining of HUVECs co-cultured with VNP for 12 h. Scale bar = 50 μm. (C) Relative mRNA expression of SASP genes in HUVECs. (D, E) ROS levels in HUVECs after co-culture with VNP, measured by FACS (D) and microplate reader (E). (F) Cell cycle composition of HUVECs. (G) Western blotting to detect the protein expression levels of senescence-associated signaling pathways. (H) HUVECs apoptosis at different time points of co-culture with VNP. (I) Relative mRNA expression of angiogenesis-related genes in HUVECs stimulated by VNP. (J) Western blotting analysis of proteins expression involving the PI3K–AKT signaling pathway in HUVECs co-cultured with VNP. (K) The effect of VNP on the tube formation assay of HUVECs. Scale bar = 100 μm. (L) CD31 immunohistochemical staining of tumor tissue. Scale bar = 100 μm. (M) Immunofluorescence staining of angiogenesis-related genes within tumor tissues. Scale bar = 200 μm. Data represent the mean ± SD. All data are representative of two independent experiments in (A–C, E, F, H, I, K–M) (*n* = 5) and (G, J) (*n* = 3). Statistical significance was determined using unpaired Student’s *t*-test in (A, B, K, L). One-way ANOVA with Tukey test was used in (E, H). Two-way ANOVA with Tukey test was used in (C, F, G, I, J, M).

Similar results were observed in HUVECs, where VNP induced senescence (Fig. 2A–H). Considering the critical role of the PI3K–AKT pathway in angiogenesis^25^, ^26^, ^27^, ^28^, ^29^, we examined the effect of VNP on the expression levels of key PI3K–AKT pathway proteins and angiogenesis-related proteins in HUVECs. VNP downregulated angiogenesis-related gene expression through the PI3K–AKT signaling pathway (Fig. 2I and J) and inhibited HUVECs tube formation (Fig. 2K). Immunohistochemistry and immunofluorescence staining of tumor tissue showed that VNP significantly reduced tumor angiogenesis (Fig. 2L and M). In conclusion, VNP induced senescence in both tumor cells and vascular endothelial cells to inhibit tumor progression.

### 3.2 VNP induces neutrophil recruitment and senescence in TME, leading to NETs formation

**Figure 3:**
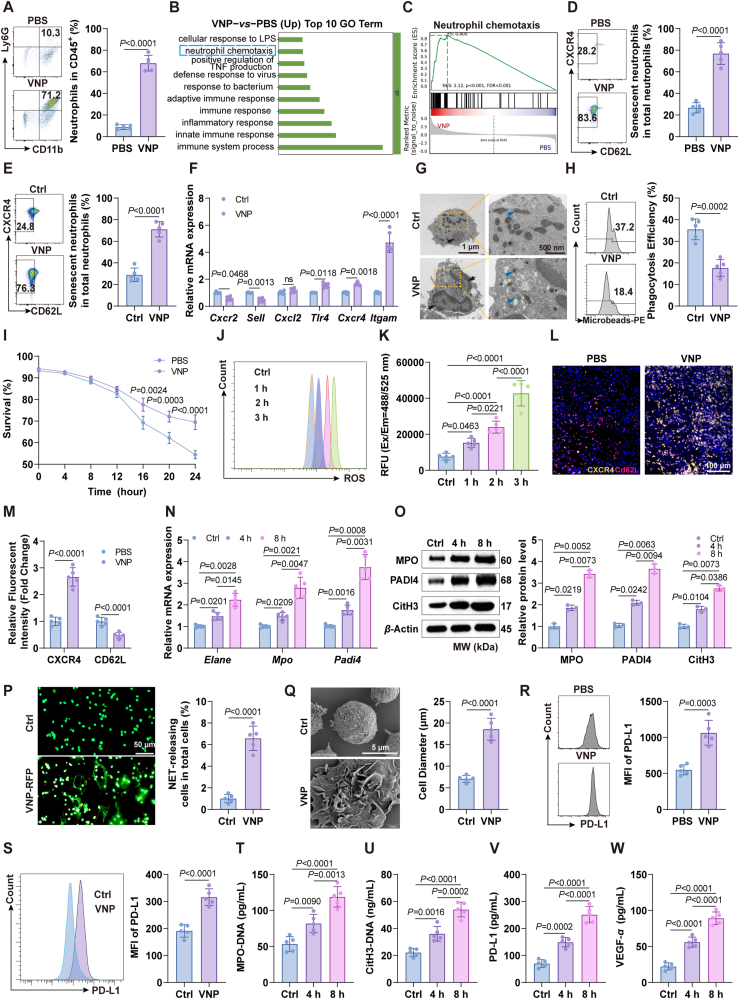
VNP upregulates neutrophil PD-L1 expression and induces neutrophil senescence to form NETs. (A) FACS analysis of neutrophil proportion within tumors after VNP administration. (B) Biological Process analysis of transcriptome sequencing data from VNP-treated tumor tissues. (C) GSEA of Neutrophil Chemotaxis Pathways. (D) FACS analysis of neutrophil senescence within tumors after VNP administration. (E) FACS analysis of neutrophil senescence with VNP stimulation *in vitro*. (F) Relative mRNA expressions of neutrophil senescence related genes. (G) TEM was used to observe the morphology of neutrophils. Black arrows indicate nuclear, blue arrows indicate mitochondria. Scale bar = 1 μm (left) and 500 nm (right). (H) FACS examined the phagocytosis of neutrophils. (I) The lifespan of neutrophils isolated from PBS and VNP tumor. (J, K) The level of ROS in neutrophils, detected by FACS (J) and microplate (K). (L, M) Immunofluorescence detection of neutrophil senescence in tumor tissues following VNP treatment in mice. Scale bar = 100 μm. (N) Relative mRNA expressions of NETs markers in neutrophils stimulated with VNP. (O) Western blotting to detect the protein expression levels of NETs markers. (P) VNP-RFP induces NETs formation, with DNA fluorescence staining (Sytox Green) analysis. Scale bar = 50 μm. (Q) SEM was used to observe the morphology of neutrophils. Scale bar = 5 μm. (R, S) FACS analysis of PD-L1 expression on neutrophils *in vivo* (R) and *in vitro* (S). (T–W) ELISA quantification of MPO-DNA, CitH3-DNA, PD-L1 and VEGF-*α* in the supernatant. Data represent the mean ± SD in (A, D–F, H, I, K, M, N, P–W) (*n* = 5) and (B, C, O) (*n* = 3). Statistical significance was determined using unpaired Student’s *t*-test in (A, D, E, H, P–S). One-way ANOVA with Tukey test was used in (K, T–W). Two-way ANOVA with Tukey’s *post hoc* test was used in (F, I, M, N, O).

Based on previous studies of VNP and our current findings, VNP treatment significantly remodels the tumor immune microenvironment (TIM)^1^^,^^4^^,^^30^^,^^31^, primarily characterized by robust neutrophil recruitment. Specifically, the proportion of neutrophils among total immune cells increased from 10.3% to 71.2% (Fig. 3A). Furthermore, RNA sequencing of tumor tissues revealed significant activation of neutrophil chemotaxis signaling pathways in VNP-treated tumors (Fig. 3B and C), along with marked upregulation of key neutrophil-associated chemokines (Supporting Information Fig. S2). These observations prompted us to investigate whether VNP could induce neutrophil senescence and how this might influence the therapeutic efficacy of VNP. Our results demonstrated that the percentage of senescent neutrophils (defined as CXCR4^hi^, CD62L^low^) in the VNP-treated group was approximately three times higher than in the control group (Fig. 3D). To further validate these findings, we assessed the effect of VNP on neutrophil senescence using bone marrow-derived neutrophils at the cellular level. Consistently, VNP treatment led to a significant increase in the proportion of senescent neutrophils (Fig. 3E). Next, we examined the expression of senescence-associated markers in neutrophils. As expected, levels of *Tlr4*, *Cxcr4*, and *Itgam* were significantly upregulated, while the negative markers *Cxcr2* and *Sell* were downregulated following VNP treatment (Fig. 3F). We extended our analysis to other immune cell types, including T cells (Jurkat), macrophages (Raw264.7), and dendritic cells (DCs, DC2.4), and VNP did not induce senescence in any of these cell types (Supporting Information Figs. S3–S5). Transmission electron microscopy (TEM) revealed that VNP treatment induces characteristic senescent morphological changes in neutrophils. Compared to the control group, VNP-treated neutrophils exhibited larger and more irregular nuclei with multiple lobes, accompanied by a reduction in mitochondrial number and shortening of mitochondrial length—features that are well-established hallmarks of cellular senescence^32^ (Fig. 3G). Furthermore, VNP-treated neutrophils demonstrated diminished phagocytic capabilities (Fig. 3H). Additionally, neutrophils isolated from tumors in the VNP group showed a shortened lifespan compared to controls (Fig. 3I). Consistent with the senescence process, ROS levels gradually increased during co-incubation with VNP (Fig. 3J and K, Supporting Information Fig. S6). Moreover, the immunofluorescence results clearly demonstrate that VNP treatment led to significant upregulation of CXCR4 accompanied by marked downregulation of CD62L (Fig. 3L and M), providing compelling *in vivo* evidence that VNP promotes cellular senescence in tumor tissues. These results collectively demonstrate that VNP induces neutrophil senescence both *in vitro* and *in vivo*.

Previous studies have shown that senescent neutrophils are more likely to form NETs^16^^,^^33^^,^^34^. We performed experiments using etoposide, a well-established inducer of cellular senescence^35^^,^^36^, to treat neutrophils. The results clearly showed that pharmacologically-induced neutrophil senescence also leads to robust NETs formation (Supporting Information Fig. S7), establishing a direct causal link between neutrophil senescence and NETs generation. Therefore, we examined the levels of NETs in neutrophils after VNP treatment. The levels of NETs markers ELANE, MPO, and PADI4 were significantly increased in neutrophils co-cultured with VNP in a time-dependent manner (Fig. 3N and O). Immunofluorescence and scanning electron microscopy (SEM) results revealed that neutrophils underwent vigorous NETosis upon encountering VNP-RFP. The cellular membranes of neutrophils were observed to protrude into a reticular structure following VNP treatment (Fig. 3P and Q). These results demonstrated that VNP contributed to NETs formation. Considering that NETs formation is accompanied by the release of PD-L1 and VEGF-*α*^37^^,^^38^, and Analysis of the Human Protein Atlas (HPA) database revealed neutrophils as the primary immune cell type expressing PD-L1^39^, ^40^, ^41^, ^42^, ^43^, ^44^, ^45^, ^46^ (Supporting Information Fig. S8), we found through FACS and RT-PCR that VNP significantly upregulated PD-L1 and VEGF-*α* levels in neutrophils both *in vivo* and *in vitro* (Fig. 3R and S, Supporting Information Fig. S9), which might contribute to the up-regulation of PD-L1 and VEGF-*α* levels in TME after NETs formation. Also supporting these conclusions, the expression of NETs markers MPO-DNA and CitH3-DNA, as well as PD-L1 and VEGF-*α*, were significantly increased in the supernatant of VNP-treated neutrophils in a time-dependent manner (Fig. 3T–W). Therefore, VNP leads to neutrophil senescence to promote NETs formation, accompanied by an upregulation of PD-L1 and VEGF-*α*.

### 3.3 VNP-induced NETs promote angiogenesis and T-cell exhaustion in TME

**Figure 4:**
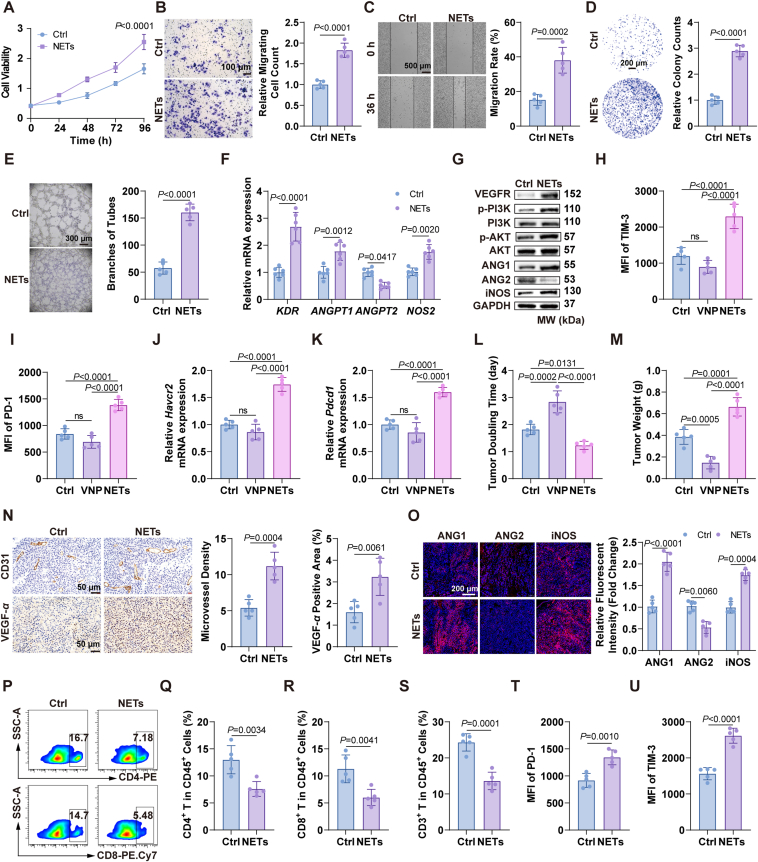
NETs promote angiogenesis *via* the PI3K–AKT signaling pathway and T cell exhaustion. (A–E) NETs enhance the proliferation (A), migration (Scale bar = 100 μm) (B), wound healing (Scale bar = 500 μm) (C), colony formation (Scale bar = 200 μm) (D), and tube formation (Scale bar = 300 μm) (E) of HUVECs. (F) Relative mRNA expressions of angiogenesis-related genes in HUVECs stimulated with NETs. (G) Western blotting to detect the protein expression levels of angiogenesis-related genes in HUVECs after treatment with NETs. (H, I) MFI of CD8^+^ T exhaustion marker TIM-3 and PD-1. (J, K) Relative mRNA expressions of exhaustion in CD8^+^ stimulated with NETs. (L) TDT. (M) Tumor weight. (N) Immunohistochemistry staining of CD31^+^ cells and VEGF-α. Scale bar = 50 μm. (O) The expression of indicated proteins in angiogenesis was detected by immunofluorescence within tumors. Scale bar = 200 μm. (P–S) The proportion of CD4^+^, CD8^+^ and CD3^+^T cells within tumors. (T, U) The MFI changes of CD8^+^ T cells exhaustion marker. Data represent the mean ± SD in (A–F, H–K, L–U) (*n* = 5) and (G) (*n* = 3). Statistical significance was determined using unpaired Student’s *t*-test in (B–E, N, Q–U). One-way ANOVA with Tukey test was used in (H–M). Two-way ANOVA with Tukey test was used in (A, F, O).

Researchers have reported that neutrophils can promote tumor progression *via* releasing NETs^47^, ^48^, ^49^, ^50^. Analysis of The Cancer Genome Atlas (TCGA) database revealed that high expression of NETs markers, including *Padi4*, *Ctsc*, and *Mpo*, is associated with poor prognosis in melanoma (Supporting Information Fig. S10). Consequently, we investigated the effects of NETs on tumor progression. Our findings demonstrate that NETs promote B16F10 cell proliferation, migration and invasion (Supporting Information Fig. S11A–S11K). Moreover, NETs also promote HUVECs proliferation, migration, and tube formation (Fig. 4A–E, Supporting Information Fig. S12A). RT-PCR and Western blot results indicated that NETs elevated the expression of pro-angiogenic genes in HUVECs, such as VEGFR, Angiopoietin-1 (ANG1), iNOS, pPI3K, and pAKT, while significantly downregulating Angiopoietin-2 (ANG2) expression (Fig. 4F and G, Fig. S12B), suggesting that NETs promote angiogenesis by activating the VEGFR-PI3K-AKT signaling pathway.

Concerning NETs led to the release of PD-L1, we explored the potential effects of NETs on tumor immunity. Upon stimulation with NETs, CD8^+^ T cells exhibited a significant upregulation of the exhaustion markers PD-1 and TIM-3. Interestingly, VNP alone did not directly induce the upregulation of these markers (Fig. 4H–K, Fig. S12C), indicating that the T cell dysfunction observed was predominantly mediated by NETs rather than VNP itself. Similarly, activated CD8^+^ T cells co-cultured with blood neutrophils exhibited a consistent protein expression profile (Fig. S12D–S12I). These results demonstrated that both neutrophils and NETs can induce T cell exhaustion, thereby inhibiting tumor immunity.

*In vivo* experiments showed that NETs significantly promoted the melanoma tumor growth, whereas VNP markedly inhibited tumor growth (Fig. 4L and M, Fig. S12J). Immunohistochemical staining and immunofluorescence analysis of tumor tissues revealed that NETs significantly upregulated the expression of CD31, VEGF-*α*, ANG1, and iNOS, while downregulating ANG2 expression (Fig. 4N and O). FACS assessment of tumor-infiltrating T cells demonstrated that NETs significantly reduced the proportion of CD4^+^ and CD8^+^ T cells (Fig. 4P–S). Concurrently, CD8^+^ T cells exhibited a trend towards exhaustion and dysfunction (Fig. 4T and U), suggesting that NETs exert an unfavorable effect on the TIM. In conclusion, our findings demonstrate that NETs accelerate melanoma growth by promoting tumor growth, enhancing angiogenesis, and deteriorating the TIM.

### 3.4 Neutrophil depletion strengthens VNP anti-tumor capacity

**Figure 5:**
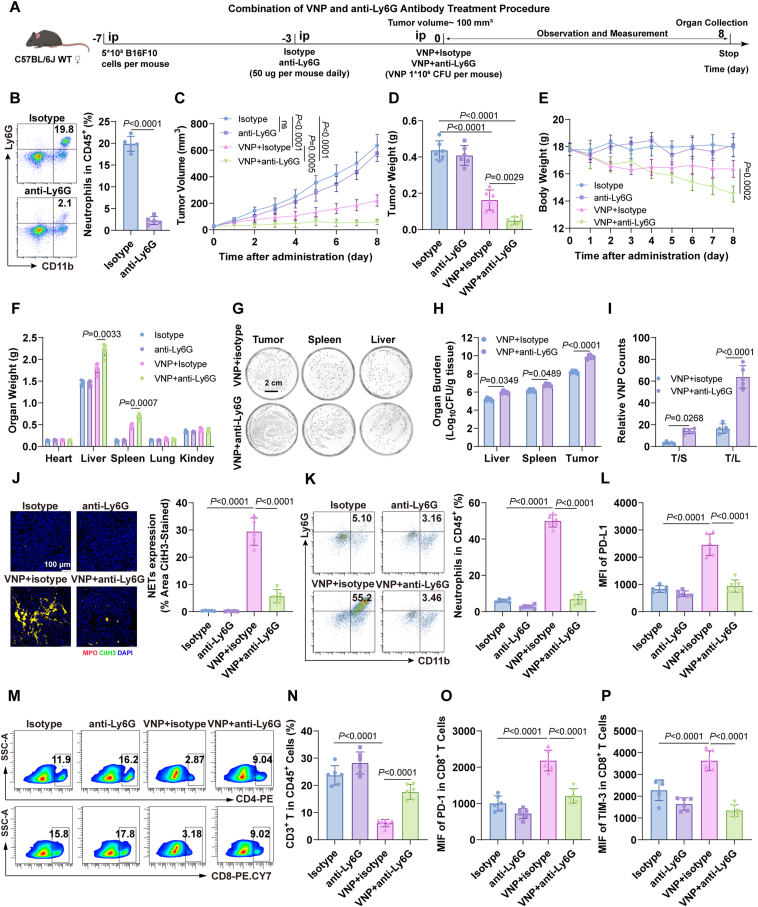
VNP combined with neutrophil depletion antibody enhances anti-tumor activity. (A) Schematic diagram of the animal experiment for VNP combined with neutrophil depletion antibody. (B) FACS to detect the depletion effect of neutrophils in mouse blood. (C) Tumor growth curves. (D) Tumor weight. (E) Daily weight changes. (F) Organ weight. (G) Tumor, liver, and spleen homogenates from VNP and VNP combined with antibody 8 days after administration, appropriately diluted and plated on LB agar, photographed and counted after cultured 16 h. Scale bar = 2 cm. (H, I) Organ load of tumor, liver, and spleen (absolute numbers of VNP) 8 days after administration. Based on the number of VNP CFU on the plate on the 8th day (H), calculate the ratio of tumor to liver and tumor to liver (I). (J) Immunofluorescence images of NETs. Scale bar = 100 μm. (K, L) FACS to detect the percentage of neutrophils (K) in immune cells (CD45^+^) and MFI of PD-L1^+^ in neutrophils (L). (M) FACS analysis showing the ratio of CD4^+^ T cells and CD8^+^ T cells. (N) Frequency of CD3^+^ T cells among CD45^+^ cells. (O, P) MFI analysis of CD8^+^ T cell exhaustion markers (PD-1, TIM-3). Data represent the mean ± SD in (B, J–N, N–P) (*n* = 5), (C–F) (*n* = 6) and (H, I) (*n* = 3). Statistical significance was determined using unpaired Student’s *t*-test in (B). One-way ANOVA with Tukey test was used in (D, J–L, N–P). Two-way ANOVA with Tukey’s *post hoc* test was used in (C, E, F, H, I).

Although VNP inhibits tumor growth and angiogenesis by inducing senescence in tumor cells and vascular endothelial cells, its recruitment of neutrophils and induction of their senescence promotes the formation of NETs in the TME, which significantly drive tumor progression. Therefore, in theory, clearing NETs from the TME could mitigate tumor progression caused by neutrophil senescence without interfering with VNP’s effects on tumor cells and vascular endothelial cells. Based on this, we investigated the therapeutic potential of combining anti-Ly6G antibody treatment with VNP therapy, as shown in Fig. 5A. The anti-Ly6G antibody effectively depleted approximately 90% of neutrophils from the blood (Fig. 5B). Compared to VNP monotherapy, the combination therapy demonstrated superior anti-tumor efficacy, as evidenced by reduced tumor growth (Fig. 5C), prolonged TDT (Supporting Information Fig. S13A), and decreased tumor weight (Fig. 5D, Fig. S13B). However, mice receiving combination therapy showed significant body weight loss and hepatosplenomegaly (Fig. 5E and F, Fig. S13C and S13D), indicating potential risks associated with systemic neutrophil depletion. The combination therapy significantly enhanced both intra-tumoral VNP titers and tumor targeting efficiency (Fig. 5G–I), potentially contributing to its improved therapeutic outcomes. Furthermore, NETosis was markedly reduced in the combination group (Fig. 5J). FACS revealed a significant decrease in PD-L1-positive neutrophils in both blood (Fig. S13E and S13F) and tumor tissue (Fig. 5K and L) of the combination group compared to VNP monotherapy. This reduction corresponded with increased proportions of CD4^+^ and CD8^+^ T cells in the tumor tissue (Fig. 5M and N, Fig. S13G and S13H). Beyond altering T cell frequencies, the combination therapy also improved T cell functionality. While T cells in the VNP group exhibited an exhausted phenotype characterized by elevated PD-1 and TIM-3 expression, this exhaustion was reversed in the combination therapy group (Fig. 5O and P). These findings demonstrate that combining VNP therapy with anti-Ly6G antibody treatment enhances therapeutic efficacy through multiple mechanisms: reducing neutrophil infiltration, suppressing NETs formation within the TME, and reinvigorating anti-tumor immune responses.

### 3.5 VNP-SNase demonstrates enhanced tumor suppression by degrading NETs

Due to the side effects caused by the direct inhibition of neutrophils, we used two methods to inhibit the level of NETs in the TME instead of directly inhibiting neutrophils, in hopes of achieving stronger anti-tumor effects and lower toxic side effects. These are DNase I, which can degrade the DNA backbone of NETs^51^^,^^52^, and GSK484, an inhibitor that targets the key enzyme PADI4 in the formation of NETs^53^. The results showed that both DNase I and GSK484, when used in combination with VNP, exhibited excellent anti-tumor effects (Supporting Information Fig. S14A–S14D, S14H–S14K). Meanwhile, compared with the direct inhibition of neutrophils, both significantly improved weight loss and hepatosplenic toxicity in mice (Fig. S14E–S14G, S14L and S14M), indicating that NETs inhibition is effectively in enhancing the therapeutic effect of VNP with favorable biosafety.

**Figure 6:**
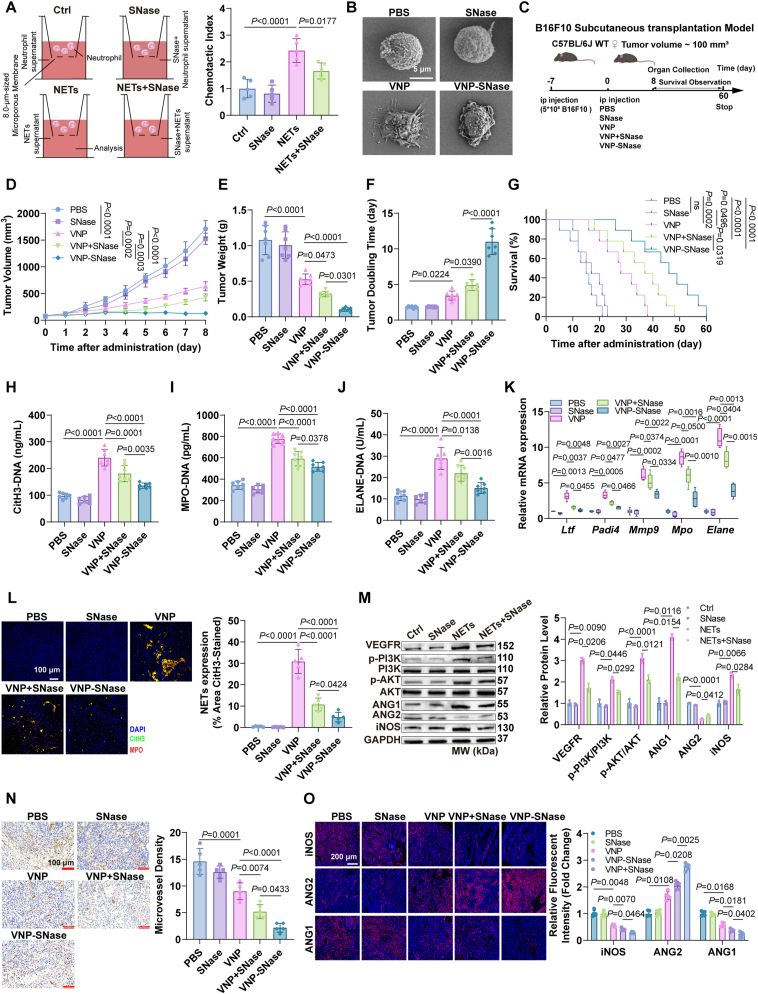
VNP-SNase enhances anti-tumor effects by degrading NETs. (A) Neutrophil chemotaxis assay. (B) SEM was used to observe the morphology of neutrophils and NETs. Scale bar = 5 μm. (C) B16F10 tumor-bearing mice C57BL/6J are given VNP, VNP-SNase (5.0 × 10^5^ CFU/mouse), SNase (100 μg/mouse) *via* intraperitoneal injection. (D) Tumor growth curve. (E) Tumor weight. (F) TDT. (G) Overall survival curve. (H–J) ELISA quantification of CitH3-DNA, MPO-DNA and NE-DNA in serum. (K) Relative mRNA expressions of NETs-related markers genes in tumors. (L) Immunofluorescence images of NETs. Scale bar = 100 μm. (M) Western blot was conducted to determine the protein expression of angiogenesis-related genes in HUVECs. (N) Immunohistochemistry staining of CD31^+^ expression within tumors. Scale bar = 100 μm. (O) The expression of indicated proteins in angiogenesis was detected by immunofluorescence within tumors. Scale bar = 200 μm. Data represent the mean ± SD in (A, K, L, N, O) (*n* = 5), (D–F, H–J) (*n* = 7), (G) (*n* = 9) and (M) (*n* = 3). Statistical significance was determined using one-way ANOVA with Tukey test in (A, E, F, H–J, L, N). Two-way ANOVA with Tukey’s *post hoc* test was used in (D, K, M, O). Log rank (Mantel–Cox) tests in (G).

However, these methods possess a frequent drug administration and a low patient compliance. Therefore, we constructed an engineered VNP-based bacterial strain, VNP-SNase, which secretes SNase within the TME to degrade NETs (Supporting Information Fig. S15A). The growth characteristics, tumor invasiveness, bacterial colony formation and surface morphology of VNP-SNase are consistent with those of VNP (Fig. S15B–S15G). Moreover, hemolysis assays demonstrated that both VNP-SNase and VNP exhibited hemolysis rates of less than 5%, confirming their safety *in vitro* (Fig. S15H). We confirmed the extracellular secretion of SNase using Western Blots (Fig. S15I). The ability of the purified SNase (Fig. S15J–S15M) to inhibit NETs chemotaxis (Fig. 6A) and degrade nucleic acids as well as its enzyme activity was successfully verified *in vitro* (Fig. S15N–S15P). In addition, SEM and immunofluorescence results showed that VNP-SNase significantly disrupted the morphology of NETs (Fig. 6B, Fig. S15Q). As a DNA-degrading enzyme, SNase may also degrade DNA from other sources within the TME, such as DNA released by necrotic tumor cells or extracellular DNA derived from immune cells, thereby influencing immune responses. To address these concerns, we extracted and purified extracellular DNA from tumor tissues treated with either VNP or VNP-SNase. Quantitative analysis showed that compared to VNP, VNP-SNase slightly reduced the total extracellular DNA level per unit tumor mass (Supporting Information Fig. S16A) and moderately enhanced DNA fragmentation (Fig. S16B). Notably, detection of the NETs-specific marker MPO-DNA demonstrated that VNP-SNase significantly decreased extracellular MPO-DNA levels in tumor tissues (Fig. S16C), providing strong evidence for the specific NETs-degrading effect of VNP-SNase. To further validate the impact of VNP-SNase on immune responses, we evaluated the effect of SNase on antigen presentation using the DC2.4 cell line. *In vitro* experiments revealed that B16F10 cell DNA significantly upregulated the expression of antigen presentation-related genes in DCs, while SNase treatment showed no significant effect on the expression levels of these genes (Supporting Information Fig. S17), indicating that SNase does not interfere with DC antigen presentation function. This result is highly consistent with the conclusion that VNP-SNase specifically degrades NETs.

We next examined the function of SNase *in vitro* by co-culturing B16F10 with SNase, NETs, or a combination of SNase and NETs. Results showed that SNase mitigated NETs-induced promotion of proliferation, migration and invasion in B16F10 cells (Supporting Information Fig. S18A–S18K). In a melanoma mouse model, SNase alone had no significant effect on tumor progression, while both VNP-SNase and VNP combined with exogenous SNase significantly inhibited tumor growth and prolonged survival compared to wild-type VNP, with the engineered VNP-SNase possessing superior therapeutic performance (Fig. 6C–G, Supporting Information Fig. S19A and S19B). Concurrently, VNP-SNase significantly ameliorated weight loss and hepatosplenomegaly compared to VNP and VNP combined with SNase (Fig. S19C–S18E). Upon analyzing tumor tissues from different mouse groups, we found that VNP-SNase exhibited enhanced tumor colonization and targeting compared to VNP and VNP combined with exogenous SNase (Fig. S19F–S19H). Furthermore, NETs-related markers in mouse serum, including CitH3-DNA, MPO-DNA, and NE-DNA, were significantly reduced (Fig. 6H–J). RT-PCR and immunofluorescence analyses revealed that in tumor tissues, VNP-SNase significantly reduced NETs formation compared to the VNP or VNP combined with SNase groups. This reduction was evidenced by the downregulation of NETs markers, including *Padi4*, *Elane*, *Mpo*, *Mmp9*, and *Ltf*, as well as decreased NETs signals (Fig. 6K and L). To further highlight VNP-SNase’s therapeutic advantage, comparative animal studies revealed that VNP-SNase outperformed VNP plus DNase I treatment, showing greater reduction in TME NETs levels and stronger tumor growth inhibition (Supporting Information Fig. S20). Considering the pro-angiogenic effects of NETs, we evaluated VNP-SNase’s impact on tumor angiogenesis. Results reveal that SNase weakened NETs-induced angiogenesis in HUVECs through the VEGFR–PI3K–AKT signaling axis (Fig. 6M). Immunohistochemical and immunofluorescence assays of tumor tissues further confirmed that VNP-SNase demonstrated a more potent inhibition of angiogenesis compared to VNP or VNP combined with SNase groups (Fig. 6N and O).

In biosafety experiments, no significant pathological damage to organs was observed in any group (Supporting Information Fig. S21A). Minor elevations in hepatic and renal function biochemical indicators were noted, but no abnormalities were detected in other groups (Fig. S21B). Blood indicator examinations revealed only minor changes in the VNP and VNP combined SNase group, including decreased lymphocytes and platelets, and slightly increased neutrophils and monocytes (Fig. S21C). Furthermore, our comprehensive biodistribution analyses demonstrate VNP-SNase’s excellent targeting specificity, with significantly higher tumor accumulation *versus* normal tissues (Fig. S19F–S19H). Kinetic studies show rapid blood clearance (peaking at 4 h and nearly eliminated by 24 h; Supporting Information Fig. S22) coupled with prolonged tumor-selective retention compared to normal organs (Supporting Information Fig. S23). These findings collectively validate VNP-SNase’s favorable therapeutic window, combining rapid systemic clearance with sustained intratumoral presence and minimal off-target distribution. These results demonstrate that VNP-SNase enhances VNP-mediated tumor growth inhibition and angiogenesis blockade by degrading NETs, while exhibiting superior biosafety.

**Figure 7:**
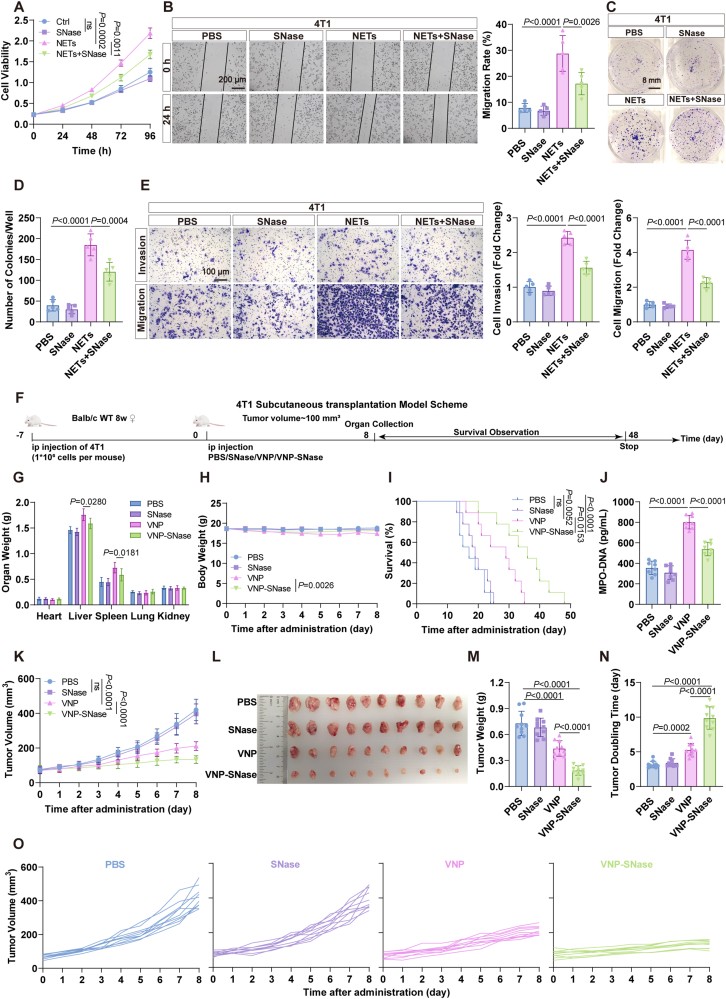
VNP-SNase inhibited the 4T1 proliferation, migration and tumor growth caused by NETs. (A) 4T1 proliferation assay. (B) Wound healing assay. Scale bar = 200 μm. (C, D) Colony formation assay. Scale bar = 8 mm. (E) *Trans*-well migration and invasion assay. Scale bar = 100 μm. (F) Schematic diagram of VNP-SNase antitumor efficacy against 4T1 tumor-bearing mice. (G) Organ weight. (H) Body weight. (I) Survival curve. (J) Changes of the NETs marker MPO-DNA in serum. (K) Tumor growth curve. (L, M) Photographs of tumor (L) and tumor weight (M). (N) TDT. (O) Tumor growth curves for each mouse in (K). Data represent the mean ± SD. All data are representative of two independent experiments. in (A, B, D, E) (*n* = 5) and (I) (*n* = 9), (G, H, J–N) (*n* = 10). Statistical significance was determined using One-way ANOVA with Tukey test in (B, D, E, J, M, N). Two-way ANOVA with Tukey’s post hoc test was used in (A, G, H, K). Log rank (Mantel–Cox) tests in (I).

Apart from the melanoma model, subcutaneous mouse models of breast cancer and liver cancer were also employed to assess the anticancer efficacy of VNP-SNase. *In vitro* cellular experiments revealed that the combination of NETs and SNase could significantly impede the proliferation, migration, and invasion of 4T1 and Hepa1-6 cells (Fig. 7A–H, Supporting Information Fig. S24A–S24H). Similar results were also obtained in their corresponding model mice (Fig. 7I–O, Fig. S24I–S24N).

### 3.6 VNP-SNase reshapes the TIM to enhance antitumor immunity

**Figure 8:**
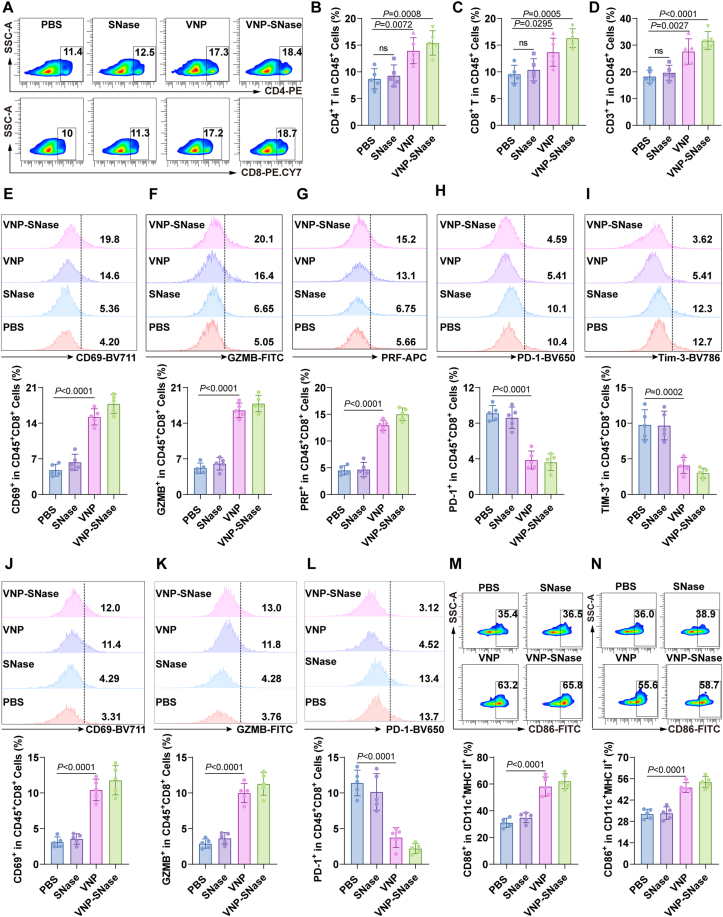
VNP therapy potentiates anti-tumor immunity at early stage (Day 3). (A–D) Representative FACS plots and quantification of CD3^+^, CD4^+^, and CD8^+^ T-cell frequencies among CD45^+^ immune cells. (E) The expression level of CD69 in tumor-infiltrating CD8^+^ T-cells was detected by FACS. (F, G) Frequency of GZMB^+^ and PRF^+^ functional CD8^+^ T cells in tumors. (H, I) Frequency of exhaustion markers (PD-1^+^ and TIM-3^+^) on CD8^+^ T cells in tumors. (J) The expression level of CD69 in Tumor-draining lymph nodes (TDLNs) CD8^+^ T-cells was detected by FACS. (K, L) Frequency of GZMB^+^ functional and PD-1^+^ exhaustion markers on CD8^+^ T cells in TDLNs. (M, N) The expression levels of CD86^+^ DCs in tumor (M) and in TDLNs (N) were measured by FACS. Data represent the mean ± SD. All data are representative of two independent experiments. in (B–N) (*n* = 5). Statistical significance was determined using one-way ANOVA with Tukey test in (B–N).

Considering the potential modulatory effects of NETs on the TME, we employed FACS to analyze immune cell alterations in the TME 3 days after bacterial therapy. Compared to the PBS control, VNP treatment significantly increased PD-L1-high expressing neutrophils and neutrophils senescence (Supporting Information Fig. S25). Additionally, VNP treatment significantly augmented the proportion of T cells, particularly CD4^+^ and CD8^+^ T cells (Fig. 8A–D). Furthermore, we observed enhanced activation of CD8^+^ T cells, as evidenced by the increased expression of the activation marker CD69 (Fig. 8E). This was accompanied by the upregulation of cytotoxic molecules in GZMB^+^ CD8^+^ and PRF^+^ CD8^+^ T cells (Fig. 8F and G), and concurrent downregulation of exhaustion markers in PD-1^+^ CD8^+^ and TIM-3^+^ CD8^+^ T cells (Fig. 8H and I). CD4^+^ T cells exhibited consistent changes with those observed in CD8^+^ T cells (Supporting Information Fig. S26). Parallel alterations were also observed in tumor-draining lymph nodes (TdLNs) (Fig. 8J–L), suggesting systemic immune activation. DCs, as key antigen-presenting cells, are instrumental in recruiting and stimulating antitumor T cells^2^^,^^4^^,^^54^^,^^55^. Accordingly, we evaluated the changes in DCs following treatment. VNP significantly induced the maturation of dendritic cells both in tumors and TdLNs (Fig. 8M and N). The initiation of antigen-specific CD8^+^ T cell responses is critically contingent upon the assistance of mature DCs. These findings are congruent with previous research indicating that VNP therapy enhances tumor immunity in the early stages (Day 3)^1^^,^^4^.

**Figure 9:**
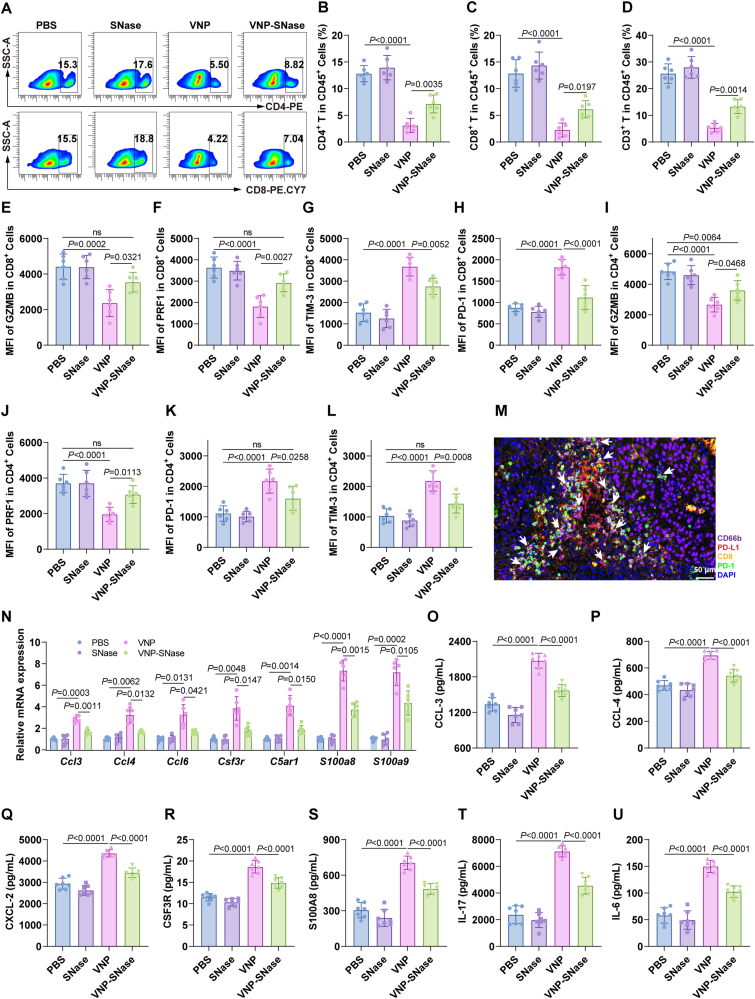
VNP-SNase treatment reshapes the TME (Day 9). (A–D) FACS analysis showing the proportion of CD4^+,^ CD8^+^, CD3^+^ T cells in immune cells. (E–H) MFI of CD8^+^ T cell cytotoxic markers GAMB, PRF (E, F) and exhaustion markers Tim-3, PD-1 (G, H). (I–L) MFI of CD4^+^ T cell exhaustion markers GAMB, PRF (I, J) and cytotoxic markers PD-1, TIM-3 (K, L). (M) Immunofluorescence staining of TANs and CD8^+^ T cells. Representative cells are indicated by arrows, including PD-L1^+^ Neu (red), CD66b^+^ Neu (purple) cells, PD1^+^ T cells (green), CD8^+^ T cells (golden yellow). Scale bar = 50 μm. (N) Relative mRNA expression of neutrophil chemotaxis-related genes within tumors. (O–U) ELISA quantification of neutrophil chemotaxis-related genes in serum. Data represent the mean ± SD in (B–L) (*n* = 6), (N) (*n* = 5) and (O–U) (*n* = 7). Statistical significance was determined using One-way ANOVA with Tukey test in (B–L, O–U). Two-way ANOVA with Tukey’s *post hoc* test was used in (N).

Extending our observation to Day 9, both VNP and VNP-SNase groups maintained significantly higher TANs levels compared to PBS. Compared to the VNP group, the proportion of PD-L1^+^ neutrophils was decreased in the VNP-SNase group. Consistently, senescent neutrophils increased in the VNP group, with VNP-SNase mitigating this phenotype (Supporting Information Fig. S27). Blood neutrophil changes corroborated these findings (Supporting Information Fig. S28). Previous studies have shown that NETs promote hepatocyte and endothelial cell senescence^56^^,^^57^. Therefore, we hypothesized that VNP-SNase might inhibit neutrophil senescence by reducing NETs levels in the TME. Flow cytometry results revealed that NETs induce neutrophil senescence (CXCR4^high^, CD62L^low^), while SNase reverses this effect (Supporting Information Fig. S29A). RT-PCR experiments further confirmed this finding: NETs significantly upregulated senescence-associated positive markers and downregulated negative markers in neutrophils, whereas SNase treatment counteracted these changes (Fig. S29B–S29I). Thus, VNP-SNase alleviates neutrophil senescence in the TME by degrading NETs. In contrast to day 3 data, VNP induced a notable decrease in CD4^+^ and CD8^+^ T cell numbers by Day 9 (Fig. 9A–D), accompanied by increased expression of CD8^+^ T cell exhaustion markers PD-1 and TIM-3, and decreased GZMB and PRF (Fig. 9E–H). VNP-SNase reversed these effects (Fig. 9I–L). Immunofluorescence colocalization revealed physical proximity between PD-L1^+^ neutrophils and PD1^+^ CD8^+^ T cells (Fig. 9M), suggesting direct interaction. These results collectively indicate that VNP-induced PD-L1^+^ TANs inhibit CD8^+^ T cell cytotoxic function in the melanoma TME on Day 9, while VNP-SNase ameliorates this phenomenon. To confirm that VNP-SNase’s effect was mediated by weakened neutrophil chemotaxis following NETs degradation by SNase, we assessed neutrophil chemotaxis-related gene expression in tumor tissue. As shown in Fig. 9N, VNP increased expression of *Ccl3*, *Ccl4*, *Ccl6*, *Csf3r*, *C5ar1*, *S100a8*, and *S100a9*, which was reversed by VNP-SNase. Corroborating these findings, serum tests revealed significantly reduced protein levels of CCL-4, CCL-4, CXCL2, CSF3R, S100A8, IL-17 and IL-6 in the VNP-SNase group (Fig. 9O–U).

### 3.7 VNP-SNase exhibits a potent anti-metastatic effect in melanoma metastasis models

**Figure 10:**
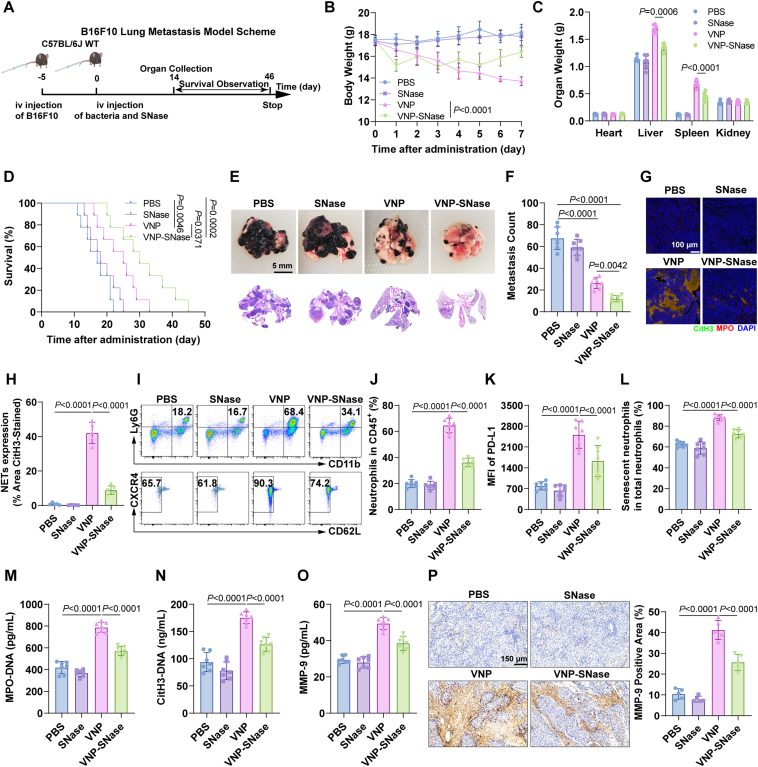
Enhanced therapeutic efficacy of VNP-SNase against melanoma lung metastasis. (A) Experimental protocol for the treatment of the B16F10 lung metastasis model with VNP-SNase. (B) Daily weight changes. (C) Organ weight. (D) Overall survival rate. (E) Representative photographs of lung metastatic tumors (upper), and H&E scanning (lower). Scale bar = 5 mm. (F) Quantification of B16F10 lung metastasis foci. (G, H) Immunofluorescence images of NETs. Scale bar = 100 μm. (I–L) FACS analysis showing the proportion of neutrophils among immune cells (upper left, J) and the proportion of senescent neutrophils among total neutrophils (lower left, L) and MFI of PD-L1 (K). (M–O) ELISA quantification of NETs markers MPO-DNA (M), CitH3-DNA (N), and MMP-9 (O) in serum. (P) Immunohistochemistry staining of MMP-9 expression within tumors. Scale bar = 150 μm. Data represent the mean ± SD in (A–C, F, J–O) (*n* = 7) and (H, P) (*n* = 5), (D) (*n* = 9). Statistical significance was determined using one-way ANOVA with Tukey test in (F, H, J–P). Two-way ANOVA with Tukey’s *post hoc* test was used in (B, C). Log rank (Mantel–Cox) tests in (D).

Tumor metastasis is a critical stage in cancer progression and the primary cause of treatment failure^58^, ^59^, ^60^. Recent studies have implicated NETs in cancer metastasis^61^. We therefore investigated the effect of VNP-SNase on B16F10 lung metastasis (Fig. 10A). Compared to VNP alone, VNP-SNase demonstrated a more potent therapeutic effect, as evidenced by increased body weight (Fig. 10B), reduced hepatosplenomegaly (Fig. 10C, Supporting Information Fig. S30A and S30B), decreased lung weight (Fig. S30C), and prolonged survival time (Fig. 10D). Both lung metastatic foci counts and H&E staining results indicated that while SNase monotherapy had no significant effect on melanoma lung metastasis, VNP and VNP-SNase showed marked anti-metastatic effects, with VNP-SNase being more efficacious (Fig. 10E and F, Fig. S30D). Immunofluorescence results revealed that VNP-SNase significantly reduced intra-tumoral NETs (Fig. 10G and H) and the proportion of PD-L1^+^ neutrophils, while also delaying neutrophil senescence (Fig. 10I–L). Furthermore, serum levels of NETs markers, including MPO-DNA and CitH3-DNA, were significantly lower in the VNP-SNase group compared to the VNP group (Fig. 10M and N). Given that MMP-9, a component of NETs^62^^,^^63^, is associated with tumor metastasis, we examined MMP-9 levels in serum and lung tissues. Results showed a significant decrease in MMP-9 levels in the VNP-SNase group (Fig. 10O and P). Biosafety experiments corroborated previous findings: liver and kidney function indicators remained within normal ranges (Fig. S30E and S30F). Apart from a decrease in lymphocytes and platelets, and a slight increase in neutrophils and monocytes in the VNP group, no abnormalities were observed in other groups (Fig. S30G).

## 4 Discussions

The safety and therapeutic efficacy of engineered bacteria for cancer treatment are two critical issues in the development of tumor bacterial therapy^1^^,^^64^. The unsatisfactory single-agent therapeutic results of VNP in clinical trials underscore the urgent need to further elucidate its antitumor mechanism. Cellular senescence has been discovered in multiple studies to participate in tumor progression^12^^,^^14^^,^^22^, functioning as a potential antitumor therapeutic target that can influence tumor cell apoptosis, angiogenesis, and modulate the TIM remarkably similar to those observed with VNP in cancer treatment. This suggests that cellular senescence may also be involved in VNP’s antitumor mechanism. Currently, the potential triggers of cellular senescence include endogenous mechanisms such as replicative senescence, epigenetic alterations, and metabolic disturbances, as well as exogenous factors like DNA damage, oxidative stress, and mechanical stress^65^, ^66^, ^67^. In this study, we employed differential transcriptomics to reveal that VNP significantly induced ROS elevation, DNA damage, cell cycle arrest, and decreased cell proliferation, indicating VNP’s capacity to promote tumor cell senescence. RT-PCR and Western blotting results demonstrated that VNP concentration-dependently upregulated the expression of ATM, P53, and P21 while downregulating Cyclin D1 protein levels and RB phosphorylation in B16F10 cells. Importantly, pharmacological inhibition using the ATM-specific inhibitor CGK733 or the P53 inhibitor Pifithrin-*α* significantly attenuated VNP-induced cellular senescence, providing direct evidence for the functional involvement of these signaling molecules. These findings suggest that VNP may induce senescence *via* the DNA damage–ATM–P53–P21–Cyclin D1 signaling axis. Additionally, we observed that VNP significantly elevated intracellular ROS levels, implying that ROS-mediated DNA damage might contribute to senescence. Further animal-level experiments and pathological results confirmed that VNP first induced tumor cell senescence before causing tumor cell death. Similar findings were observed in HUVECs. Therefore, this research elucidates a novel mechanism by which VNP exerts its antitumor effects: inhibiting tumor growth and angiogenesis through promoting senescence in both tumor cells and vascular endothelial cells. This conclusion expands our understanding of VNP’s anti-tumor mechanisms, elucidating the role of cellular senescence in VNP-based therapy. However, given this study’s primary focus on neutrophils and the role of NETs formation in VNP’s antitumor effects, we did not fully validate or confirm the mechanistic details of VNP-induced senescence—such as how VNP elevates ROS or whether alternative DNA damage pathways are involved. These questions will be addressed in future work.

Complex interactions between bacteria and the host immune system often result in suboptimal intra-tumoral colonization of VNP^30^. Neutrophils, key immune defenders, play a crucial role in combating bacterial infections, and their heterogeneity contributes to their complex role in tumor development^17^^,^^68^, ^69^, ^70^. As expected, we observed a significant recruitment of neutrophils in the TME following VNP treatment. Consistent with these findings, pre-clinical and clinical studies employing other oncolytic bacteria—such as attenuated *Listeria monocytogenes* and *Escherichia coli* Nissle 1917 have similarly reported marked neutrophil enrichment in the TME^71^, ^72^, ^73^, ^74^, ^75^. However, the precise mechanisms by which neutrophils influence tumor progression, particularly their impact on VNP’s therapeutic effects, remain unclear. Neutrophils engulf and kill bacteria by releasing NETs, which can be induced by senescent neutrophils^16^^,^^17^. Considering VNP’s senescence-promoting effects in tumor cells and vascular endothelial cells, we investigated VNP’s impact on neutrophil senescence. *In vitro* experiments revealed that VNP induced neutrophil senescence and NETs production. NETs have been implicated in tumor initiation, progression, metastasis, and poor prognosis^76^. When we cultured B16F10 cells with NETs-containing medium, we found that NETs promoted cell proliferation and migration. Moreover, NETs can accelerate angiogenesis by promoting HUVECs proliferation through the VEGFR–PI3K–AKT pathway.

Studies in various malignancies have revealed that TANs frequently exhibit higher PD-L1 expression than other immune populations (*e*.*g*., macrophages or dendritic cells), positioning them as potentially dominant contributors to PD-1/PD-L1-mediated immunosuppression in certain contexts^40^, ^41^, ^42^, ^43^, ^44^, ^45^, ^46^. We also found that VNP upregulates PD-L1 expression in TANs and enhances NETs formation, consequently increasing PD-L1 levels in the TME. This mechanism induces cytotoxic T cell exhaustion and suppresses antitumor immunity. These findings indicate that VNP-induced neutrophil senescence leads to NETs formation which induces tumor progression and antitumor immunity suppression. In addition to neutrophils, the impact of VNP on a broader range of immune cells was examined, encompassing T cells (Jurkat), macrophages (Raw264.7), and dendritic cells (DC2.4). The results demonstrate that VNP fails to elicit a senescence response in these cell types. While the precise mechanism of VNP-triggered senescence remains to be elucidated, we fully recognize its importance and plan to investigate this in future studies, which may reveal additional therapeutic targets for VNP-based combinations.

Given the protumourigenic and immune-exhausting properties of NETs, we combined VNP with anti-neutrophil antibodies to treat tumor-bearing mice. This combination therapy significantly reduced intra-tumoral NETs by eliminating neutrophils, alleviating T cell exhaustion, and ultimately enhancing the anti-tumor effect of VNP. However, the combination group displayed more severe weight loss and hepatosplenomegaly, indicating that simply using neutrophil antibodies to systemically suppress neutrophils to enhance VNP treatment efficacy can lead to toxicity in other organs, resulting in significant adverse effects. Therefore, targeting the NETs formed by senescent neutrophils rather than neutrophils themselves may be a more viable approach. To this end, during VNP treatment, we introduced NETs inhibitors GSK484 or DNase I to degrade the DNA backbone of NETs^48^^,^^77^. Both solutions not only enhanced the therapeutic effect of VNP but also mitigated side effects in other organs, particularly alleviating hepatosplenomegaly significantly. In conclusion, combining VNP treatment with the inhibition or degradation of NETs presents an effective strategy for cancer therapy. This approach addresses the challenges posed by NETs in bacterial cancer therapy while minimizing systemic toxicity, offering a promising direction for future research and clinical applications.

However, the combined external supplementation inevitably increased the frequency of administration, diminishing the advantage of a single VNP administration and potentially reducing patient compliance. To address this issue, we engineered a VNP strain capable of expressing and secreting SNase, a type of *Staphylococcus aureus* nuclease with the ability to degrade nucleic acids^78^. Compared to DNase I (31 kDa), SNase (17 kDa) has a lower molecular weight and reduced immunogenicity^79^. Furthermore, DNase I contains disulfide bonds that require proper post-translational modification—a process potentially compromised when expressed by VNP, whereas SNase’s simpler structure (lacking disulfide bonds) makes it more suitable for VNP expression. Moreover, both cellular and animal experiments confirmed SNase’s effective NETs clearance when delivered by VNP. Recent studies have demonstrated that *Staphylococcus aureus* can evade NETs-mediated killing due to the nucleic acid degradation capabilities of SNase^78^^,^^80^^,^^81^, supporting the feasibility of our engineered VNP approach. Compared with NETs-targeting small-molecule PADI4 inhibitors or nanoparticle-based neutrophil-targeting systems, VNP-SNase confers three decisive advantages: 1) Tumor selectivity: Exploiting VNP’s innate tropism, SNase is enriched almost exclusively in the TME, sparing normal tissues and avoiding the systemic exposure inherent to small-molecule inhibitors; 2) Self-amplifying therapy: After a single i.p. dose, VNP colonizes hypoxic tumor niches and continuously secretes SNase, generating durable NETs suppression without the need for repeated dosing required by small molecules or nanoparticles; 3) Dynamic microenvironment modulation—The living vectors adapt to temporal changes in the TME, whereas static small-molecule inhibitors or nanoparticles cannot respond to evolving tumor biology.

The tumor-targeting capability of VNP employed in this study relies on the hypoxic TME, making VNP-SNase particularly effective against solid tumors capable of forming hypoxic TME (*e*.*g*., melanoma, hepatocellular carcinoma, and breast cancer), as demonstrated by multiple experiments in this study. Conversely, for hematologic malignancies or neurogenic tumors that typically lack such hypoxic microenvironments, VNP’s targeting efficiency may be compromised, potentially limiting its therapeutic efficacy. Therefore, in addition to the melanoma model, we further validated the anti-tumor efficacy of VNP-SNase in both hepatocellular carcinoma and breast cancer models. Compared to standard VNP, VNP-SNase more potently suppressed the progression of multiple cancer types and melanoma lung metastasis, while also extending the survival time of model mice by disrupting the NETs induced by VNP treatment. Additionally, in our B16F10 passive lung transfer experiments, the observed >10% single-day weight loss likely stems from intravenous administration, which enables rapid bacterial accumulation in these organs, triggering acute toxicity. Weight loss is indeed a common phenomenon in live bacterial therapies^1^^,^^54^^,^^55^^,^^82^^,^^83^. Our comparative studies in both tumor-bearing and healthy mice demonstrated that intraperitoneal injection significantly ameliorates these adverse effects, reducing both weight loss and hepatosplenomegaly compared to intravenous delivery (Supporting Information Figs. S31 and S32). While these findings confirm that VNP and its derivatives retain some toxicity risks, they also suggest clinically actionable mitigation strategies through route optimization—favoring intraperitoneal or even oral administration in clinical settings to minimize potential adverse effects while maintaining therapeutic efficacy.

When assessing the effects of VNP-SNase on the immune landscape within the TME, our findings revealed an initial increase in the proportion of cytotoxic T cells in the early stages of treatment. However, the aforementioned results did not align with our expectations, leading us to hypothesize that the proportion of neutrophils might be insufficient to exert an influence on T cells. We extended our investigation to the ninth day post-bacterial therapy, and it was encouraging to find that VNP-SNase effectively mitigated T-cell exhaustion, a phenomenon attributed to the excessive presence of NETs that had been exacerbated by prior VNP treatment.

By revealing this multifaceted impact of VNP on various cell types within the TME, we gain deeper insights into its therapeutic potential and mode of action. This new perspective on VNP-induced senescence across different cell populations opens up novel avenues for optimizing bacterial cancer therapy and potentially developing combination strategies that leverage these senescence-inducing properties.

## 5 Conclusions

In conclusion, VNP inhibits tumor growth and angiogenesis by inducing cellular senescence in tumor cells and vascular endothelial cells. Simultaneously, it recruits neutrophils in the TME and promotes NETs formation through senescence. However, these NETs can potentially counteract VNP therapeutic effects by promoting tumor growth, metastasis, angiogenesis, and T cell exhaustion. The engineered VNP strain VNP-SNase overcomes this limitation by degrading NETs in the TME, blocking NETs-mediated effects, and thereby enhancing the antitumor efficacy of VNP.

## Author contributions

Zichun Hua and Xiao Chen supervised and conceived the project. Zichun Hua, Xiao Chen and Wanfa Dong designed the experiments. Wanfa Dong performed the experiments, analyzed the data, and wrote manuscript. Zichun Hua and Xiao Chen modified the manuscript. Chenyang Li helped analyze the transcriptome sequencing. Xiao Chen and Jiqiang Lu helped with the preparation of the figures. Chenyang Li, Jiqiang Lu, Lin Weng, Yicong Xu, Min Xu, Yanhui Wu, Peiqi Li, Zixuan Shan, Pengyou Shang, Liangliang Dai, Tao Zhang, Yanlong Jia, Tianyun Wang, Wenjie Ren and Ping Lu helped with the experiments using animals. Xiao Chen and Wanfa Dong contributed to the discussion, and Zichun Hua provided relevant advice. All authors discussed the results and reviewed the manuscript.

## Conflicts of interest

The authors declare no competing interests.

## Appendix A Supporting information

The following is the Supporting Information to this article:
