# Supplementary material for: Engineered bacteria reprogram tumor microenvironment via cell senescence and neutrophil extracellular traps degradation
Source: Acta Pharm Sin B. 2026 Jan 28;16(4):2332–56. doi: 10.1016/j.apsb.2026.01.030 (PMC13104682; doi:10.1016/j.apsb.2026.01.030)
Supplement: Multimedia component 1 [file mmc1.pdf]

Supporting Information for

Original article

Engineered bacteria reprogram tumor microenvironment *via* cell senescence and neutrophil extracellular traps degradation

Wanfa Dong[a], Chenyang Li[b], Jiqiang Lu[a], Lin Weng[a], Yicong Xu[b], Min Xu[a], Peiqi Li[a], Yanhui Wu[a], Zixuan Shan[a], Pengyou Shang[a], Liangliang Dai[a], Tao Zhang[c], Yanlong Jia[c], Tianyun Wang[c], Wenjie Ren[c], Ping Lu[c], Xiao Chen[a,*], Zichun Hua[a,b,c,d,*]

[a]*School of Biopharmacy, China Pharmaceutical University, Nanjing 211198, China*

[b]*The State Key Laboratory of Pharmaceutical Biotechnology, College of Life Sciences, Nanjing University, Nanjing 210023, China*

[c]*Faculty of Pharmaceutical Sciences, Xinxiang Medical University, Xinxiang 453003, China*

[d]*Changzhou High-Tech Research Institute of Nanjing University and Jiangsu TargetPharma Laboratories Inc., Changzhou 213164, China*

Received 17 July 2025; received in revised form 26 August 2025; accepted 16 September 2025

*Corresponding authors.

E-mail addresses: xchen@cpu.edu.cn (Xiao Chen), 1020192596@cpu.edu.cn (Zichun Hua).

Supporting Figures S1–S32

Supporting Tables S1–S3

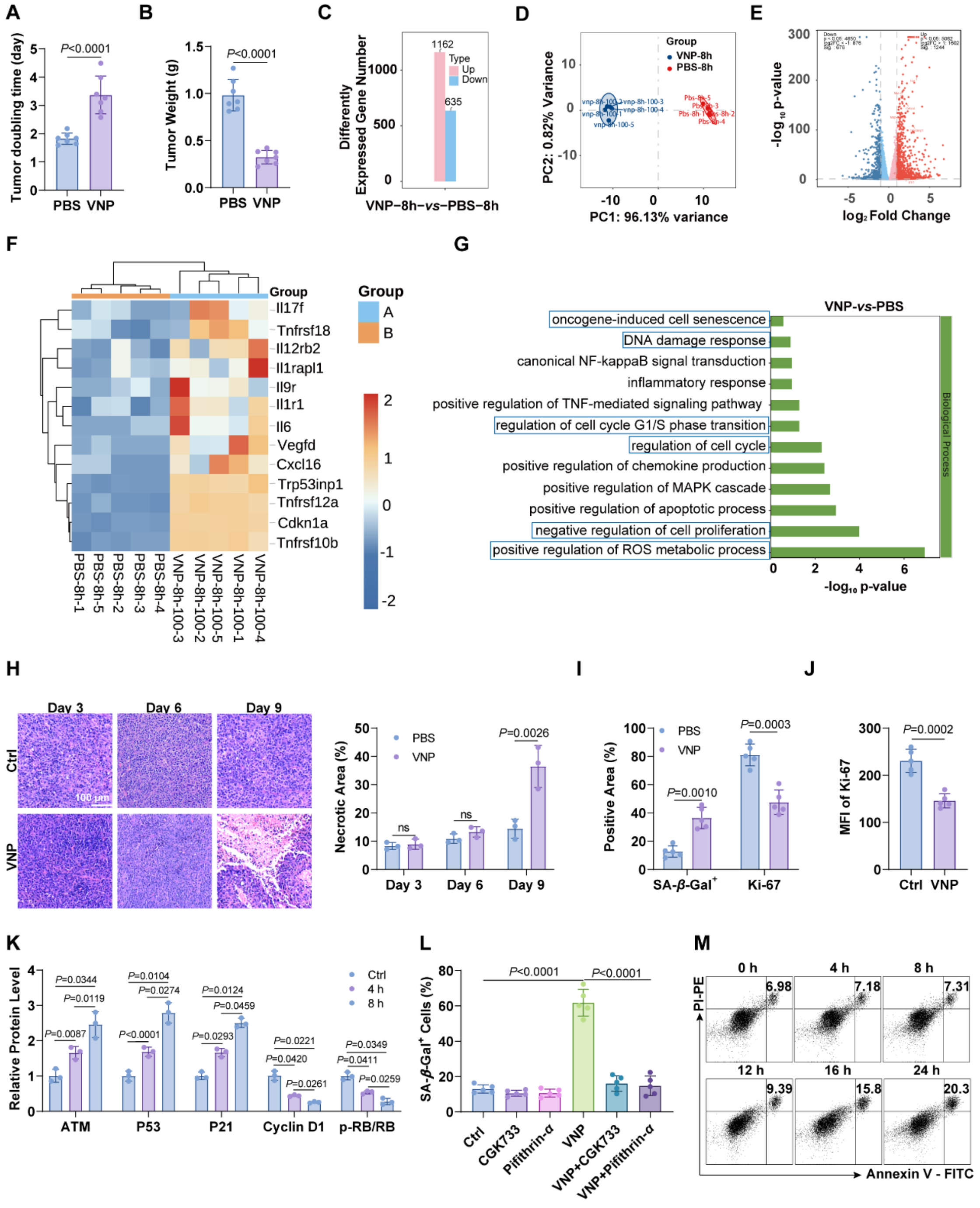

Figure S1 VNP induces B16F10 cell senescence. (A) Tumor doubling time. (B) Tumor weight. (C) Bar chart analysis of differential gene expression. (D) Principal component analysis (PCA). (E) Volcano plots. (F) Heatmap of representative genes associated with cellular senescence. (G) Biological process of GO enrichment. (H) H&E staining. Scale bar = 100 μm. (I) Statistical analysis for the SA-*β*-Gal and Ki-67 immunohistochemistry staining within tumor tissues. (J) Ki-67 immunofluorescence staining of B16F10 cells with VNP stimulation. (K)

Statistical analysis of Western blot detected senescence-associated gene expression in B16F10 cells with VNP stimulation. (L) Statistical analysis of SA-β-Gal staining detected the effects of VNP combined with ATM inhibitor (CGK733) or P53 inhibitor (Pifithrin-α) on B16F10 cell senescence. (M) B16F10 apoptosis with VNP stimulation. Data represent the mean ± SD. All data are representative of two independent experiments. In (A, B) (n = 7), (C–G, I, J, L) (n = 5) and (H, K) (n = 3). Statistical significance was determined using unpaired Student's t-test in (A, B, J). One-way ANOVA with Tukey test was used in (L). Two-way ANOVA with Tukey test was used in (H, I, K).

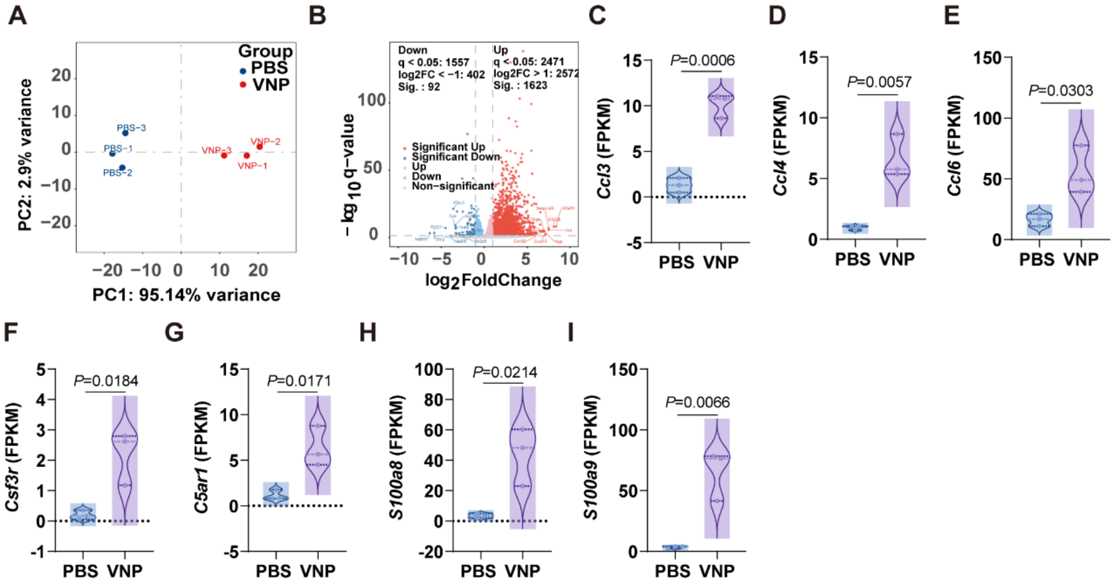

Figure S2 Transcriptomic sequencing analysis of B16F10 melanoma tissues following VNP treatment. (A) Principal component analysis (PCA). (B) Volcano plots. (C–I) Differential expression analysis of neutrophil chemotaxis-related genes in melanoma tissues following VNP treatment. Data represent the mean ± SD in (A–I) (n = 3). Statistical significance was determined using unpaired Student's t-test in (C–I).

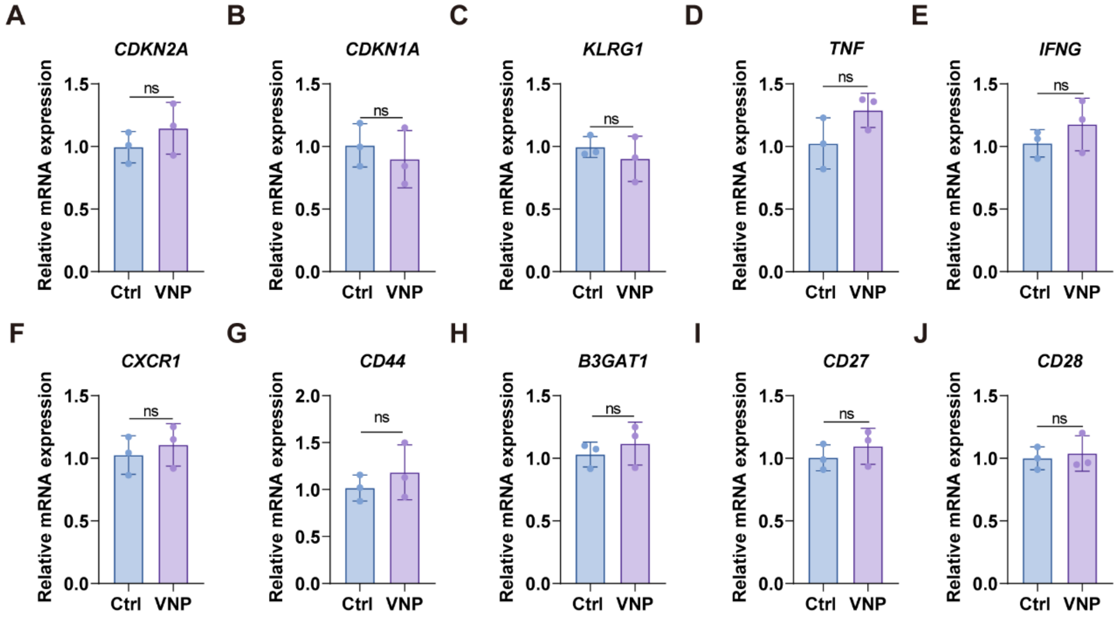

Figure S3 Effect of VNP on Jurkat cell senescence. (A–J) RT-qPCR was performed to quantify the transcriptional levels of senescence-associated marker genes in Jurkat T cells. Data represent the mean ± SD in (A–J) (n = 3). Statistical significance was determined using Student's t-test in (A–J).

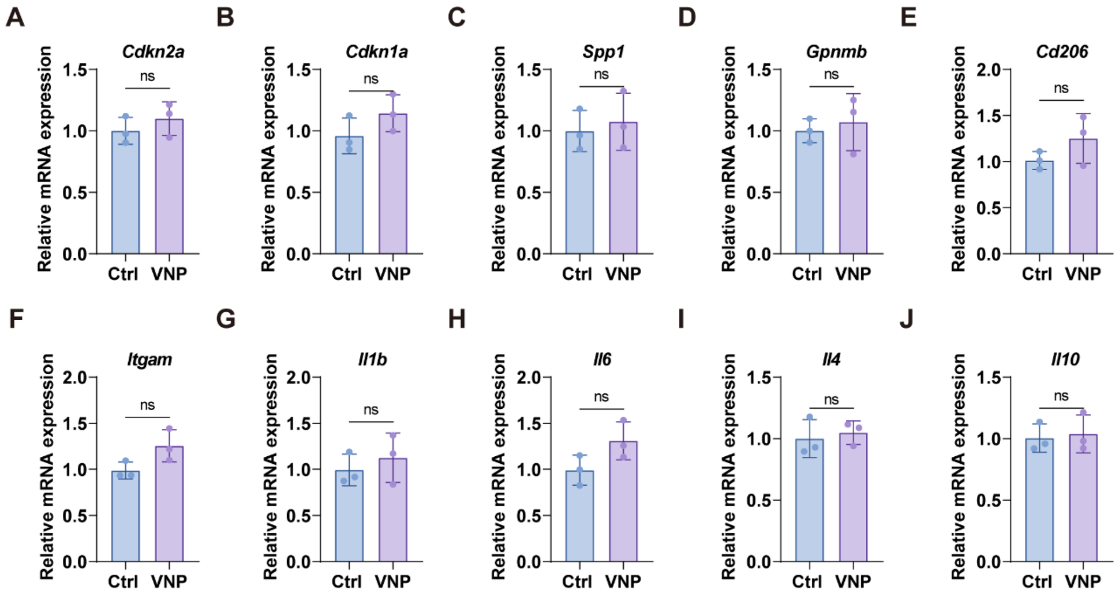

Figure S4 Effect of VNP on RAW264.7 cell senescence. (A–J) RT-qPCR was performed to quantify the transcriptional levels of senescence-associated marker genes in RAW264.7 cells. Data represent the mean ± SD in (A–J) (n = 3). Statistical significance was determined using Student's t-test in (A–J).

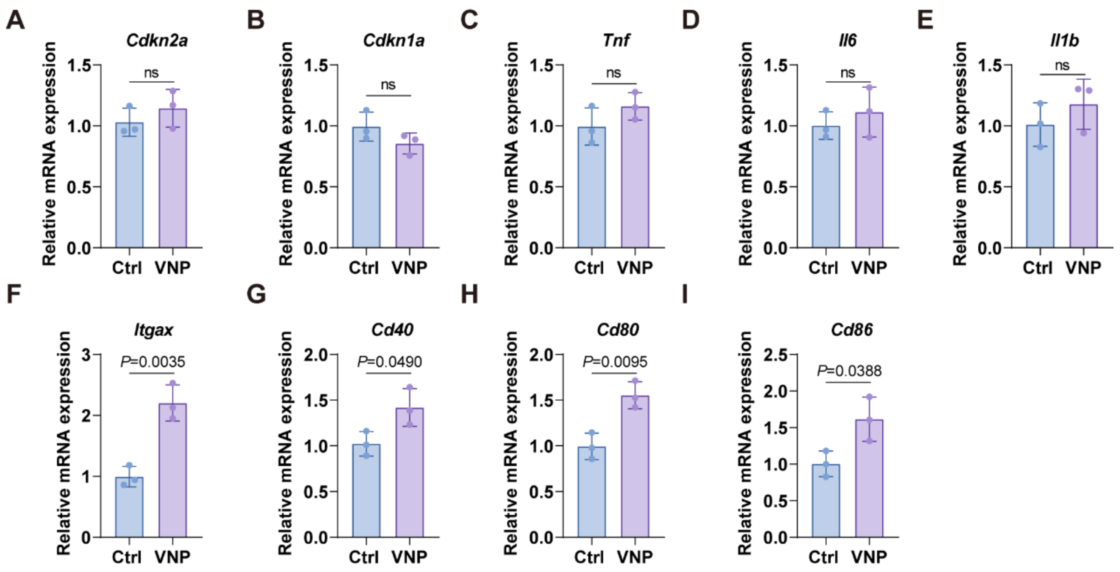

Figure S5 Effect of VNP on DC2.4 cell senescence. (A–I) RT-qPCR was performed to quantify the transcriptional levels of senescence-associated marker genes in DC2.4 cells. Data represent the mean ± SD in (A–I) (*n* = 3). Statistical significance was determined using Student's *t*-test in (A–I).

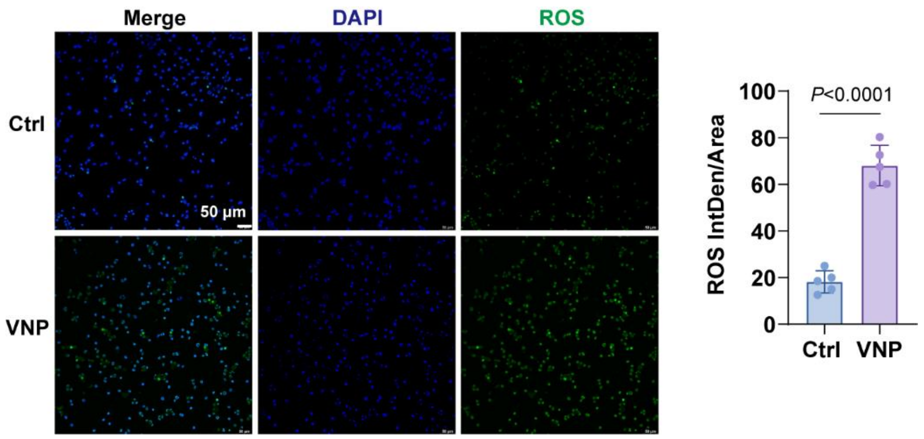

Figure S6 VNP significantly increased reactive oxygen species (ROS) production in neutrophils (n = 5). Scale bar = 50 μm. Data represent the mean ± SD. Statistical significance was determined using Student's t-test.

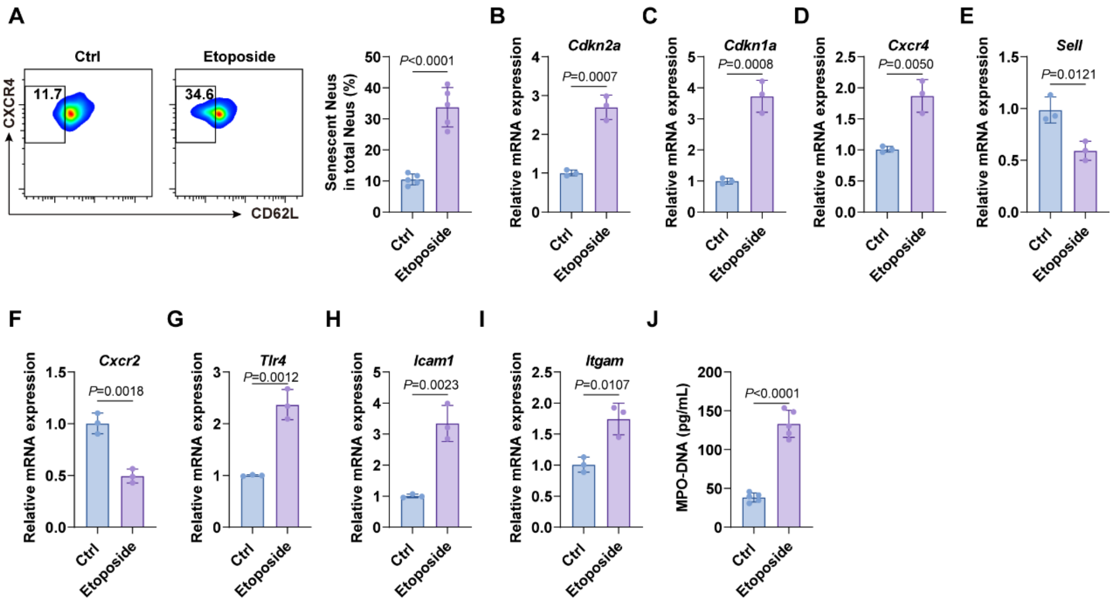

Figure S7 Etoposide-induced senescence of neutrophils leading to the formation of NETs. (A) FACS was used to assess etoposide-induced neutrophil senescence. (B–I) Quantitative PCR analyzed mRNA expression of senescence-associated marker genes in etoposide-exposed neutrophils. (J) ELISA quantification of MPO-DNA in the supernatant. Data represent the mean ± SD in (A, J) (n = 5) and (B–I) (n = 3). Statistical significance was determined using Student's t-test in (A–J).

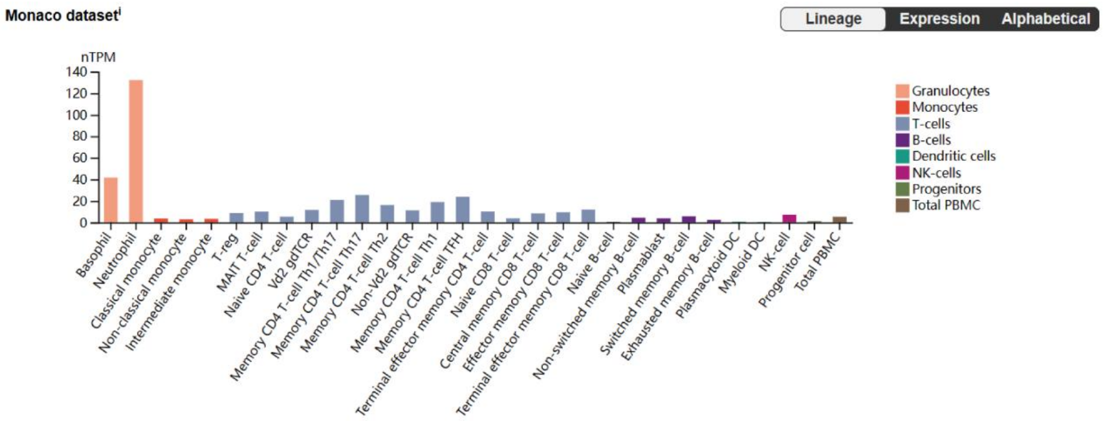

Figure S8 Analysis from the Human Protein Atlas (HPA) database. the expression profile of PD-L1 within immune cells in the HPA database.

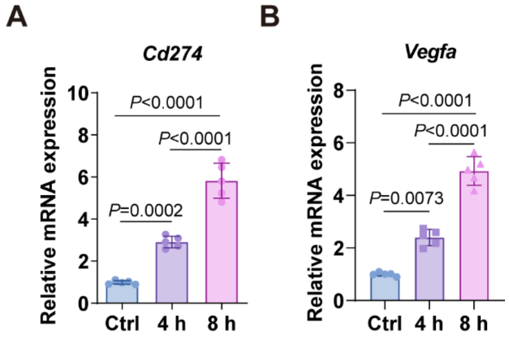

Figure S9 The transcriptional levels of PD-L1 and VEGF-*α* in neutrophils were examined by RT-PCR following co-incubation with VNP for different durations. (A, B) Relative mRNA expressions of PD-L1 (A) and VEGF-*α* (B) in neutrophils co-incubated with VNP. Data represent the mean ± SD in (A, B) (n = 5). Statistical significance was determined using one-way ANOVA with Tukey test in (A, B).

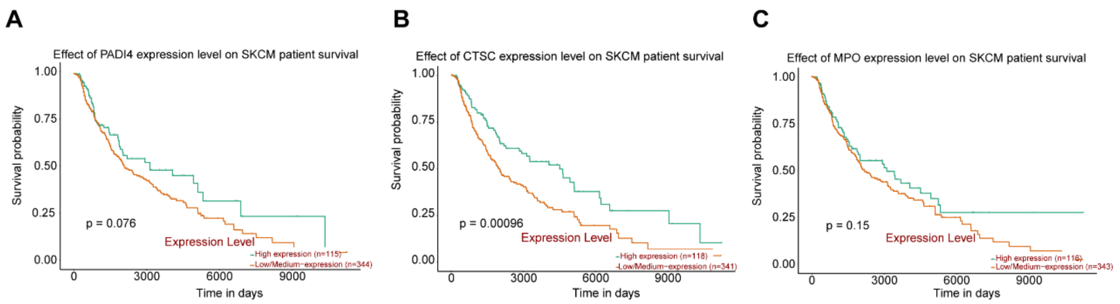

Figure S10 Analysis from the Cancer Genome Atlas (TCGA) database. (A–C) the effect of PADI4 (A), CTSC (B) and MPO (C) expression on the prognosis of SKCM patient survival.

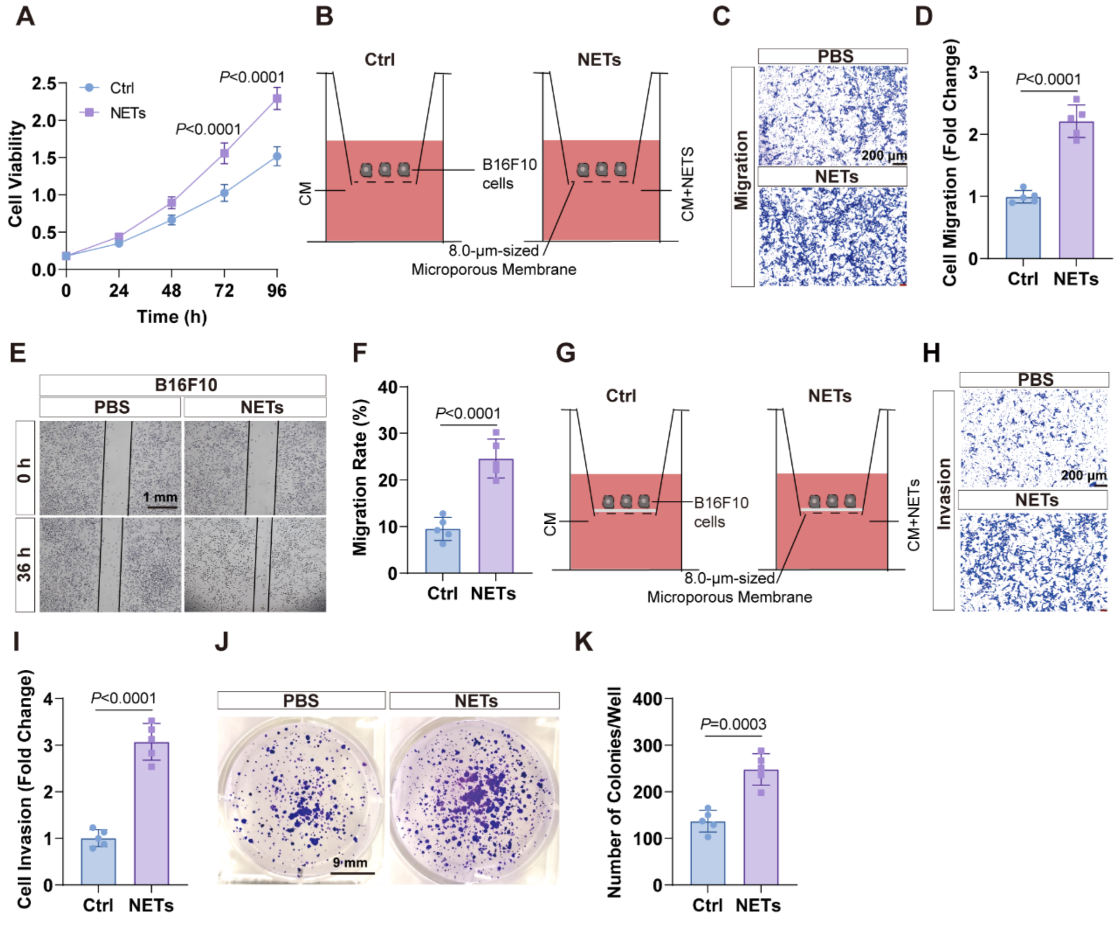

Figure S11 NETs promote B16F10 proliferation and migration. (A) B16F10 cells proliferation assay (CCK-8). (B–D) Trans-well migration assay. Scale bar = 200 µm. (E, F) Wound healing assay. Scale bar = 1mm. (G–I) Trans-well Matrigel invasion assay. Scale bar = 200 µm. (J, K) Colony formation assay. Scale bar = 9 mm. Data represent the mean ± SD. All data are representative of two independent experiments. in (A, D, F, I, K) (n = 5). Statistical significance was determined using Student's t-test in (D, F, I, K). Two-way ANOVA with Tukey's post hoc test was used in (A).

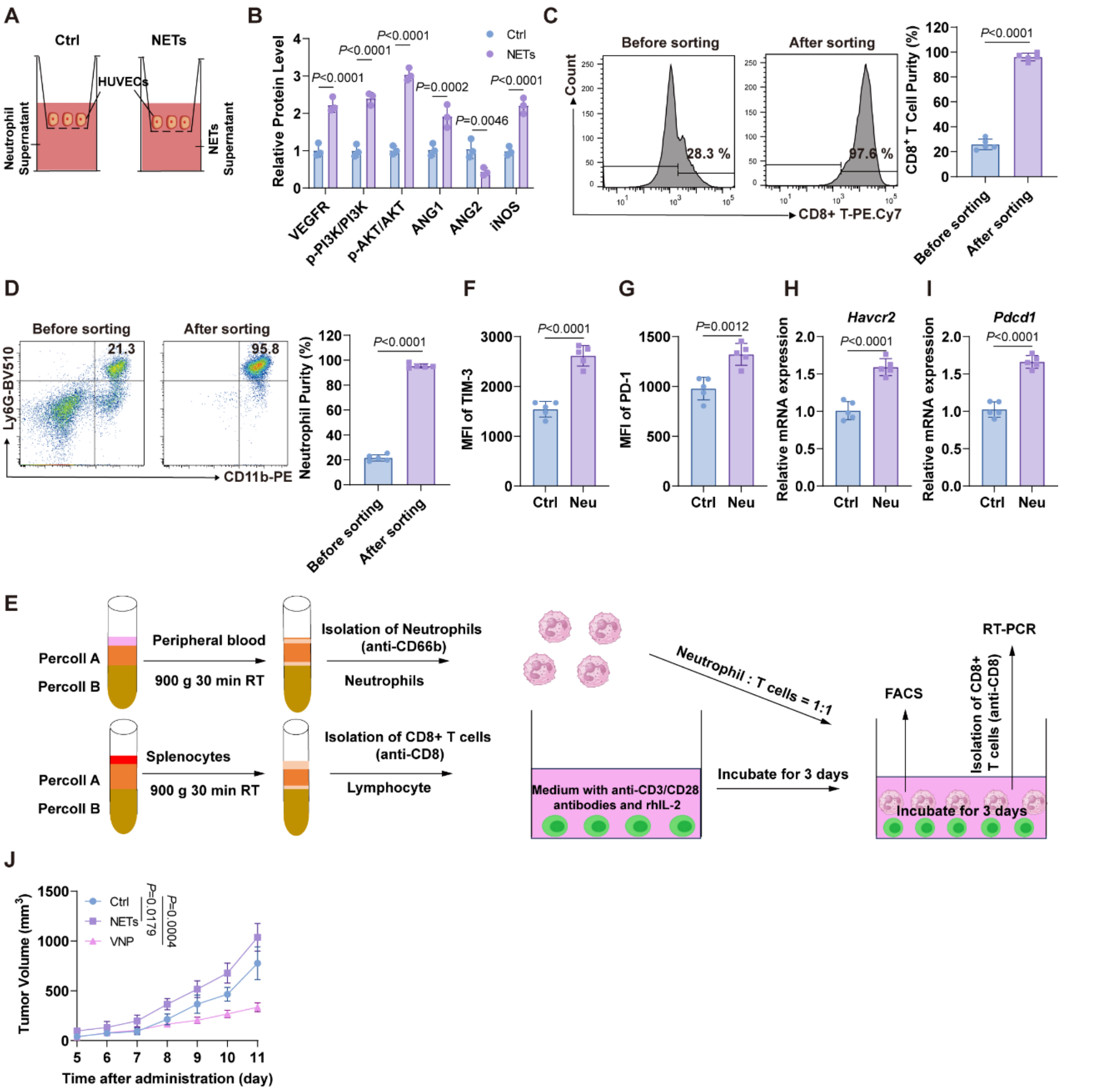

Figure S12 NETs promote T cell exhaustion and angiogenesis. (A) Schematic diagram of HUVECs migration stimulated with NETs. (B) Quantitative chart of proteins related to angiogenesis-associated genes. (C) FACS detected the purity of mouse splenic $CD8^+$ T cells before and after magnetic bead separation. (D) FACS detected neutrophil purity. (E) Schematic diagram of the co-culture experiment between neutrophils and T cells, including the extraction of neutrophils, T cells, and the activation of T cells. (F, G) MFI change of $CD8^+$ T exhaustion marker TIM-3 and PD-1. (H, I) RT-PCR to assess the transcriptional levels of $CD8^+$ T exhaustion. (J) Tumor growth curve. Data represent the mean ± SD in (C, D, F–J) (n = 5) and (B) (n = 3). Statistical significance was determined using Student's t-test in (C, D, F–I). Two-way ANOVA with Tukey's *post hoc* test was used in (B, J).

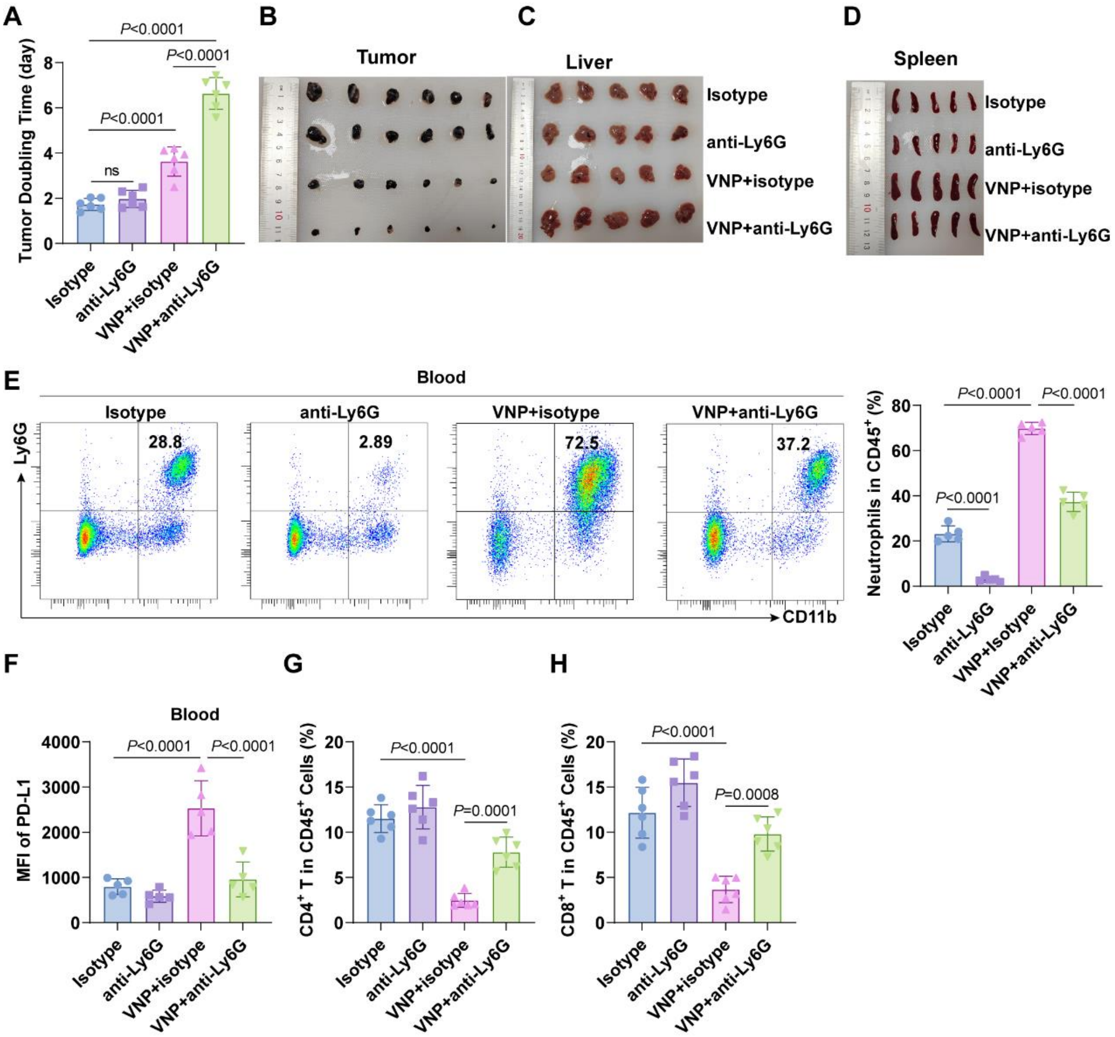

Figure S13 VNP combined with anti-Ly6G enhances anti-tumor effects. (A) Tumor doubling time. (B–D) Photographs of tumors (B), liver (C), and spleen (D). (E) FACS to detect changes in blood neutrophils in mice after different treatments. (F) MFI of PD-L1 in blood neutrophils. (G, H) Quantification of intra-tumoral $CD4^+$ (G) and $CD8^+$ (H) T cells as a percentage of $CD45^+$ cells. Data represent the mean ± SD. All data are representative of two independent experiments. in (A, B, G, H) (n = 6) and (C–F) (n = 5). Statistical significance was determined using one-way ANOVA with Tukey test in (A, E–H).

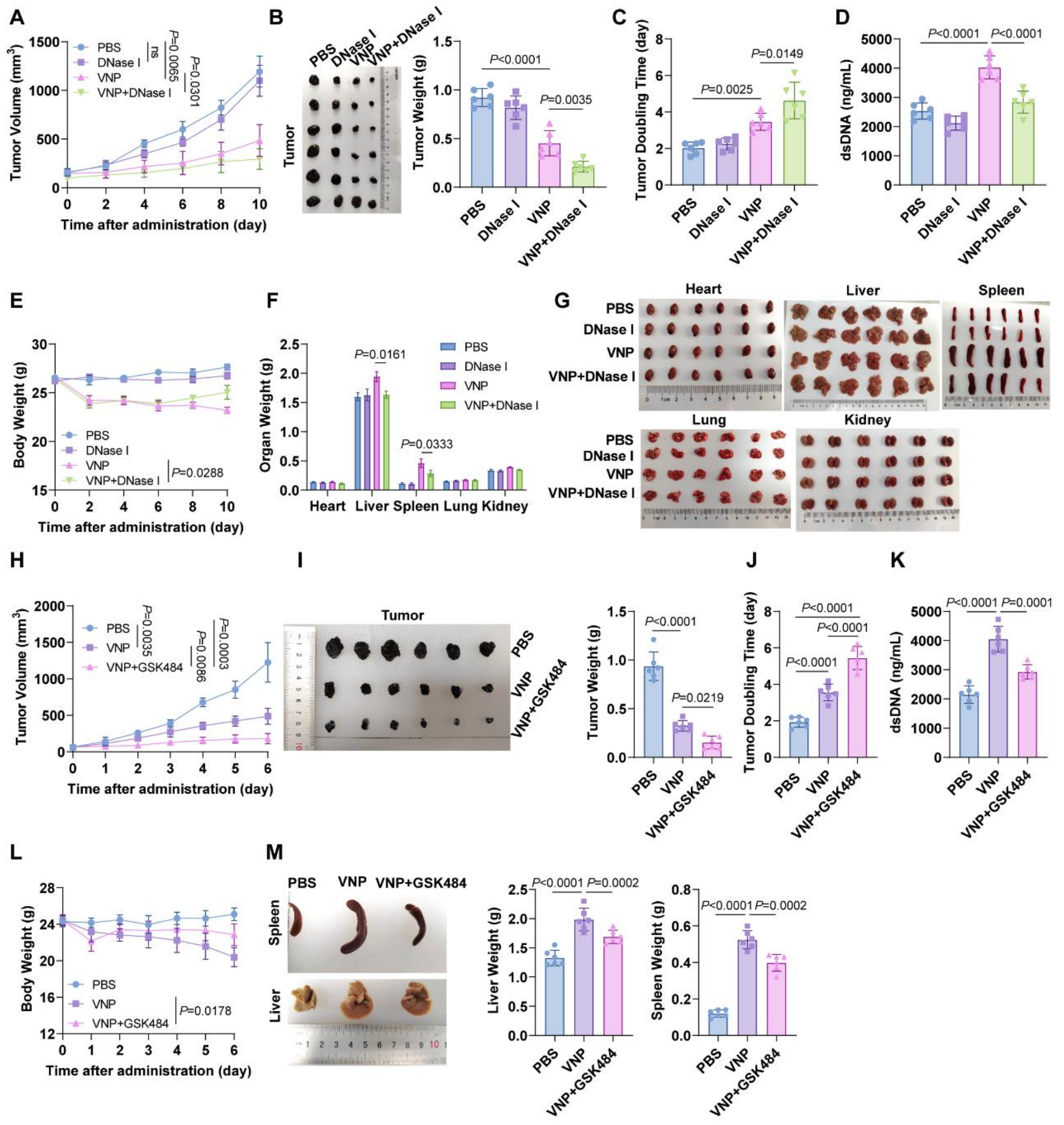

Figure S14 VNP combined with NETs blockers enhance anti-tumor effects. (A) The inhibitory effect of VNP combined with DNase I on tumor growth in B16F10 tumor-bearing mice. (B) Tumor weight. (C) Tumor doubling time. (D) Pico-green assay to detect changes of serum dsDNA. (E) Body weight changes daily. (F) Organ weight. (G) Photographs of mouse heart, liver, spleen, lung, and kidney. (H) the inhibitory effect of VNP combined with GSK-484 on tumor growth in B16F10 tumor-bearing mice. (I) Tumor weight. (J) Tumor doubling time. (K) Pico-green assay to detect changes of dsDNA in serum. (L) Daily trend of body weight. (M) Representative images of liver and spleen. Data represent the mean ± SD. All data are representative of two independent experiments. In

(A–M) (*n* = 6). Statistical significance was determined using One-way ANOVA with Tukey test in (B–D, I–K, M). Two-way ANOVA with Tukey's *post hoc* test in (A, E, F, H, L).

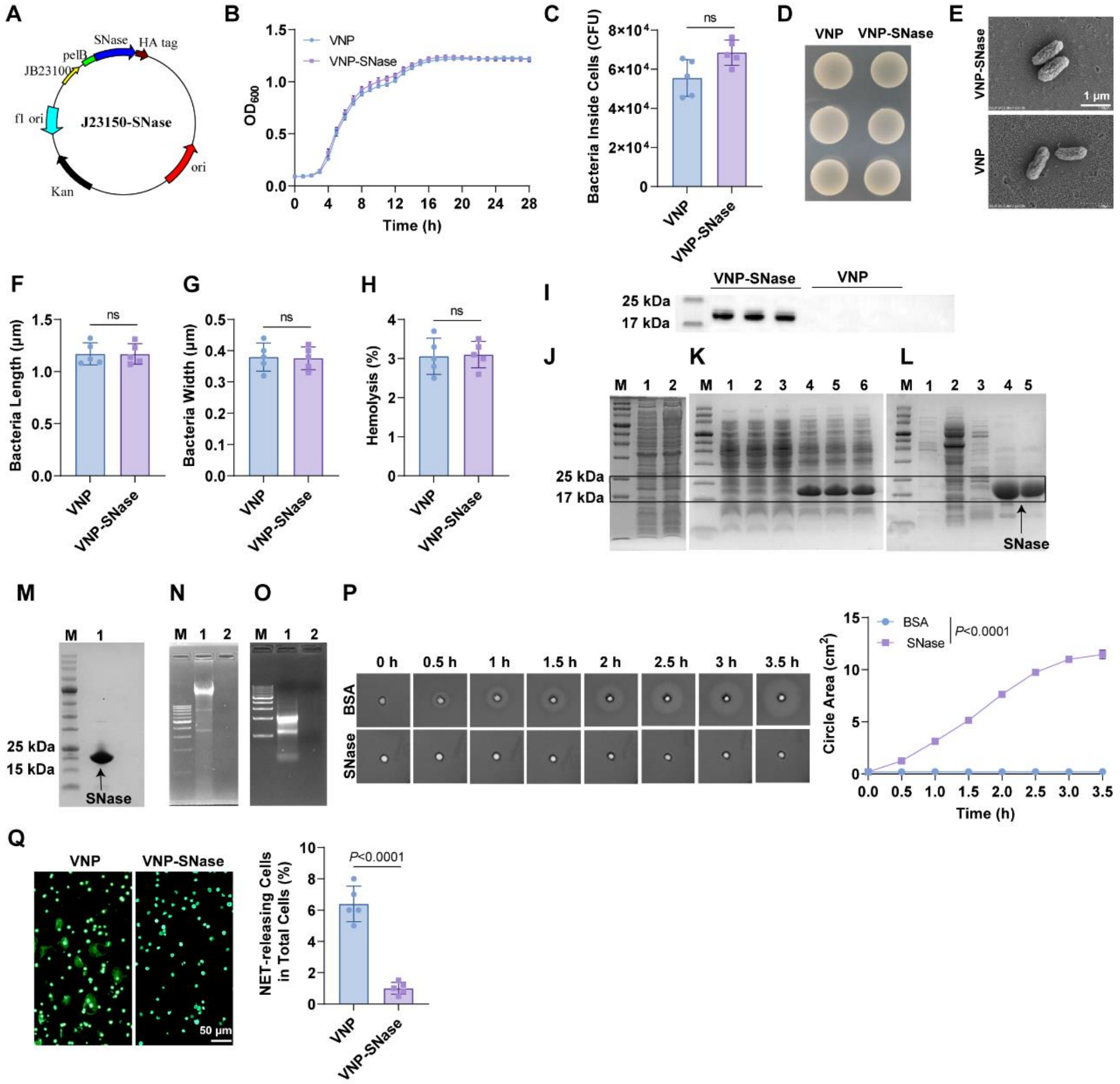

Figure S15 Construction of VNP-SNase and expression, purification, and activity verification of SNase. (A) Plasmid map of VNP-SNase. (B) Bacterial growth curves. (C) Tumor invasiveness. (D) Bacterial colony formation. (E) Bacterial morphology detected by SEM. Scale bar = 1 μm. (F, G) Quantitative analysis of VNP-SNase length and width. (H) Hemolysis assay demonstrating the *in vitro* safety profile of VNP-SNase. (I) Western blotting to verify the extracellular secretion of VNP-SNase. (J) SDS-PAGE to verify the expression of VNP-SNase, M (Marker), lane 1 (sample of VNP-SNase bacterial lysate preparation), lane 2 (sample of VNP bacterial lysate preparation). (K) Construction of *E. coli* BL 21-pET28a-His-SNase, IPTG induced expression, M (Marker), lanes 1–3 (samples of bacterial lysate preparation without IPTG induction), lanes 4–6 (samples of bacterial lysate preparation with IPTG induction). (L) After induction with IPTG at 18 °C for 8 h, sonication, centrifugation to obtain bacterial supernatant, and purification through a Ni column with different

concentrations of imidazole elution, M (Marker), lane 1 (flow-through), lane 2 (20 mmol/L imidazole eluate), lane 3 (70 mmol/L imidazole eluate), lane 4 (100 mmol/L imidazole eluate), lane 5 (200 mmol/L imidazole eluate). (M) Collection of 200 mmol/L imidazole eluate, desalting, freeze-drying, and SDS-PAGE to verify purity. (N) SNase can degrade DNA, M (DNA marker), lane 1 (genomic DNA of mouse B16F10 cell line), lane 2 (genomic DNA + SNase). (O) SNase can degrade RNA, M (DNA marker), lane 1 (total RNA of mouse B16F10 cell line), lane 2 (total RNA + SNase). (P) Toluidine blue-DNA agar method to detect SNase enzyme activity (left of the figure), and quantitative statistical analysis (right). (Q) VNP-SNase degrades NETs, with DNA staining (Sytox Green). Scale bar = 50 μm. Data represent the mean ± SD in (C, F–H, P, Q) (n = 5). Statistical significance was determined using unpaired Student's t-test in (C, F–H, Q). Two-way ANOVA with Tukey's post hoc test was used in (B, P).

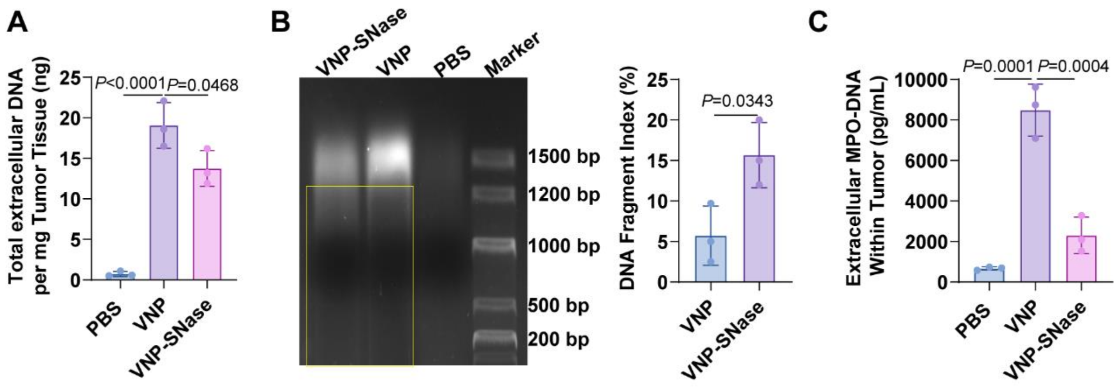

Figure S16 Specificity of VNP-SNase in degrading NETs. (A) Total extracellular DNA in tumor tissues of B1610-bearing mice on day 9 after bacterial treatment. (B) Agarose gel electrophoresis analysis of extracellular DNA fragmentation in tumor tissues after bacterial treatment. (C) ELISA analysis of the levels of the NETs marker MPO-DNA in tumor tissues. Data represent the mean ± SD in (A–C) (n = 3). Statistical significance was determined using One-way ANOVA with Tukey's test in (A, C), and using the unpaired Student's t-test in (B).

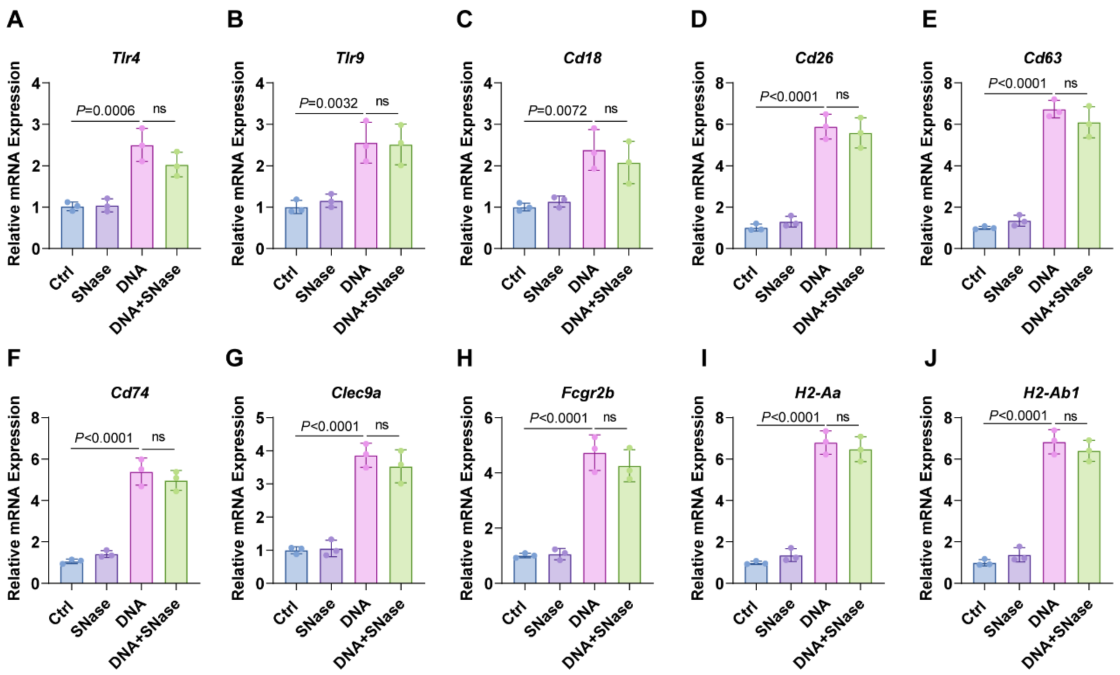

Figure S17 Effects of SNase on antigen uptake, processing, and presentation by DC cells *in vitro*. (A–J) RT-PCR analysis of the transcriptional levels of genes related to antigen uptake, processing, and presentation in DC cells treated with SNase. Data represent the mean ± SD in (A–J) (n = 3). Statistical significance was determined using one-way ANOVA with Tukey test in (A–J).

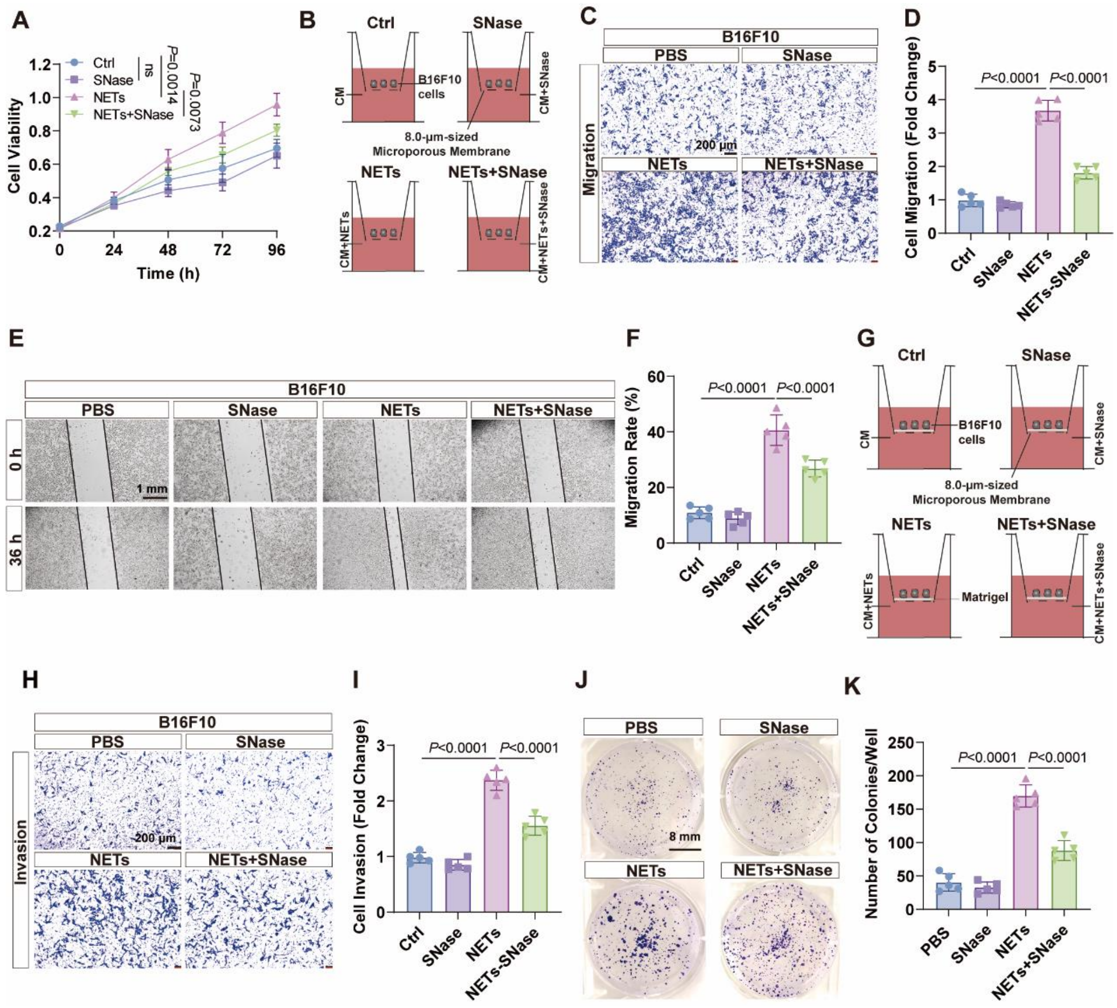

Figure S18 NETs promoted the proliferation, migration and invasion of B16F10 cells. (A) B16F10 cells proliferation assay (CCK-8). (B–D) Trans-well migration assay. Scale bar = 200 µm. (E, F) Wound healing assay. (G–I) Trans-well Matrigel invasion assay. Scale bar = 1 mm. (J, K) Colony formation assay. Scale bar = 8 mm. Data represent the mean ± SD. All data are representative of two independent experiments. in (A, D, F, I, K) (n = 5). Statistical significance was determined using one-way ANOVA with Tukey test in (D, F, I, K). Two-way ANOVA with Tukey's *post hoc* test in (A).

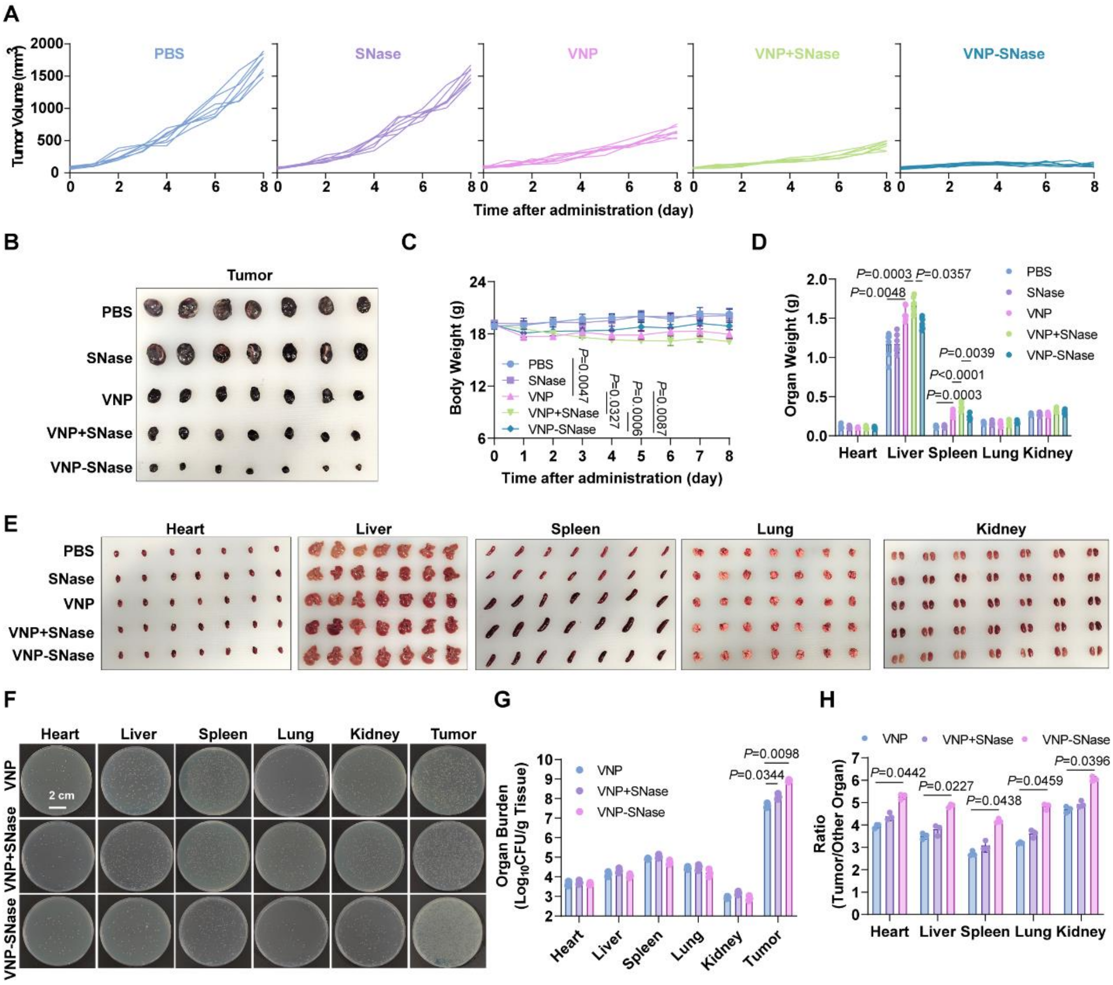

Figure S19 VNP-SNase enhances tumor targeting and ameliorates hepatosplenomegaly. (A) Tumor growth curves for each mouse in Fig. 6D. (B) Photographs of tumors. (C) Body weight. (D) Organ weight. (E) Photographs of major organs of mice. (F) *In vivo* biodistribution of VNP and VNP-SNase in murine major organs on Day 8. Scale bar = 2 cm. (G, H) Organ-specific bacterial burden (G) and tumor-to-organ ratio (H), quantified by colony-forming units (CFU) of VNP on Day 8. Data represent the mean ± SD in (A–E) (n = 7), (F–H) (n = 3). Statistical significance was determined using two-way ANOVA with Tukey's *post hoc* test in (C, D, G, H).

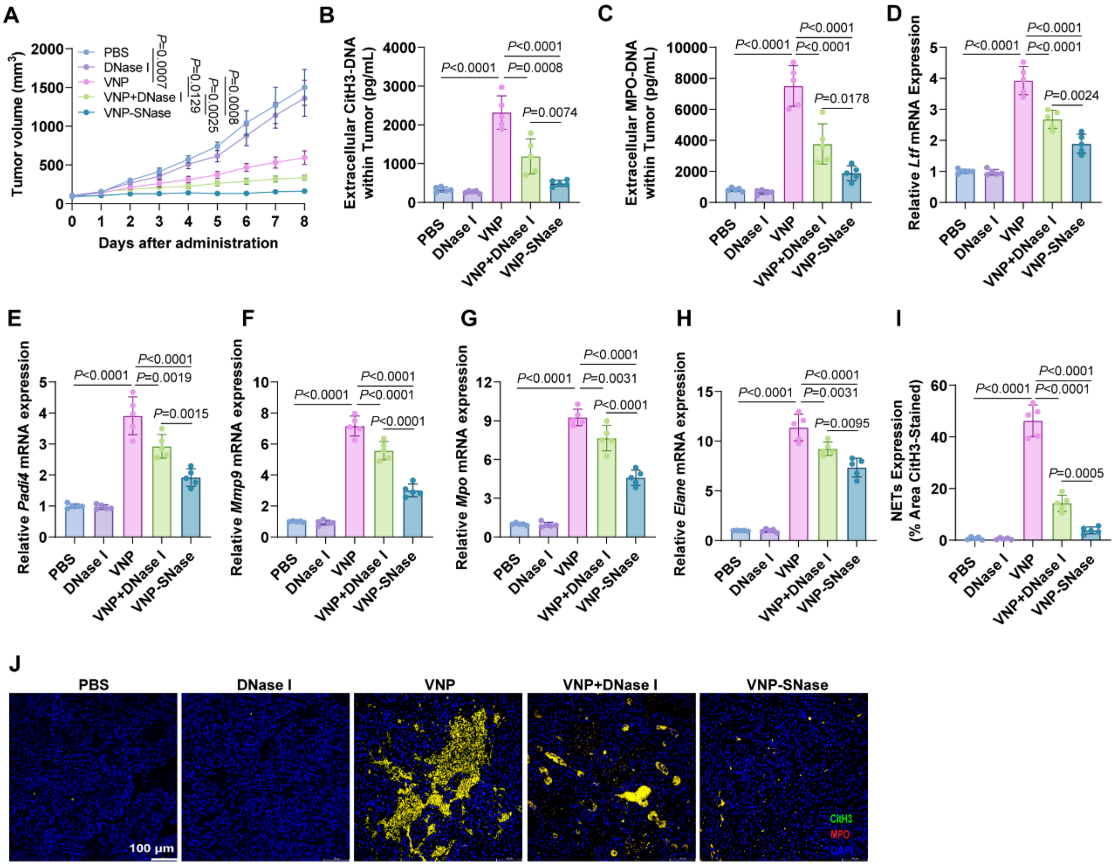

Figure S20 VNP-SNase is more effective than VNP combined with DNase I in degrading NETs. (A) Subcutaneous tumor volume changes in B16F10-bearing mice treated with VNP combined with DNase I or VNP-SNase. (B, C) Changes in the levels of extracellular NETs markers in tumor tissues after bacterial treatment. (D–H) RT-PCR analysis of transcriptional levels of NETs-related genes in mouse tumor tissues. (I, J) Immunofluorescence detection of NETs formation in tumor tissues. Scale bar = 100 μm. Data represent the mean ± SD in (A–I) (n = 5). Statistical significance was determined using two-way ANOVA with Tukey test in (A) and one-way ANOVA with Tukey test in (B–I).

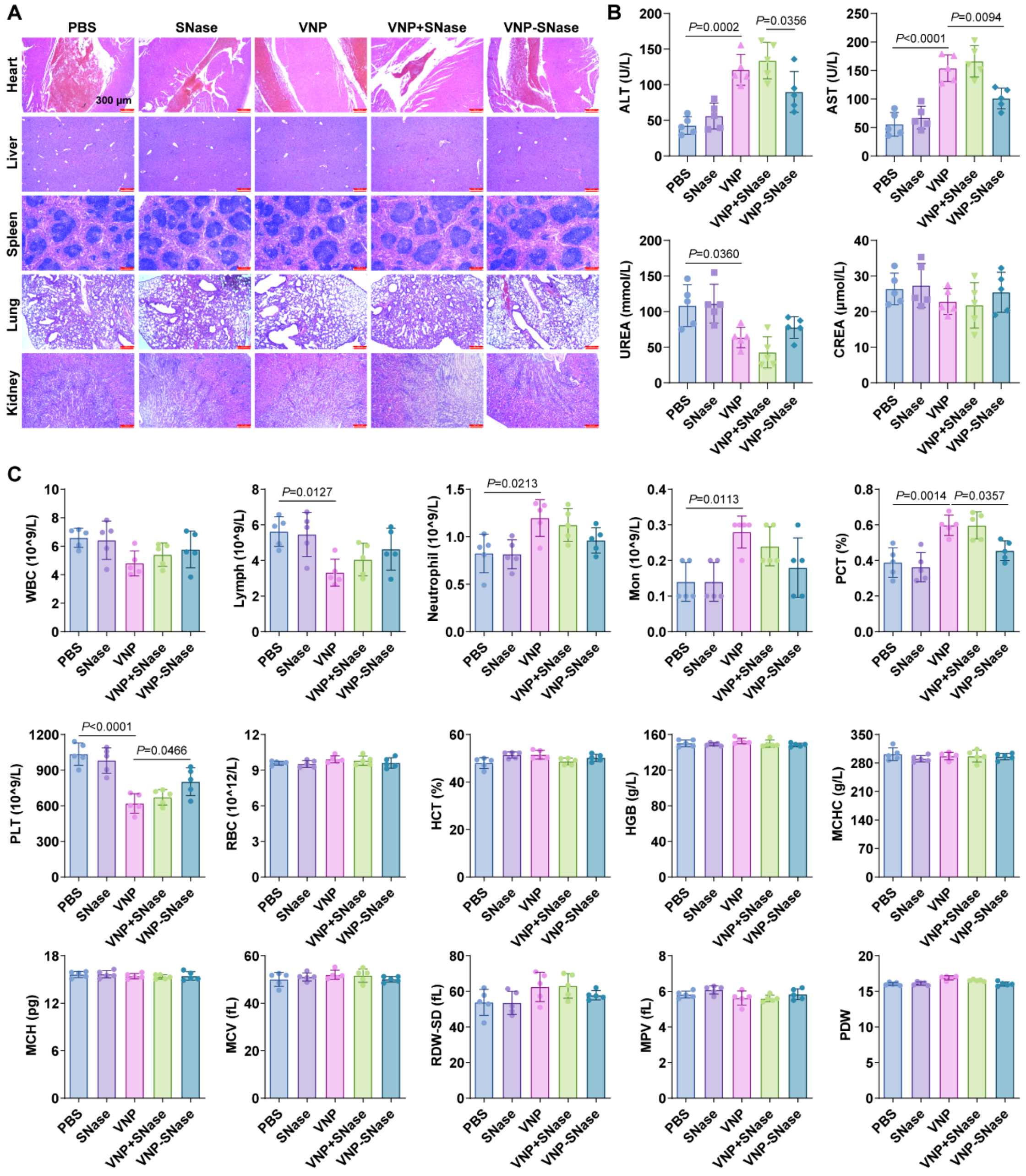

Figure S21 Safety assessment of VNP therapy. (A) Pathological analysis of organs by HE staining. Scale bar = 300 μm (B) Analysis of liver and kidney function indicators. (C) Complete blood count. Data represent the mean ± SD in (B, C) (*n* = 5). Statistical significance was determined using one-way ANOVA with Tukey test in (B, C).

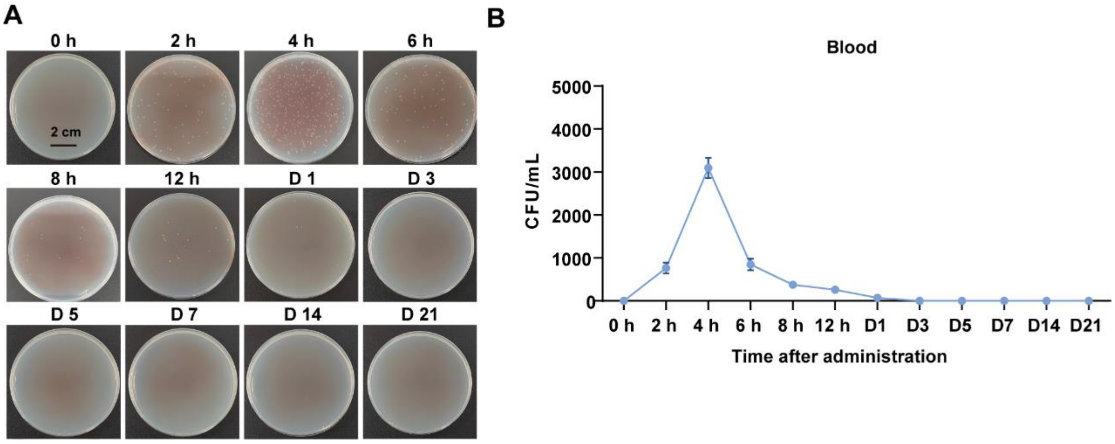

Figure S22 Pharmacokinetic Characterization of VNP-SNase in Systemic Circulation (n = 5). (A) Representative blood agar plate images from mice. (B) Quantification of kanamycin-resistant LB bacterial colonies. Scale bar = 2 cm. Data represent the mean ± SD in (A, B).

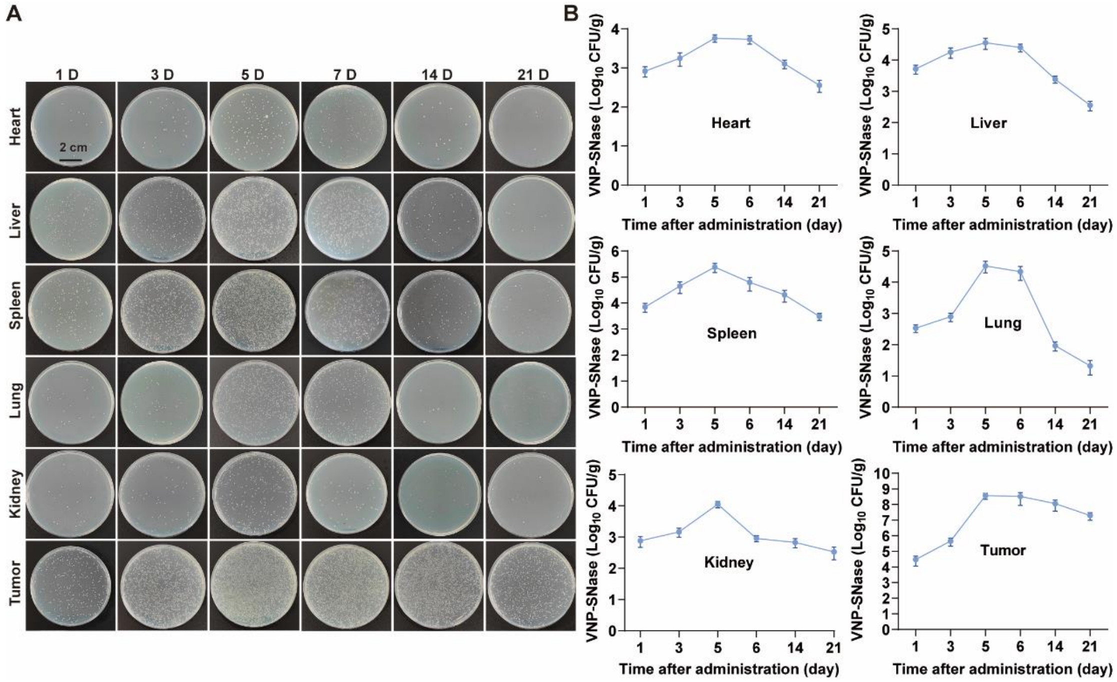

Figure S23 Tissue distribution and metabolic profiling of VNP-SNase in major organs of mice. Representative photographs (A) and quantification (B) of Bacterial colonization in major organs and tumors obtained from B16F10 tumor Bearing mice at different time points after intraperitoneal administration of engineered bacteria (*n*=3). Scale bar = 2 cm. Data represent the mean ± SD.

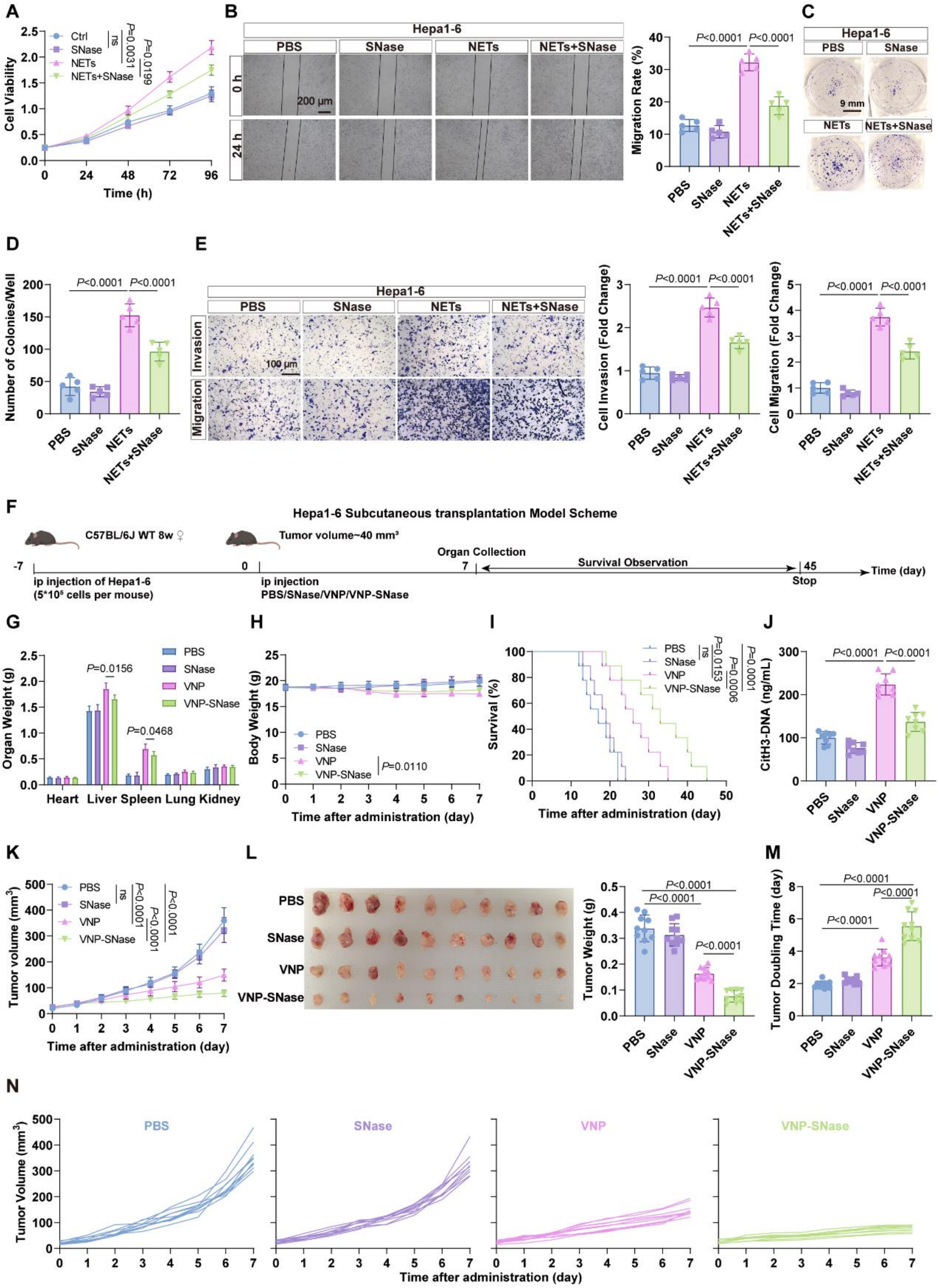

Figure S24 VNP-SNase inhibited the Hepa1-6 proliferation, migration and tumor growth caused by NETs. (A) Hepa1-6 proliferation assay. (B) Wound healing assay. Scale bar = 200 μm. (C, D) Colony formation assay. Scale bar = 9 mm. (E) Trans-well migration and invasion assay. Scale bar = 100 μm. (F) Schematic diagram of VNP-SNase antitumor efficacy against Hepa1-6 tumor-

bearing mice. (G) Organ weight. (H) Body weight. (I) Survival curve. (J) Changes of the NETs marker MPO-DNA in serum. (K) Tumor growth curve. (L) Photographs of tumor and tumor weight. (M) TDT. (N) Tumor growth curves for each mouse in (K). Data represent the mean ± SD. All data are representative of two independent experiments. in (A, B, D, E) (n = 5) and (I) (n = 9), (G, H, J–N) (n = 10). Statistical significance was determined using One-way ANOVA with Tukey test in (B, D, E, J, L, M). Two-way ANOVA with Tukey's *post hoc* test was used in (A, G, H, K). Log rank (Mantel–Cox) tests in (I).

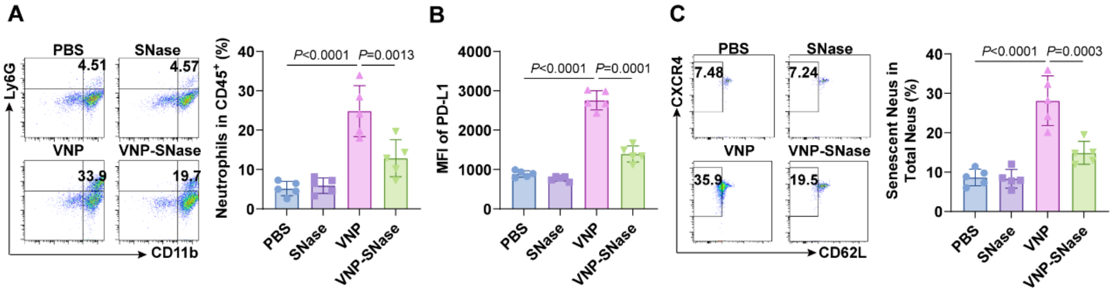

Figure S25 Effects of VNP treatment on tumor-associated neutrophils (TANs) at 3 days post-administration. (A) Frequency of neutrophils among CD45^{+} cells in tumors. (B) MFI of PD-L1 on neutrophils. (C) Percentage of senescent neutrophils within total TANs. Data represent the mean ± SD in (A–C) (*n* = 5). Statistical significance was determined using one-way ANOVA with Tukey test in (A–C).

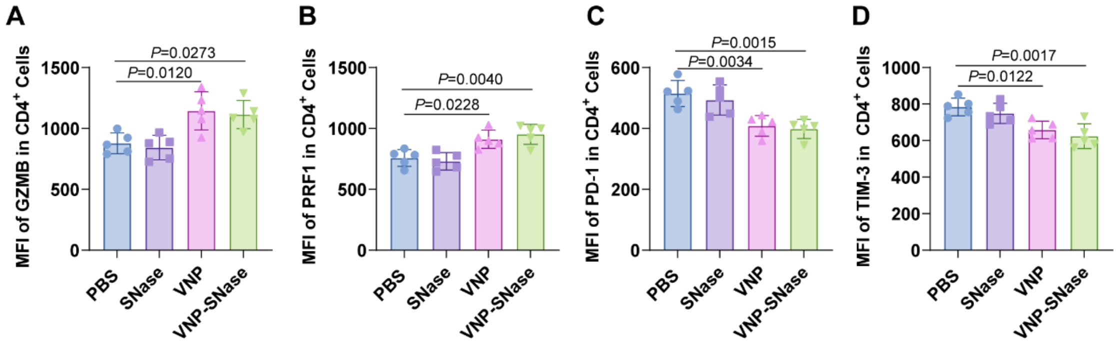

Figure S26 Effects of VNP treatment on CD4$^+$ T cells at 3 days post-administration. (A, B) MFI of GZMB and PRF1 on CD4$^+$ T cells within tumor. (C, D) MFI of PD-1 and TIM-3 on CD4$^+$ T cells within tumor. Data represent the mean ± SD in (A–D) (n = 5). Statistical significance was determined using one-way ANOVA with Tukey test in (A–D).

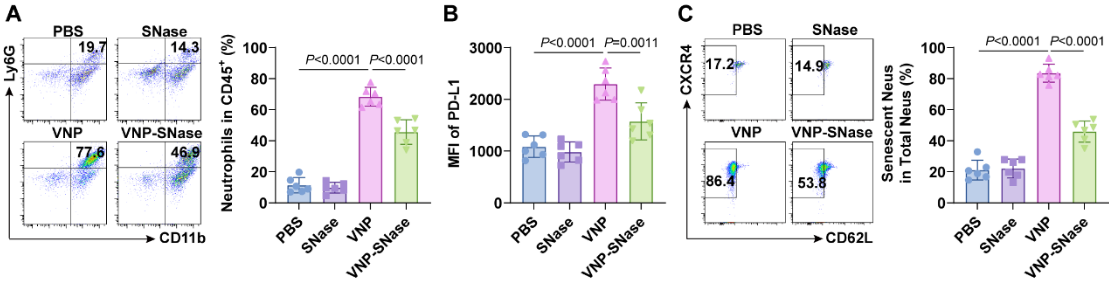

Figure S27 Effects of VNP treatment on TANs at 9 days post-administration. (A) Frequency of neutrophils among $CD45^{+}$ cells in tumors. (B) MFI of PD-L1 on neutrophils. (C) Percentage of senescent neutrophils within total TANs. Data represent the mean ± SD in (A–C) (n = 5). Statistical significance was determined using one-way ANOVA with Tukey test in (A–C).

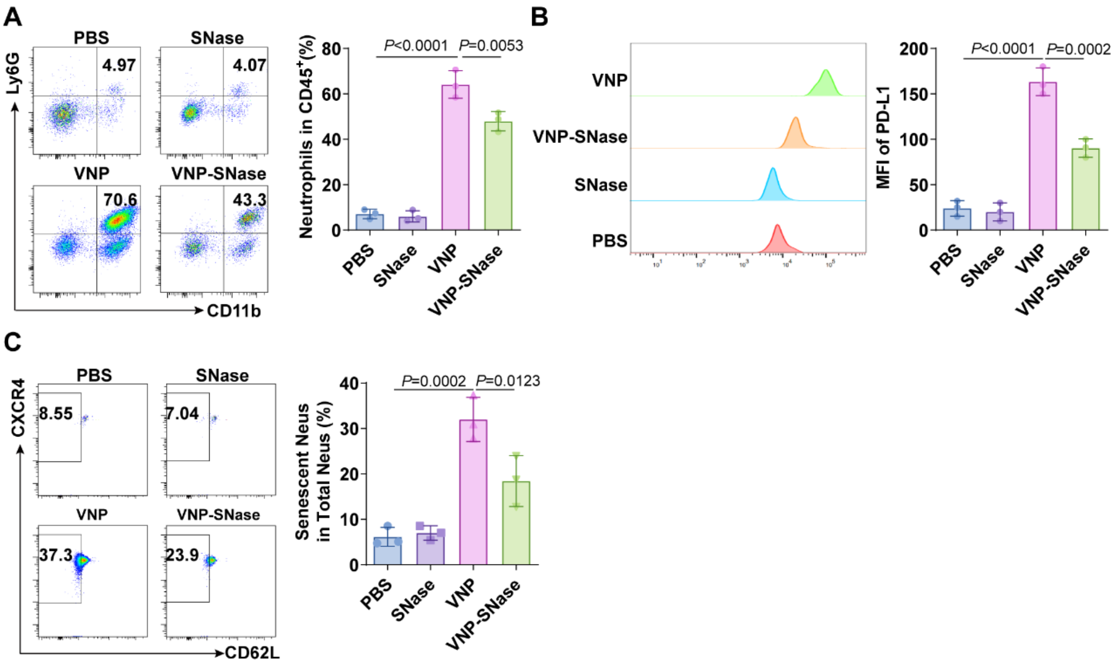

Figure S28 The impact of VNP treatment on blood neutrophils nine days post-administration. (A–C) the proportion of neutrophils (A), the proportion of PD-L1-expressing neutrophils (B) among immune cells in blood, and the proportion of senescent neutrophils among total neutrophils in blood (C). Data represent the mean ± SD. All data are representative of two independent experiments. In (A–C) (n = 3). Statistical significance was determined using one-way ANOVA with Tukey test in A–C.

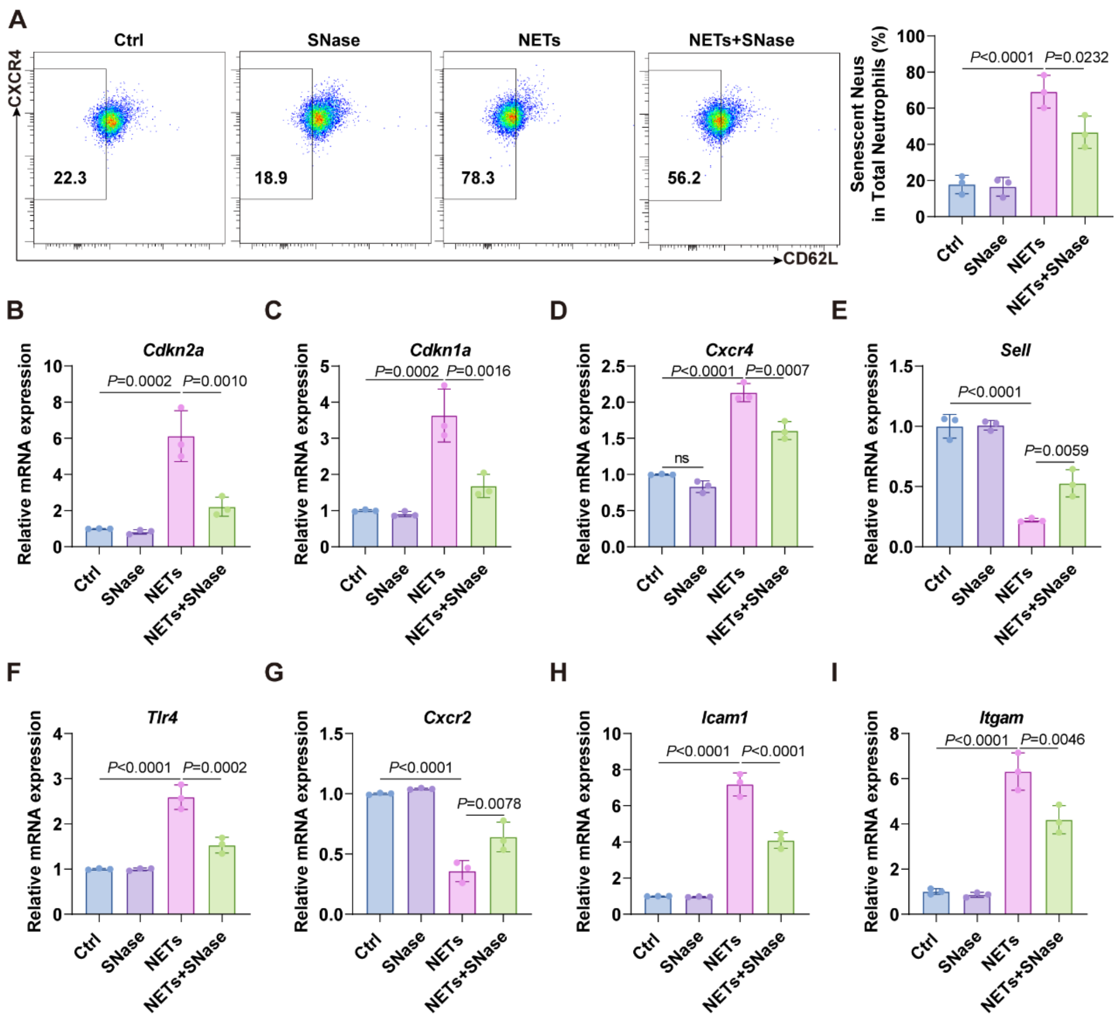

Figure S29 SNase mitigates NETosis-induced neutrophil senescence. (A) FACS was used to assess NETosis-induced neutrophil senescence. (B–I) Quantitative PCR analyzed mRNA expression of senescence-associated marker genes in NETosis-exposed neutrophils. Data represent the mean ± SD in (A–I) (n = 3). Statistical significance was determined using one-way ANOVA with Tukey test in (A–I).

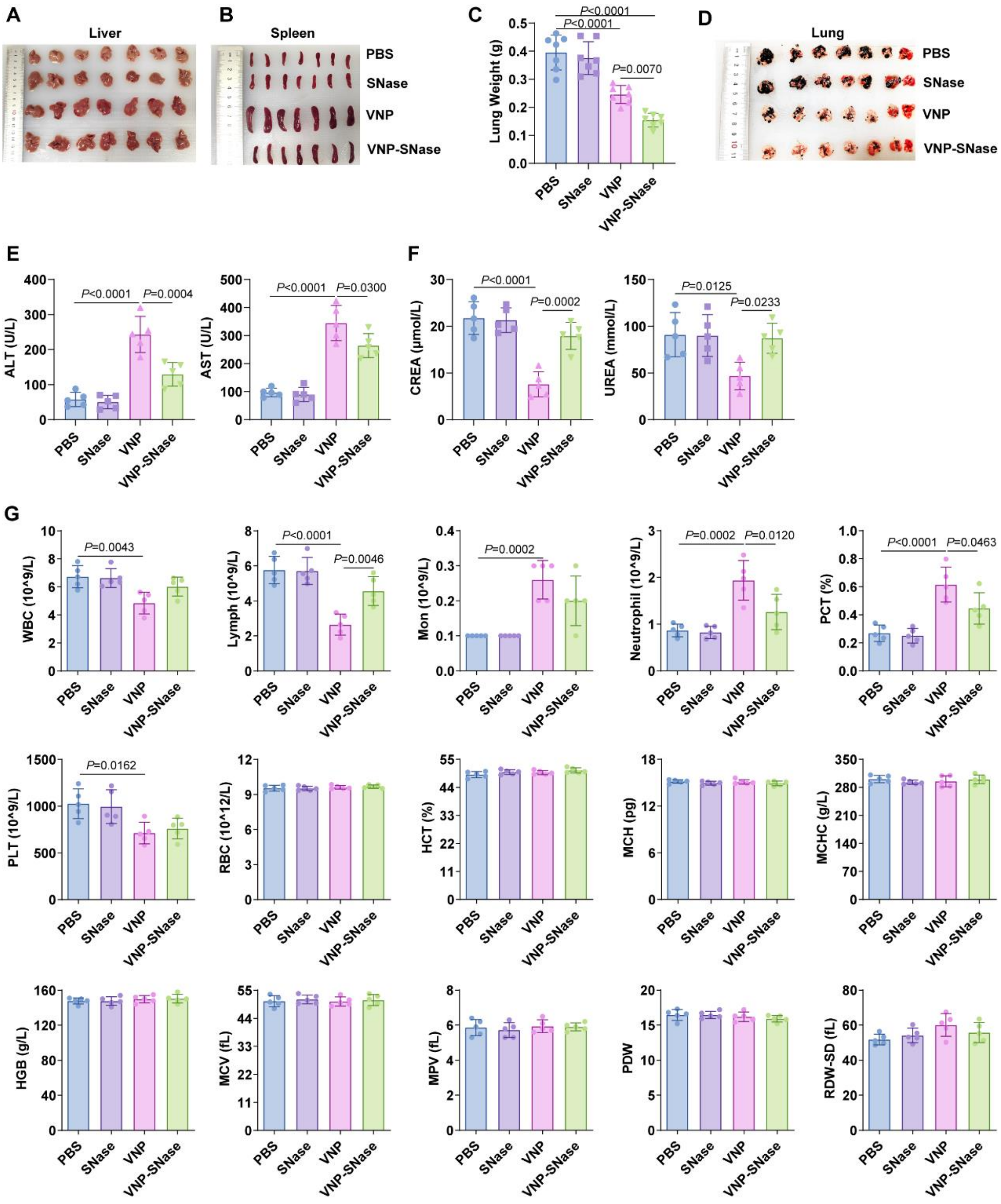

Figure S30 VNP-SNase more effectively inhibits melanoma lung metastasis. (A, B) Photographs of liver (A) and spleen (B). (C) Lung weight. (D) Photographs of lung metastatic foci. (E, F) Analysis of liver (E) and kidney (F) function indicators. (G) Complete blood count. Data represent the mean ± SD in (A–D) (*n* = 7) and (E–G) (*n* = 5). Statistical significance was determined using one-way ANOVA with Tukey test in (C, E–G).

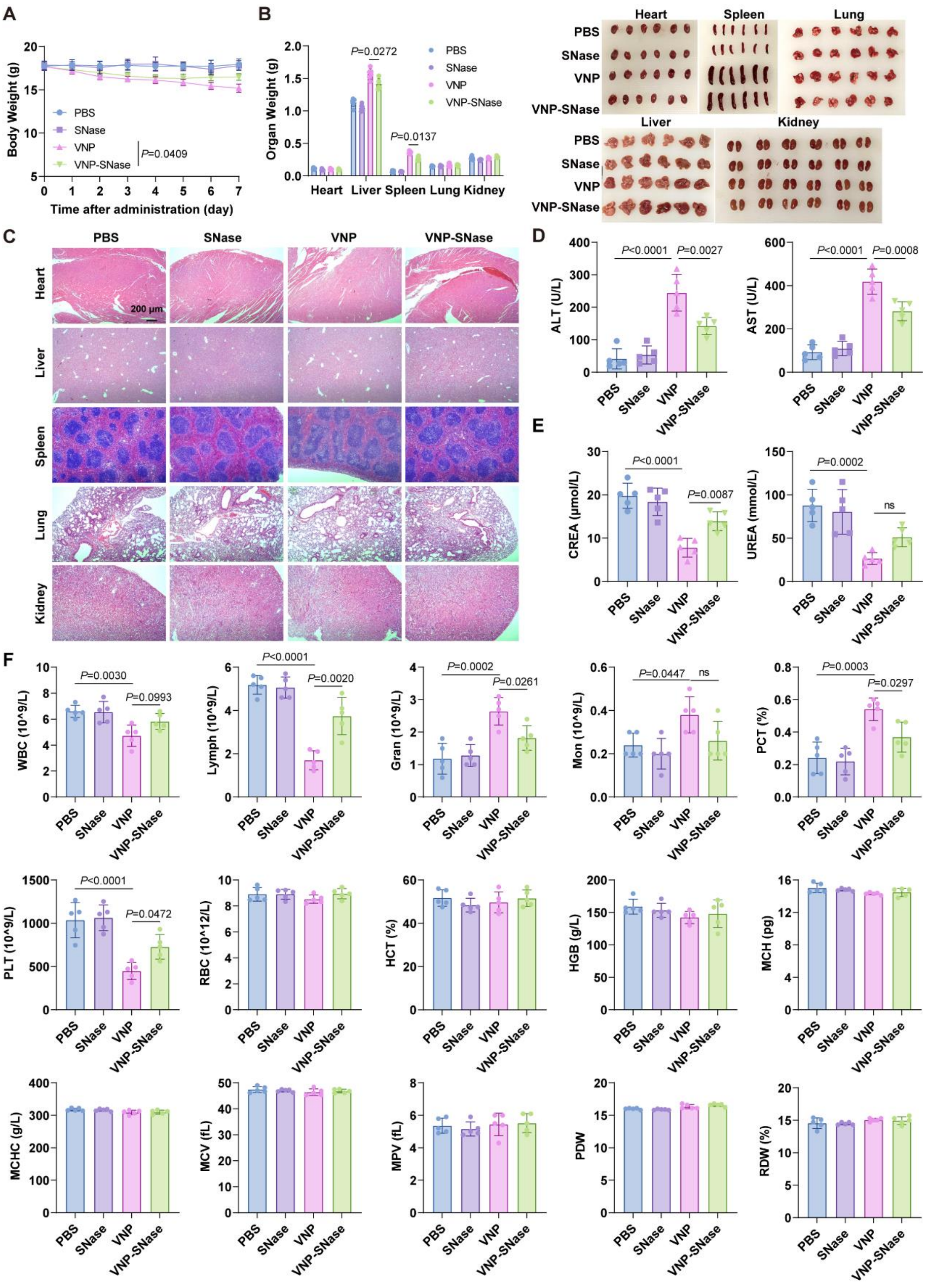

Figure S31 Safety evaluation of intravenous VNP-SNase in non-tumor-bearing mice. (A) Body weight. (B) Organ weight. (C) Pathological analysis of organs by HE staining. Scale bar = 200 μm. (D) Analysis of liver function indicators. (E) Analysis of kidney function indicators. (F) Complete blood count. Data represent

the mean ± SD in (A–F) (n = 5). Statistical significance was determined using One-way ANOVA with Tukey test in (D–F). Two-way ANOVA with Tukey test in (A, B).

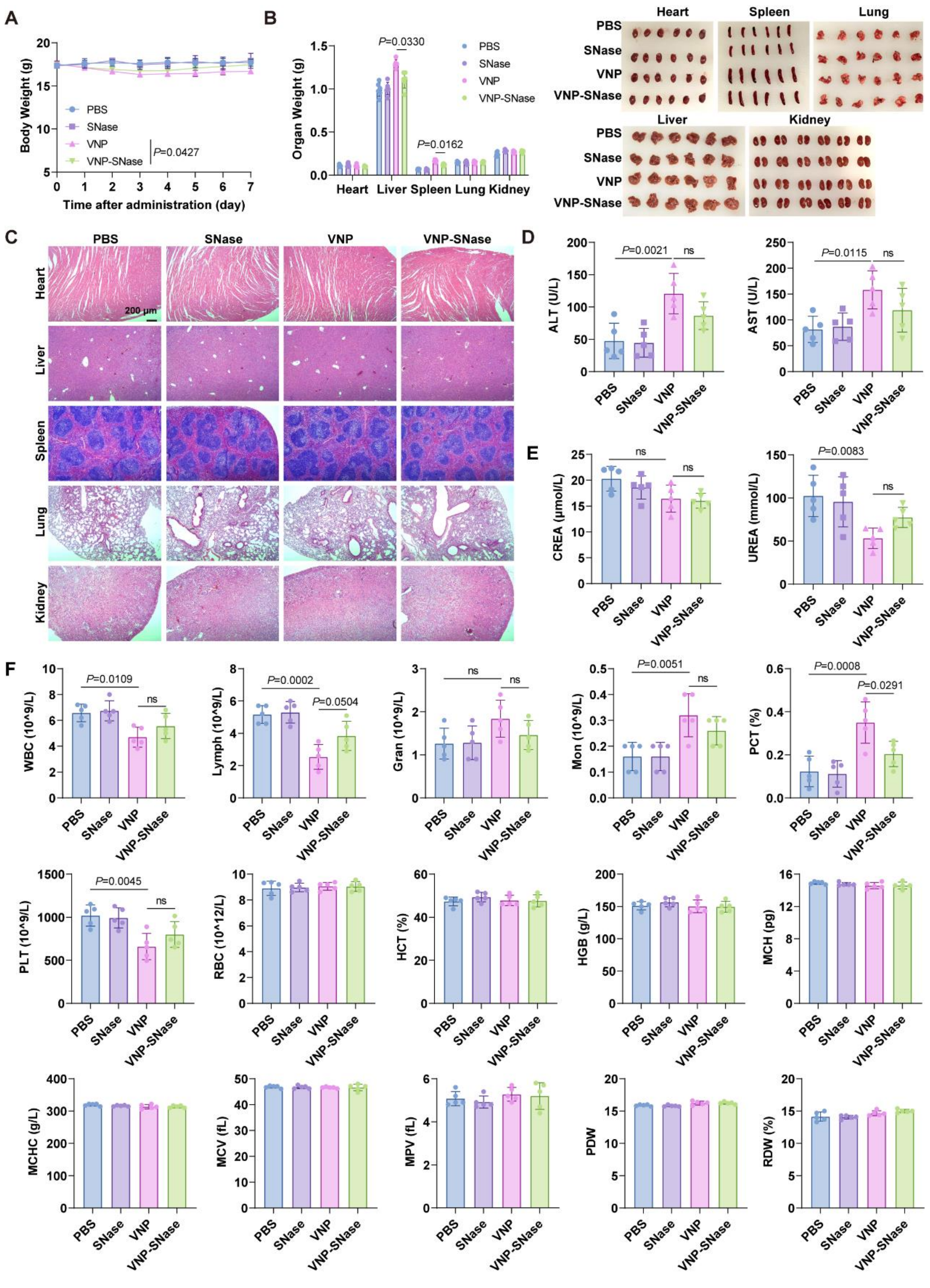

Figure S32 Safety assessment of intraperitoneal VNP-SNase in non-tumor-bearing mice. (A) Body weight. (B) Organ weight. (C) Pathological analysis of organs by HE staining. Scale bar = 200 μm. (D) Analysis of liver function indicators. (E) Analysis of kidney function indicators. (F) Complete blood count. Data represent the mean ± SD. All data are representative of two independent experiments. in

(A–F) (n = 5). Statistical significance was determined using One-way ANOVA with Tukey test in (D–F). Two-way ANOVA with Tukey test in (A, B).

Table S1. The primer sequence of RT-PCR.

Primer	Forward (5′–3′)	Reverse (5′–3′)
Ccl2	ACTGAAGCCAGCTCTCTCTTCCTC	TTCCTTCTTGGGGTCAGCACAGAC
Mmp9	GCGTCATTCGCGTGGATAAG	TGGAAACTCACACGCCAGAA
Tnf	GCCACGGCACAGTCATTGA	TGCTGATGGCCTGATTGTCTT
Il1b	TTCAGGCAGGCAGTATCACTC	GAAGGTCCACGGGAAAGACAC
Actb	ACTCTTCCAGCCTTCCTTCCT	CAGTGATCTCCTTCTGCATCC
Cdkn1a	TCTTGCACTCTGGTGTCTGA	CTGCGCTTGGAGTGATAGAA
Il6	TTGGTCCTTAGCCACTCCTTC	TAGTCCTTCCTACCCCAATTTCC
Cdkn2a	CAAGAGCGGGGACATCAAGACATC	CACAAAGACCACCCAGCGGAAC
Cxcl8	GCTGCTCAAGGCTGGTCCATG	CATCGTAGCTCTTGAGTGTCACAGG
Padi4	AAGGGCTACACAACCTTCGG	GCTGCTTTCACCTGTAGGGT
Elane	AGCAGTCCATTGTGTGAACGG	CACAGCCTCCTCGGATGAAG
Mpo	GCTCCTTGCCTGCCTCATTGG	TGCCAGTGTTGTCACAGATGATACG
Tlr4	ATGGCATGGCTTACACCACC	GAGGCCAATTTTGTCTCCACA
Itgam	CTGAACATCCCATGACCTTCC	GCCCAAGGACATATTCACAGC
Icam1	GGACCACGGAGCCAATTTC	CTCGGAGACATTAGAGAACAATGC
Cxcl2	CGCTGTCAATGCCTGAAG	GGCGTCACACTCAAGCTCT
Cd274	CAGGACGCAGGCGTTTACTG	CTTCCCACTCACGGGTTGGT
Cxcr2	ATGCCCTCTATTCTGCCAGAT	GTGCTCCGGTTGTATAAGATGAC
Cxcr4	GACTGGCATAGTCGGCAATG	AGAAGGGGAGTGTGATGACAAA
Ltf	GGACCGCAGGCTGGAACATC	ATCACCAATACACAGGGCACAGAG
Ccl6	TCTTTATCCTTGTGGCTGTCCTTGG	TGGGATCTGTGTGGCATAAGAGAAG
C5ar1	TTGATGCCACCGCCTGTATAGTC	ACACCAGATGGGCTTGAACACC
S100a8	ATGCCGTCTGAACTGGAGAAGG	AGTCATTCTTGTAGAGGGCATGGTG
S100a9	GCCAACAAAGCACCTTCTCAGATG	ATTCCTTCTTGCTCAGGGTGTCAG
Csf3r	GCAGGGTCCACCAACAGTACAG	CAGCAGAGCCAGGTCACTACAC
Ccl3	TCCCAGCCAGGTGTCATTTTCC	AGGCATTCAGTTCCAGGTCAGTG
Ccl4	CCCTCCCACTTCCTGCTGTTTC	AGCTGGCTTGGAGCAAAGACTG
Pdcd1	ACCCTGGTCATTCACTTGGG	CATTTGCTCCCTCTGACACTG
Havcr2	ACTGGTGACCCTCCATAATAACA	GCAGTTCTGATCGTTTCTCCA
Vegfa	CCACGACAGAAGGAGAGCAGAAG	ACAGGACGGCTTGAAGATGTACTC
Itgax	AGCCCATCTCCCTCCAGGTG	CACAAGCCAACAGCCAGGAAG
Cd86	TGCCGTGCCCATTTACAAAGG	TGTGCCCAAATAGTGCTCGTAC
Cd80	CTGCCTTGCCGTTACAACTCTC	CAGCAATGACAGACAGCACCAC
Cd40	GCGGTCCATCTAGGGCAGTG	TGGCTTGTCAGTCGGCTTCC
Il10	TTCCCTGGGTGAGAAGCTGAAG	ACCTGCTCCACTGCCTTGC
Il4	TGAACGAGGTCACAGGAGAAGG	AGCACCTTGGAAGCCCTACAG
Cd206	CACGGAGATCCACGAGCAAATG	CTGCCAACCACTGCGTACAC
Spp1	GAGCGGTGAGTCTAAGGAGTCC	GCTGCCCTTTCCGTTGTTGTC
Gpnmb	ATCGGCTGCCTGGCTGTG	TGCCCTTGACCGTGTTCCTG
CD28	TGGGTGCTGGTGGTGGTTG	GCCTGCTCCTCTTACTCCTCAC
CD27	GCTGCTCAGTGTGATCCTTGC	ACCAGAGTTACAGTGCCGACAG
CXCR1	GATTCCTCAAGATCCTGGCTATGC	AAGAGACATTGACAGACGAAGAAGTG
Ifng	AACCACTCCATTGTACCAACTCAC	GCACAGTTCTTAACCACCTTCAAATC
Il2	ACACAGCTACAACTGGAGCA	GCATCCTGGTGAGTTTGGGA
KLRG1	GGGCTTCTGACTGCAGTTCT	CAGCTGGCACAAGTGGAGTA
CD44	CTCCAGTGAAAGGAGCAGCA	GCAGGGATTCTGTCTGTGCT
B3GAT1	CGAAGCCAGGCCTACTTCAA	AGCCCTTCTTGCCCTCATTC
Itgam	CCACAGCCAGCGGATCATAGG	AGCCAGGTCCATCAAGCCATC
Tlr9	ACTTGATGTGGGTGGGAATTGC	AGATGGCTCAGGTGATGGAAGG
Cd18	AGGTCGGCAAGCAACTGATTTC	CCAGCAGCCTCGTGACATTG
Cd26	ACAGTGGCTCAGGAGGATTCAG	TGCTGCTGCTCGGATGGAC
Cd63	GCCATTGGTGTAGCGGTTCAG	GGAAGAGGAAGGCACCCACTG
Cd74	CATGGATGGCGTGAACTGGAAG	CTCGGTGGGCTTCTTCTCCTC
VEGFR2	AGGGAGTCTGTGGCATCTGAAGG	GTGGTGTCTGTGTCATCGGAGTG
ANGPT1	CGCTGCCATTCTGACTCACATAGG	CGTACTCTCACGACAGTTGCCATC
ANGPT2	ATGTGGTCCTTCCAACTTGAACGG	GCCTTGAGCGAATAGCCTGAGC
NOS2	CAGGGTGGAAGCGGTAACAAAGG	CCTGCTTGGTGGCGAAGATGAG
Clec9a	CTGTGTGGTCTGTATGGGCTTG	GCTGTGTCCTGTTGGATGAGTC
Fcgr2b	AATCTTGCTGCTGGGACTCATG	CAGTGTCACCGTGTCTTCCTTG
H2-Aa	CCCAACACCCTCATCTGCTTTG	ACCGTCTGCGACTGACTTGC
H2-Ab1	TGCTGTGGTGGTGCTGATGG	TGCGTCCCGTTGGTGAAGTAG

Table S2. The information of WB, IHC and IF antibody.

Antibodies	Article No.	Company
CD31	#77699	CST
MMP-9	#3852	CST
PADI4	#56308	CST
ATM	#2873	CST
P21	#2947	CST
P53	#32532	CST
Cyclin D	#55506	CST
p-RB	#8516	CST
HA-Tag	#3724	CST
β-Actin	#4967	CST
Ki-67	#9449	CST
VEGF-α	#50661S	CST
GADPH	#5174T	CST
PI3K	#4249T	CST
P-PI3K	#17366S	CST
AKT	#4691T	CST
P-AKT	#4060T	CST
MPO	AF3667	R&D systems
VEGFR	SU03-42	Huabio
ANG1	Bs-0800R	Bioss
ANG2	Bs-0766R	Bioss
iNOS	Bs-0162R	Bioss
CitH3	Ab5103	Abcam
CD62L	Bs-1036R	Bioss
CXCR4	AF5279	Affinity
ELANE	ET1702-78	Huabio

Table S3. The information of FACS antibody.

Antibodies	Article No.	Company
Ms CD45-APC-CY7	557659	BD Pharmingen™
Ms CD8a-PE-CY7	552877	BD Pharmingen™
Ms CD4-PE	561832	BD Pharmingen™
Ms CD274-BV421	568309	BD Pharmingen™
Ms CD11b-PE	553311	BD Pharmingen™
Ms CD62L-PE-CY7	560516	BD Pharmingen™
Ms Ly6G-BV510	740157	BD Pharmingen ™
Ms PD-1-BV650	569506	BD Pharmingen ™
Ms Gzmb-FITC	11-8898-82	eBioscience™
Ms TIM-3-BV786	568802	BD Pharmingen ™
Ms PRF-APC	17-9393-80	eBioscience™
Ms CXCR4-APC	306509	Biolegend™
Ms CD11c-APC	561119	BD Pharmingen ™
Ms MHC-II-PE	562010	BD Pharmingen ™
Ms CD86-FITC	561962	BD Pharmingen ™
Ms CD69-BV711	569691	BD Pharmingen ™
